# Targeting Vaccine Information Framing to Recipients' Education: A Randomized Trial

**DOI:** 10.1002/hec.70036

**Published:** 2025-09-17

**Authors:** Alice Dominici, Lisen Arnheim Dahlström

**Affiliations:** ^1^ Department of Economics European University Institute, IMT School for Advanced Studies, and Dondena Centre, Bocconi University Milan Italy; ^2^ Department of Medical Epidemiology and Biostatistics Karolinska Institute Stockholm Sweden

**Keywords:** education, HPV, information framing, vaccinations

## Abstract

We study the effect of framing informational campaigns scientifically or emotionally on the vaccination uptake of recipients with different educational backgrounds. 7616 Swedish mothers stratified by education received a leaflet on their children's upcoming HPV vaccination opportunity. The leaflet's framing was randomized between emotional and scientific, whereas the content remained uniform; control units received an uninformative reminder of the same length. We find substantial heterogeneity by educational background. Mothers with compulsory schooling exposed to scientific framing increased their uptake by 5.7 percentage points (7.25%). The effect was driven by less skeptical mothers with little previous HPV knowledge and higher engagement with the materials. Emotional framing decreased uptake by 4.8 percentage points (5.41%) among high school‐educated mothers who read more superficially and were more hesitant at baseline.

## 1 Introduction

Vaccine hesitancy was already a public health concern in 2019 (Dubé etal. 2021)^1^; COVID‐19 intensified it, increasing the volume of vaccine misinformation and eroding trust in science and health institutions (Eichengreen et al. 2021, 2024). As the risk of future pandemics remains high, contrasting vaccine hesitancy is a policy challenge of primary importance. While epidemiological studies recommend targeting vaccine communications,^2^ designing targeted informational campaigns remains challenging: how exactly recipients' background—especially education—influences vaccine information absorption is not yet fully understood. Health economics research finds that college‐educated mothers are more impacted by pseudo‐scientific disinformation (on the MMR, see Anderberg et al. 2011; Chang 2018), whereas epidemiological studies show that lower‐educated parents are more vulnerable to emotionally charged disinformation on social media (e.g., Yiannakoulias et al. 2019; Puri et al. 2019; Kearney et al. 2019; Hoffman et al. 2019). Therefore, framing vaccine information using scientific and emotional nudges could be the key to resolving the apparent inconsistencies between recipients' backgrounds and the absorption of vaccine information, and provide clearer indications for effective targeted campaigns.

This paper uses a pre‐registered stratified randomized controlled trial in Sweden to study the effect of scientifically and emotionally framed vaccine informational campaigns by recipients' education. We show these two types of framed information can have very different and non‐linear impacts in four strata defined by mothers' highest educational attainment: compulsory schooling, high school, undergraduate university, and postgraduate university education. We also include a fifth stratum comprised only of immigrants from Sweden and Europe's most representative immigrant communities. Non‐European immigrants consistently appear among the least vaccinated and more vulnerable populations to preventable diseases, due to higher vaccine hesitancy and lower access and utilization of health services (Dahlström et al. 2010; Wang et al. 2019; Azerkan et al. 2012; Møen et al. 2017). In other words, we investigate whether the framing techniques adopted by disinformation can be leveraged in the context of informational campaigns and whether the relationship between framing and recipients' backgrounds can be exploited in the same way.

Our focus is the Human Papillomavirus (HPV) vaccine. Recommended at age 12, it prevents several cancers, including cervical cancer, a common cause of death for women below age 45 in both developed and developing countries. Despite this, multiple waves of disinformation led to a 25% decrease in HPV vaccine uptake between 2019 and 2021 worldwide, a more significant decline compared to any other vaccine in the same period (The Economist 2023). Counteracting the disinformation behind this decline in uptake through effective informational campaigns poses a policy challenge in numerous countries.

We sent 7616 mothers a 650‐word leaflet covering the vaccine's safety and the consequences of catching cancer induced by HPV. While the framing is randomized, the informational content is homogeneous across treatments. The emotional framing leaflet (T1) conveys the information through the testimonies of local cancer survivors, whereas the scientifically framed leaflet (T2) uses medical and statistical jargon. A third group (the control, C) receives a leaflet that reminds of the upcoming vaccination possibility and contains some uninformative text of the same length, covering the history of the Swedish vaccination program. By comparing treatments to the control group, we subtract the effect of receiving an extra reminder and isolate the effect of framed information.

The informational content shifts the focus from the adverse effects of the vaccine to the consequences of HPV‐induced cancers, which are far more likely: cervical cancer alone, which is caused by HPV, is the fourth most common cause of women's deaths in the world, and in recent years HPV has been found to cause an increasing number of head‐neck cancers that also affect men (Chaturvedi et al. 2013).^3^ Our leaflets report that HPV‐induced cancers are transmitted sexually and are a serious threat at any age, contrary to the widespread misconception that they only affect older individuals. We emphasize that treatment for gynecological HPV‐induced cancers is invasive, that it often causes temporary or even permanent sterility, and that it significantly increases the risk of miscarriages. This contrasts the claims made by disinformation that vaccines adversely affect the fertility of both women and men. This type of disinformation is particularly effective and widespread among some immigrant communities that attach high value to their children's fertility and has been spread intensively in recent years, relative to COVID‐19 vaccines.^4^

Conducting the experiment in Sweden positively impacts our design in several ways. First, it allowed us to exploit administrative records to sample from the population and to measure actual vaccination choices as our primary outcome. While some recent studies used administrative vaccination records for COVID‐19 vaccines (e.g., Campos‐Mercade et al. 2021; Rabb et al. 2022; Dai et al. 2021), we do so for a childhood vaccination. Moreover, as we randomly select a large sample of mother‐child pairs (N=7616) and observe the primary outcome for each, our main results are not affected by selection, participation bias, or attrition, a common issue of survey‐based experiments in this literature. Since we also include a short survey to measure self‐reported intention to vaccinate, we can compare results using as outcomes the actual uptake (N=7616) and self‐reported intention to vaccinate on a much smaller sample (N=2204). The rich information contained in population registers also allows us to characterize survey respondents relative to the full sample. Moreover, childhood vaccinations are fully voluntary in Sweden and administered by school‐resident nurses during normal school time upon receipt of a signed consent form, which eliminates both the monetary and non‐monetary costs faced by parents when choosing to vaccinate their children.^5^ Finally, regarding trust in health institutions, Sweden is more comparable to the average Western European country than neighboring Nordic countries (Wellcome Trust 2021).

Within Sweden, we restricted our attention to Stockholm County in 2021. Although we cannot observe previous informational initiatives, we know they are usually coordinated at the county level. Focusing on one county ensures homogeneous exposure to pre‐treatment initiatives. The presence of Stockholm allows for observing a sufficient representation of mothers with a postgraduate education or an immigration background. In 2021, we were able to test the effect of framed information on boys' mothers: boys were included in the HPV vaccination program in 2020, and nearly all European countries are following. Finally, HPV vaccine uptake in Sweden remains sub‐optimal (below 90%), especially in Stockholm County and for boys (82.9% for the first dose in 2020). Interestingly for our research question, these figures hide a substantial heterogeneity by parents' educational level: the uptake in our control group (even after receiving an extra reminder as part of our study) ranges from 78.6% when mothers stopped at compulsory education to 93% when they pursued graduate studies.^6^

We adopt two strategies to maximize the credibility of our intervention and the resemblance to actual policies. First, we identify the sender as a joint venture between *Statistics Sweden* and *Karolinska Institute*, both reputable public institutions that played a key role in disseminating information and recommendations during the COVID‐19 pandemic. Second, we structure our leaflets as those normally handed out by Swedish health authorities for their campaigns.^7^ This also ensures that we are testing the effect of framing without introducing other novelties in the delivery mode of the information. Indeed, in Scandinavian countries, it is the norm for government agencies to send written materials to the recipients' home addresses.^8^ This mode of delivery allows authorities to have a nearly universal reach with minimal costs, but in the absence of address registries, targeted leaflets can be easily distributed in schools and other neighborhood‐based institutions to maximize coverage of specific target populations who might be harder to reach with costly online campaigns, especially in countries where school enrollment is bound by catchment areas, including the US, the UK, Australia, and New Zealand.

Our findings confirm that an overall null result hides substantial heterogeneity by educational background. Scientifically framed information increases uptake by 5.7 percentage points (7.25%) among mothers with compulsory education. However, emotionally framed information is counterproductive, as it lowers the uptake of mothers with a high school degree by 4.8 percentage points (5.41%). These are the additional effects of framing on top of an extra reminder, which is subtracted from our estimates. Yet, magnitudes are comparable to the reminder effects studied in the literature, underlying the potential for further improvements based on cost‐effective targeted information framing. In particular, our effect magnitudes align with reminder campaigns in Denmark (J. Hirani 2021; J. C. Hirani and Wüst 2024), where the HPV vaccine is also free, but HPV coverage is substantially lower, due to particularly wide diffusion of disinformation (Hansen and Schmidtblaicher 2019; Hansen et al. 2020), which implies more room for uptake increase compared to our Swedish setting.^9^ Our results confirm the negative educational gradient in the absorption of vaccine information highlighted by existing observational studies and provide new experimental insights into the relationship between education and the effectiveness of information framing. Framed information is instead not effective among immigrant mothers, whose reactions are mostly driven by baseline socioeconomic characteristics and country of origin.

Further insights come from causal forest estimates (Athey and Imbens 2016) and distinguishing by whether mothers replied to our first survey. Those who responded drove the positive effect of scientifically framed information. In contrast, among those who didn't reply, mothers with high school education drove the negative effect of emotional framing, whereas highly educated mothers had a positive effect, underlining again the importance of considering heterogeneity by education in assessing the effects of framing. We characterize respondents as having a more positive attitude toward vaccines at baseline and engaging more with our leaflets. For both positive and negative effects, estimates are larger for mothers who did not know HPV before our intervention: consistent with previous evidence (Bartoš et al. 2022), we argue that this is due to diminishing returns to informational campaigns. As a policy recommendation, this stresses the importance of planning only a few, highly effective campaigns: exposure to ineffective information can exhaust attention for future interventions and lead to null or potentially counterproductive effects. Indeed, our results highlight the importance of avoiding emotionally framed campaigns that use cancer survivor testimonies: while they are adopted by various health authorities, NGOs and advocacy groups worldwide, they can reduce uptake in a fairly large share of the population (19% in Stockholm County and up to 45% in Sweden).^10^ In terms of measurement, the positive effect of scientific framing is also found when the outcome is the self‐reported intention to vaccinate. However, the effect is understated with respect to actual vaccination uptake: this suggests that studies relying on self‐reported intention to vaccinate provide a lower‐bound estimate of the effect on actual vaccination uptake.

We contribute to a large and growing literature on vaccines in two ways. First, we add to the evidence on nudges by focusing on emotional and scientific framing. We do so for an important routine childhood vaccine, whereas the majority of recent works focus on COVID‐19, a unique case given the media coverage and the policies adopted to contrast the spread of the pandemic, and study the effect of nudging informational content rather than framing. One notable exception is Alsan and Eichmeyer (2024) –focused on American men with no college education—which uses homogeneous video messages on the flu vaccine, manipulating whether the informant is a doctor or a layperson: the latter reduces perceived social distance between the informant and the recipient and raises uptake more effectively.^11^ Moreover, our design isolates the effect of framed information from reminders, which have received considerable attention for both childhood and adult vaccinations (e.g., Milkman et al. 2022, 2021; J. Hirani 2021; J. C. Hirani and Wüst 2024). Second, we contribute novel experimental evidence with an explicit focus on the education of information recipients, previously studied observationally in the context of disinformation absorption (Anderberg et al. 2011; Chang 2018; Qian et al. 2020). Contrary to evidence from other fields (e.g., Antinyan and Asatryan (2019), who review the effect of nudges on tax payments), we show that vaccine‐related informational interventions can produce a behavioral response in low‐income recipients not only in the short term but also months after the initial intervention.^12^

The rest of the paper proceeds as follows. Section 2 introduces the institutional context and the data. Section 3 describes our framing intervention. Section 4 details our experimental design and outcome variables. Section 5 presents our main results, and Section 6 investigates heterogeneity using causal forests. Section 7 discusses the mechanisms of action of our intervention. Finally, Section 8 provides a general discussion and concludes the paper.

## 2 Background and Data

### 2.1 The HPV Virus and the Delivery of the HPV Vaccine in Sweden

There are about 100 types of HPV, 14 of which are high‐risk for cancer. HPV accounts for up to 90% of cervical cancers, a leading cause of death for women in Europe, and is linked to most vulvar, vaginal, anal, penile, and approximately 70% of oropharyngeal cancers in men.^13^ Nearly every sexually active adult is exposed to HPV in their lifetime (ECDC. European Centre for Disease Prevention and Control Report 2020; Viens et al. 2016; Chaturvedi et al. 2013). The Swedish vaccination program offers GARDASIL 9, which protects against the 9 most common cancer‐causing HPV types.

The diffusion of HPV and HPV‐induced cancers in Sweden is in line with the rest of Europe, with some cancers exhibiting higher incidence peaks (IARC 2019).^14^ In terms of vaccination uptake, the Swedish figure remains below the recommended value (90%), although the figure for girls is high relative to other European countries (Bruni et al. 2021). However, the 80% uptake of the two doses hides considerable heterogeneity by parents' education. As we report in Section 5 when presenting our results, education is associated with sizable changes in baseline uptake: this can be seen in our control group, even after receiving one extra reminder compared to the general population. In particular, our field area (Stockholm County) reports an average uptake of 78% for both doses, lower than other areas in the country (The Public Health Agency of Sweden, 2018). Relative to the average Western European country, Sweden is comparable or slightly worse in terms of trust in government, health agencies, and science and is more similar to the rest of Europe than to other Nordic countries that appear among the most trusting (Wellcome Trust 2021). More detailed background information on uptakes and trust indicators can be found in Supporting Information S1: Section A in the Appendix.

Within the Swedish vaccination program, children in fifth grade (11–12 years old) are offered the HPV vaccine free of charge directly in schools, between September and October. While this has been true for girls since 2010, boys have been included under the same conditions in 2020, in line with several other countries. All vaccinations in Sweden are fully voluntary: parents only need to express consent for the vaccination through a paper consent form (reported in Supporting Information S1: Section A in the Appendix), which also serves as a reminder and is handed out to students a few weeks before the scheduled vaccination. The consent form is only signed once to allow the receipt of both vaccine doses during the school year. The resident school nurse then administers the vaccine during normal school time, and chooses the specific day of inoculation autonomously, meaning that parents have no control over the timing of vaccination. Children who are absent on that day are vaccinated as soon as possible once they return to school. This ensures that the choice to vaccinate results from personal beliefs about the vaccine and is not confounded by monetary and non‐monetary costs.

### 2.2 Population and Administrative Data

Our population of interest is children (and their mothers) who were due to receive the HPV vaccine in the fall of 2021 in Stockholm County. We sample 7616 mother‐child pairs out of a population of 21,952.^15^ ^,^ ^16^ While we cannot observe previous informational campaigns, these are usually carried out at the county level by the *Public Health Agency of Sweden*: we restrict to Stockholm County to ensure uniformity of available information at baseline. Moreover, the city of Stockholm ensures a sufficient representation of both immigrant and highly educated parents.^17^ As detailed in Section 4, we stratify by mothers' country of origin and education level. For mothers born outside of Sweden, we restrict to those born in Eritrea, Iraq, Iran, Afghanistan, Somalia and Syria, as they are the most common non‐European immigrants in Sweden, and they are representative of numerous immigrant communities in other European countries.

For all 7616 children in the sample, we observe HPV vaccination records for the first dose (the primary outcome) from the registers held by the *Public Health Agency of Sweden*. Note that while we cannot access data on the second dose, this is automatically included in the authorization signed by parents at the beginning of the school year. Our secondary outcomes, namely self‐reported intention to vaccinate and beliefs on vaccines, are instead measured with a survey administered right after being exposed to the leaflet and are observed only among survey respondents. We also ran an endline survey in November after the HPV vaccination: it asked mothers whether they had authorized the vaccination and investigated possible mechanisms. The evidence from this second survey remains only suggestive due to the reduced sample size and high self‐selection (N=694). The main purpose of this second survey is to study the determinants of self‐selecting into replying, which we discuss for our mechanisms' analysis (Section 7).

Registry data also allows us to access detailed demographic and socioeconomic information on parents, covering income, capital income, occupation and education type. For children, we observe the most recent vaccination record before treatment (how many doses of the MMR vaccine they received 3 years before treatment). The full list of variables is presented in Supporting Information S1: Section C of the Appendix. The first survey (Supporting Information S1: Section F of the Appendix) complements this information by asking about pre‐treatment reception of information on the vaccine, preferred sources of information, intention to vaccinate, beliefs on vaccines, questions about personal networks and percentage of the informational leaflet read.

## 3 The Informational Intervention

**FIGURE 1:**
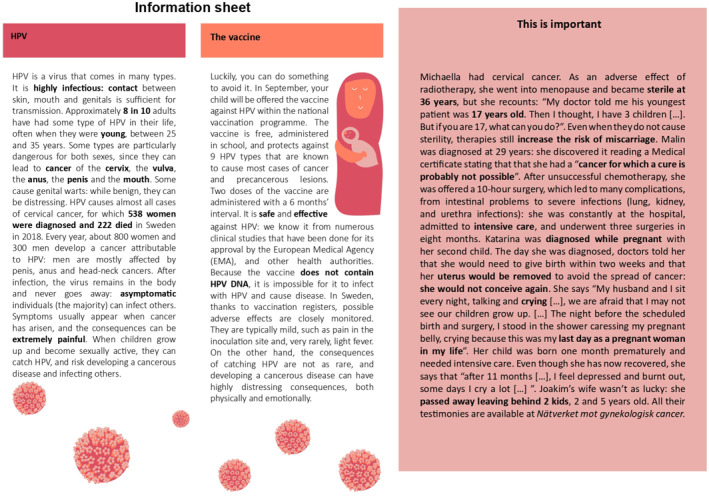
Emotionally framed leaflet (T1).

**FIGURE 2:**
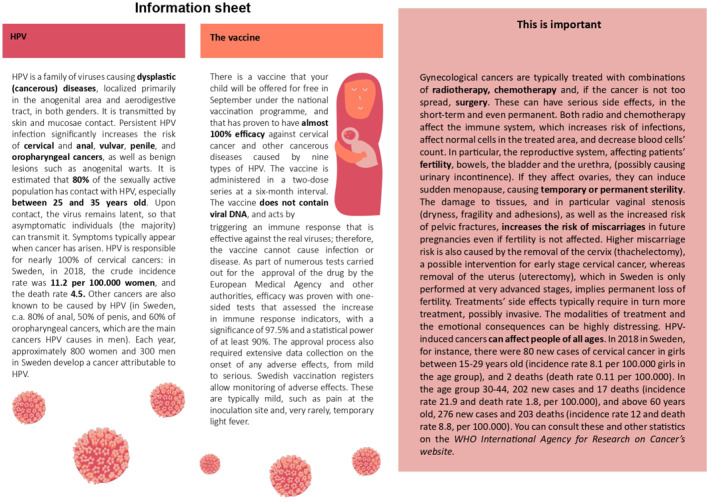
Scientifically framed leaflet (T2).

**FIGURE 3:**
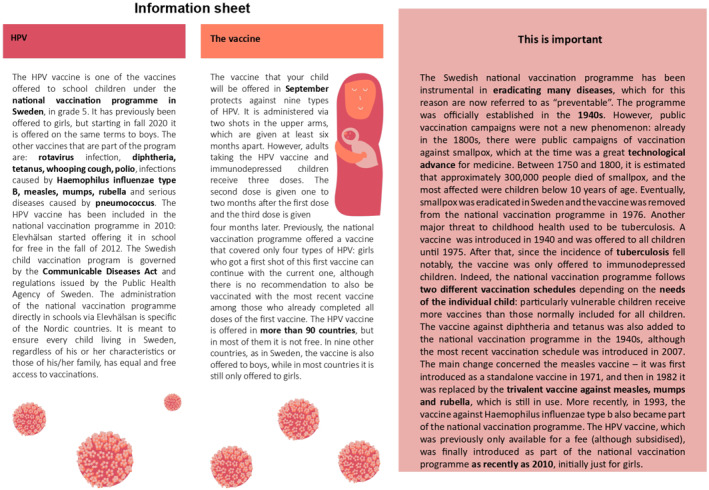
Control group leaflet (C).

Our intervention consists of a written information leaflet of approximately 650 words. It is printed on an A4‐colored paper sheet divided into three text boxes, mimicking the structure of actual leaflets from the *Swedish Public Health Agency*.^18^ The leaflets, reported in their English translation in Figures 1, 2, 3, have been compiled by the authors using information from several sources that are summarized in Supporting Information S1: Table J.22 in the Appendix. The *content is kept fixed* across the two treated arms and addresses common concerns and misconceptions in our populations of interest, which are leveraged by vaccine disinformation. For each topic that causes concern, we shift the focus away from the adverse effects of the vaccine by underlining the possibility and consequences of catching HPV‐induced cancer. In particular, the treatment leaflets report the following content (the location within the leaflets is indicated between parentheses):

Topic 1. **Reminder (central box “The vaccine”):** Reminder of the upcoming vaccination possibility;
Topic 2. **Information on HPV (left box “HPV”):** There are many types of HPV, which can cause a number of cancers, affecting both men and women of all ages. The most common HPV‐induced cancer is cervical cancer, which imposes a non‐negligible death toll on women in Sweden. It is estimated that almost every adult enters in contact with HPV in their life and often it remains asymptomatic: this makes infected people potential virus spreaders, and vulnerable to discovering cancer when it is already at an advantaged stage and treatment can be invasive;
Topic 3. **Efficacy and safety of the vaccine (central box “The vaccine”):** The HPV vaccine is almost 100% effective in preventing infection and its safety has been extensively tested as part of its approval by the European Medical Agency (EMA);
Topic 4. **Mildness and rarity of adverse effects (central box “The vaccine”):** The vaccine's adverse effect are closely monitored in Sweden, and are typically very rare and mild in nature;
Topic 5. **Cervical cancer is an actual threat for women of all ages (left box “HPV” and right box “This is important”):** Cervical cancer, and its treatment, affect women of all ages, and it can be deadly also for young women;
Topic 6. **Cancer treatment is very invasive and has very serious side effects (right box “This is important”):** The treatment of HPV‐induced can be very invasive and distressful. Cervical cancer is typically treated with combinations of surgery (if it is caught before it spreads excessively), chemo and radiotherapy. These therapies can have serious adverse effects: we pose particular emphasis on the loss of fertility (both temporary and permanent), and we also mention that they can facilitate the emergence of other infections, which can require further invasive treatment.

Qualitative studies emphasize that policy interventions targeting non‐European immigrants should address their specific concerns, which, in the specific case of HPV, also relate to the stigma associated with sexually transmissible diseases. Indeed, while cultural factors, religious in particular, increase overall vaccine hesitancy due to vaccine components of porcine origin and concerns about vaccines causing sterility (WHO Africa 2014; WHO EMRO 2014; Ahmed et al. 2018; Martinez‐Bravo and Stegmann 2021), vaccinating girls and boys as young as 12 against a sexually transmitted disease (STD) is often interpreted by parents as signaling acceptance of pre‐marital active sexual life, which entails high reputational costs in some immigrants' cultures (Wong 2009; ECDC 2017).^19^

While the content is fixed, we *randomize the framing* of the leaflet based on two treatment groups: emotional framing (T1), and scientific framing (T2). We add a third control leaflet (C) that still contains a reminder of the upcoming possibility of vaccination, plus some irrelevant text of the same length as the treatment leaflets on the history of the Swedish vaccination program. This leaflet is modeled on the basis of the actual reminder and consent form that schools distribute to parents before commencing the vaccination campaign, which we included in Supporting Information S1: Section A of the Appendix. As a result, our control leaflet (C) does report some information on the HPV vaccine, similar to the actual reminder: the notion that the HPV vaccine is part of the national vaccination program, that it previously only included girls but boys are also covered since 2020, that HPV can cause different types of cancers, that the current vaccine is administered with two doses whereas adults require a three‐dose vaccination schedule. Other information we included to match the length of treatment leaflets—such as the history of the HPV and other vaccines offered by the program, the school‐based delivery of vaccines—should be already known to parents or should have a minimal effect on uptake, relative to the treated leaflets where we cover the safety and efficacy of the vaccine, plus the negative consequences of catching HPV‐induced cancers. Nevertheless, this content might not be precisely neutral and may enhance trust in the vaccination program. As a result, our treatment effect estimates from comparing treatment and control leaflets: (i) net out the effect of receiving an extra reminder like the one typically sent by schools to parents, and instead identify only the effect of framed information on efficacy, safety of the vaccine, and consequences of catching HPV‐induced cancers; (ii) return conservative, lower‐bound estimates of the effect of framed information.

The first difference between T1 and T2 concerns the framing of topics (5) and (6)—“Cervical cancer is a threat at all ages”, and “Cancer treatment is highly invasive and has very serious side effects”. In emotional framing (T1), this information is reported in terms of testimonies of local cervical cancer survivors who describe in non‐technical but emotionally charged language their experience with cervical cancer treatment, and how it affected their fertility.^20^ In scientific framing (T2), the language is emotionally neutral and includes medical terminology: where possible, medical procedures have been mentioned using their technical name. The notion that cancer affects women of all ages is reported in terms of incidence and death rates in Sweden. The sources of this information are clearly indicated in both leaflets, and the invitation letter—sent by *Statistics Sweden* and *Karolinska Institute*, two credible sources—clearly states that all the information provided is true.^21^

The second framing difference between treatment groups concerns the inclusion of statistical concepts and jargon. Within emotional framing (T1), we only report the ratio (e.g., “8 in 10 adults”) of adults who enter into contact with HPV and the absolute number of cervical cancer cases (and deaths) in Sweden from 2018. We explicitly avoid the use of percentages, incidence and mortality rates. Both are, instead, included in the scientific framing (T2). In addition, T2 explicitly mentions three concepts from inferential statistics: one‐sided tests, statistical significance, and power of the test—relative to Phase‐3 clinical trials conducted on the HPV vaccine (Gardasil 9) by the European Medical Agency (EMA. European Medical Agency 2015).

Both individual testimonies and scientific jargon—even when it cannot be easily understood by the audience—are used by disinformation on vaccines. Individual recounts are used more often in the context of social and traditional media disinformation (Hoffman et al. 2019; Yiannakoulias et al. 2019; Kearney et al. 2019). A famous example of scientifically framed disinformation is the MMR scare, which originated by Wakefield et al. (1998)'s then‐retracted study on the supposed link between the MMR vaccine and autism, and spurred a long‐lasting pseudo‐scientific debate in the media.

The status quo in Sweden is also the reason we focused on written information, by far the most used type of campaign chosen by the *Public Health Agency of Sweden*.^22^ Compared to videos and other social media content, its diffusion and targeting can be more easily controlled by policymakers. For instance, authorities can exploit schools as informational hubs to reach parents who can hardly be reached with online campaigns. In countries that do not hold detailed administrative data, neighborhood segregation by socio‐economic status still allows targeting schools, especially in the presence of catchment areas and school‐based vaccination programs (e.g., UK, Canada, Australia).^23^ By comparing different leaflets, we investigate whether framing affects how much people engage with written information, eventually determining the effectiveness of written informational campaigns. Our leaflets are 650 words long, and the average adult reads 238 English words per minute (Brysbaert 2019): assuming a comparable speed in Swedish and other languages used in our intervention, this implies an average reading time of 2.73 min for our leaflets.

The sender of the information is an important aspect of this type of intervention, as it determines the credibility of the source and ultimately the trust of receivers. In our experiment, we mirror the same delivery method adopted by the *Public Health Agency*. The leaflet and invitation to answer surveys were sent by *Statistics Sweden*, and the invitation text (which has distinct graphical features of all *Statistics Sweden* materials) explicitly mentioned that the data collection was conducted in collaboration with researchers from the *Karolinska Institute*. The two institutional logos are clearly visible on the letter, and both institutions are generally highly regarded and well‐known, and played a significant role during the COVID‐19 pandemic, before and during our intervention. *Karolinska Institute* is the largest producer of medical research in Sweden and has an excellent reputation among the general public: for instance, it awards the Nobel Prize in Medicine and Physiology and is consistently ranked among the top medical universities worldwide. *Statistics Sweden*, the National Statistics Bureau, is formally a governmental agency and played a key role in disseminating information on the spread of COVID‐19 (Brusselaers et al. 2022). Even during the pandemic, Swedes reported a general trust of scientists similar to the rest of Western Europe, and particularly high opinion of how science benefited their country (Wellcome Trust 2021).

## 4 Evaluation Design and Outcomes

Our goal is to evaluate the effect of framed information across educational levels, and with an extra focus on immigrants. Therefore, we stratify mothers by education level and immigration background, for a total of five strata. One stratum is dedicated to all immigrant mothers from the selected origin countries (Eritrea, Somalia, Iran, Iraq, Syria, Afghanistan) regardless of their education level, though their education is observed and controlled for in our analyses. The remaining four strata are Swedish‐born mothers, categorized by their highest educational attainment. Table 1 describes the definition of strata, the number of subjects, and their allocation to treatment arms. It also reports the number of respondents to the first survey in brackets: these constitute the available sample for secondary, self‐reported outcomes.^24^ Within each stratum, randomization to treatment arms is at the individual (mother‐child) level. Children in our sample attend 611 schools located in 49 municipalities. Supporting Information S1: Section H.4 in the Appendix shows the distribution of the number of sampled children within the same school and presents a robustness check to exclude the presence of spillover effects. Note that our ethical approval prevents us from extracting data beyond our sample. Among others, this excludes the possibility of investigating spillovers on siblings or classmates.

**TABLE 1:** Stratified design and sample sizes for the primary and secondary outcomes.

Stratum	Stratum definition	*N*	C units Control	T1 units Emotional	T2 units Scientific
1. Immigrants	Selected origin countries	2548	611	961	976
		(416)	(106)	(148)	(162)
Swedish‐born mothers					
2. Educ‐level‐1	≤ 3 years high school	1627	393	616	617
	End of compulsory schooling	(353)	(94)	(138)	(121)
3. Educ‐level‐2	(3 years high school, high school degree)	1413	337	535	541
		(484)	(112)	(203)	(169)
4. Educ‐level‐3	(High school degree, Undergrad)	1009	243	385	381
		(417)	(101)	(168)	(148)
5. Educ‐level‐4	> Undegrad degree	1019	242	387	390
		(534)	(122)	(213)	(199)
Total		7616	1826	2884	2905
		(2204)	(535)	(870)	(799)

*Note:* Immigrants are mothers born in Iraq, Iran, Syria, Afghanistan, Eritrea or Somalia. Stratum 2 comprises mothers with at most 3 years of high school: This corresponds to Swedish *högstadiet* (grades 7–9), the last compulsory grades under Swedish law. Mothers in stratum 3 completed high school (*gymnasium*, grades 10–12), which is not compulsory and comprises different tracks, including vocational ones. Numbers in brackets indicate survey respondents, that is subjects for whom we can estimate results on the secondary outcome (intention to vaccinate).

Section E in the Supporting Information S1: Appendix shows balance tests of pre‐treatment covariates across treatment arms both in the full sample and restricting to survey respondents. The only meaningful difference in the full sample (>0.1 of a standard deviation) is that children assigned to emotional framing (T1) are more likely to have a parent working in healthcare relative to the control group (by 0.12 st. dev.). This difference is driven by fathers, who are not the sampling and randomization unit: there is, as expected, no significant difference in maternal medical education. After restricting to survey respondents, this difference remains present and is also found between scientific framing and the control group (0.13 st. dev.). Moreover, respondent mothers who receive emotional framing have a higher high school final grade relative to the control (by 0.13 st. dev.). These differences are taken into account by including covariates in the model. The section also shows that there is no differential attrition (i.e., probability of not responding to the first survey) by treatment status.

The timeline of the study was the following: at baseline (May 2021), our implementing partner *Statistics Sweden* identified the population of eligible mothers within each stratum and randomly selected subjects from the population using registers. The baseline information on mothers, their partners and the child was also made available from registers held by *Statistics Sweden* and the *Public Health Agency of Sweden*. Invited mothers were contacted by ordinary post at their home addresses in mid‐June 2021. The letter contained the informational leaflet, a short description of the study that did not explicitly mention the topic of vaccinations, the informed consent to take part in the first survey (with instructions to access it online after reading the leaflet), and the paper version of the survey, sent in case some mothers who preferred it to the online version.

Mothers who did not answer the survey in June received up to three reminders, until August 2021.^25^ All the materials were written in Swedish. However, the invitation letter stated (in English) that it was possible to access the leaflet and survey in English by logging in to the online version. Immigrant mothers received all printed materials in Swedish, plus a printed copy in either Arabic or Farsi, depending on their country of birth. Supporting Information S1: Section B in the Appendix shows the original invitation letter. Supporting Information S1: Table J.23 in the Appendix summarizes the content of each envelope (original + reminders) along the entire timeline. The vaccine was offered in schools between the end of September and the month of October 2021. Finally, in November, mothers who replied to the first survey received an invitation to compile an endline survey.

We did not have direct control over who replied to the survey. However, we find that on average, respondents and non‐respondents differ along maternal and not along paternal baseline characteristics, suggesting that mothers are those answering (see Supporting Information S1: Table N.31 in the Appendix). This aligns with previous literature showing that mothers are often in charge of decisions on children's health and managing doctor appointments (Case and Paxson 2001; Daly and Groes 2017). The trial was pre‐registered with this design at the AEA Registry (Dahlström and Dominici 2024) and *ClinicalTrials.gov*. Supporting Information S1: Section D in the Appendix summarizes deviations from the pre‐analysis plan.

### 4.1 Outcome Variables: Definition and Measurement

We measure vaccination uptake, our outcome, in two ways:

1. **Actual vaccination (primary outcome).** These are administrative records from population vaccination registers, measured as a binary indicator of whether the child has received the first dose of the HPV vaccine, and observed for all sampled children (N=7616);
2. **Intention to vaccinate (secondary outcome).** Subjects are instructed to answer a short survey after reading the leaflet. We ask “As of now, how likely is it that you will authorize the HPV vaccination for your child in the autumn?” and collect answers on a seven‐point Likert scale. We code a binary variable equal to one if the parent chose one between “Slightly likely”, “Likely” or “For sure”, and 0 otherwise. We observe this outcome only for survey respondents (N=2204);

The rationale for multiple measures is contributing to the understanding of how measurement impacts health economics studies about vaccinations: given the rarity of vaccination registers, it is standard practice to resort to both self‐reported intentions to vaccinate and ex‐post indicators (e.g., Alsan and Eichmeyer 2024).^26^

## 5 Estimation and Results

Within each stratum and for the whole sample of Swedish‐born mothers, we estimate the following logit model:

(1) $$\log\left(\frac{P\left(Y_{i}=1\right)}{P\left(Y_{i}=0\right)}\right)=\alpha +\tau T_{i}+X_{i}^{\prime }\beta +\eta_{m}+\varepsilon_{i}$$

The outcome $P\left(Y_{i}=1\right)$ is the probability that the child receives the vaccine according to our two measures, namely actual uptake and self‐reported intention to vaccinate.

Having a binary outcome, we prefer the logit estimator since it minimizes the estimates' variance and bias in magnitude without affecting the causal interpretation, which follows from the randomized design.^27^ $T_{i}$ is a binary treatment status indicator. Our primary interest is assessing the effectiveness of each treatment against the reminder in the control group (T1 vs. C and T2 vs. C)—the effect of framed information on top of a reminder—, which we complement with the statistical significance of the two treatments against each other (T2 vs. T1)—the effect of framing conditional on receiving information. In compliance with the pre‐registration, in Supporting Information S1: Section H.3 of the Appendix we also report the results of testing the effectiveness of any treatment (T, i.e., either T1 or T2) against the sole reminder (C).

X is a vector of pre‐treatment covariates aimed at increasing statistical power and precision, included in the pre‐registration. It includes:

- Outcome before treatment: number of MMR vaccine doses received by the child^28^;
- Child characteristics: gender, birth order (relative to the mother's children);
- Maternal characteristics: total number of children, income and net capital gains in the 12 months before sampling, age, a dummy for being married, three dummies indicating whether the highest educational attainment is focused on numerical, scientific or medical subjects, a dummy for having a research job, grade at the national high school exam (from stratum 3);
- Paternal/both parents' characteristics: a dummy for whether the father is a Swedish citizen; grade at the national high school exam; amount of government transfers received by both parents; dummies for whether any parent has a job in healthcare or research;
- For immigrant mothers in stratum 1: country of origin dummies, education level. Relative to Swedish parents, the percentage of immigrant mothers who graduated in Sweden is very low (less than 11%). For the restricted sample on secondary outcomes (only survey respondents), we also include a dummy indicating whether they compiled the survey in Swedish as a proxy of integration.

$\eta_{m}$ are municipality fixed effects (our children attend 611 schools located in 49 municipalities). We follow Abadie et al. (2023) and do not cluster standard errors, since randomization is at the individual level. To account for the sporadic missingness in registry data and preserve power, we use multiple imputation (with 5 datasets) on the pre‐treatment covariates, following Little and Rubin (2019).^29^

Results are presented in terms of average marginal effects (AME): Table 2 refers to vaccination status measured from registers, whereas Table 3 shows results for the self‐reported intention to vaccinate. For actual vaccination records, our primary outcome measure, the estimates identify an Intention‐To‐Treat (ITT) effect for the overall population from which each stratum is randomly sampled. For the intention to vaccinate (our secondary measure), the estimand is an Average Treatment Effect (ATE) relative to the subpopulation of survey respondents: this group is policy‐relevant because by answering, these individuals decided to actively engage with our materials and participate in our study. This is key considering that the envelope was mailed by *Karolinska Institute*, a well‐known health research institution, to resemble a real‐world potential implementation. While, just like the policymaker, we cannot ensure that replying implies full attentiveness, our survey data indicate that both Swedish‐born and immigrant mothers report to have read between 90% and 100% of it (which is both the median and mode answer: the full distribution is shown in Supporting Information S1: Figure N.16 in the Appendix). Section 7 will further show that responding is associated with higher engagement with our materials, and that the self‐reported percentage read is unlikely to be inflated by social desirability bias.^30^

**TABLE 2:** ITT effect of information framing on actual vaccination uptake.

Stratum	Stratum definition	Uptake in control group	T1 vs C Emotional	T2 vs C Scientific	T2 vs T1
1.	Immigrants	0.773	−0.016	−0.013	0.002
			(0.020)	(0.020)	(0.017)
Swedish‐born mothers					
2. Educ‐level‐1	≤ 3 years high school	0.786	0.037	0.057**	0.029
			(0.025)	(0.024)	(0.021)
3. Educ‐level‐2	Up to high school	0.887	−0.048**	0.004	0.041**
			(0.022)	(0.021)	(0.020)
4. Educ‐level‐3	Up to UG	0.905	−0.016	−0.021	−0.010
			(0.026)	(0.025)	(0.023)
5. Educ‐level‐4	Graduate	0.930	0.003	0.005	−0.006
			(0.020)	(0.021)	(0.018)
Total (Swedish‐born)		0.867	−0.002	0.015	0.018
			(0.120)	(0.012)	(0.010)

*Note:* Results are estimated on the entire sample of invited subjects: they can be interpreted as an ITT effect for the entire population. Immigrants are mothers born in Iraq, Iran, Syria, Afghanistan, Eritrea or Somalia. Stratum 2 comprises mothers with at most 3 years of high school: this corresponds to Swedish *högstadiet* (grades 7–9), the last compulsory grades under Swedish law. Mothers in stratum 3 completed high school (*gymnasium*, grades 10–12), which is not compulsory and comprises different tracks, including vocational ones. The total effect is restricted to Swedish‐born mothers due to different sets of covariates and the regression includes stratum dummies.

*p < 0.1.

** *p* < 0.05.

***p < 0.01.

**TABLE 3:** ATE of information framing on the intention to vaccinate.

Stratum	Stratum definition	Uptake in control group	T1 vs C Emotional	T2 vs C Scientific	T2 vs T1
1.	Immigrants	0.830	−0.039	−0.003	0.003
			(0.053)	(0.047)	(0.048)
Swedish‐born mothers					
2. Educ‐level‐1	≤ 3 years high school	0.862	0.002	0.115**	0.025
			(0.045)	(0.046)	(0.036)
3. Educ‐level‐2	Up to high school	0.929	−0.021	0.022	0.029
			(0.033)	(0.032)	(0.028)
4. Educ‐level‐3	Up to UG	0.931	0.036	−0.010	−0.042
			(0.036)	(0.035)	(0.032)
5. Educ‐level‐4	Graduate	0.967	−0.003	−0.018	−0.008
			(0.021)	(0.025)	(0.021)
Total (Swedish born)		0.925	−0.000	−0.001	−0.001
			(0.016)	(0.016)	(0.014)

*Note:* The outcome variable is the self‐reported intention to vaccinate (binary indicator). Results are estimated on the subsample of survey respondents for whom the outcome is observed: They can be interpreted as an ATE effect for this subpopulation. Immigrants are mothers born in Iraq, Iran, Syria, Afghanistan, Eritrea or Somalia. Stratum 2 comprises mothers with at most 3 years of high school: This corresponds to Swedish *högstadiet* (grades 7–9), the last compulsory grades under Swedish law. Mothers in stratum 3 completed high school (*gymnasium*, grades 10–12), which is not compulsory and comprises different tracks, including vocational ones. The total effect is restricted to Swedish‐born mothers due to different sets of covariates and the regression includes stratum dummies.

*p < 0.1.

** *p* < 0.05.

***p < 0.01.

Consistent with our hypothesis, we find a null result for the overall population of Swedish‐born mothers, which hides heterogeneous effects across education backgrounds. These could have important policy implications when designing informational campaigns. Table 2 reports that for mothers with only compulsory schooling in stratum 2, only scientifically framed information (T2) has a significant positive effect, leading to a 5.7 percentage points increase in uptake (equivalent to 7.25%). For mothers who continued their studies and obtained a high school degree in stratum 3, T2 is ineffective $\left({\hat{\tau }}_{\mathrm{AME}}=0.004\right)$. In contrast, emotionally framed information (T1) has a negative significant ITT effect, leading to a 4.8 percentage points decrease in uptake (a 5.41% decrease). Emotionally framed information never appears to have a statistically significant positive effect, and (T2 vs. T1) tests confirm that T1 never overperforms with respect to T2 by a significant or policy‐relevant amount, despite not being significant in stratum 2 due to lower power (our minimum detectable size exceeds the 0.029 estimate by roughly 2 percentage points). Similarly, (T vs. C) tests in Supporting Information S1: Section H.3 of the Appendix are only significant in stratum 2, driven by the positive effects of scientific framing (T2). Our significant results are robust to the Benjamini‐Hochberg (BH) adjustment of *p*‐values for multiple hypotheses that takes into account the number of strata and comparisons performed (5×3=15), although robustness depends on the procedure adopted.^31^

Table 3 reveals that measuring the outcome as the self‐reported intention to vaccinate rather than actual vaccinations makes a difference. Comparing these uptake measures for the control group reveals that at baseline, mothers from all strata overstate their intention to vaccinate by at least 3 percentage points. The mismatch between intentions and actual vaccination choice is larger among immigrants and lowly educated mothers.

Yet, the positive effect of scientifically framed information (T2) in stratum two is also found on the intention to vaccinate. The magnitude of this estimate, however, is not directly comparable to the one in Table 2, because it refers to a different population. To obtain comparable estimates with the two outcomes, we replicate the analysis for both measurements after restricting the sample to survey respondents: the tables are reported in Section 7. Conditional on responding, scientific framing increases objective uptake by 16.1 percentage points, whereas with the subjective intention to vaccinate, the corresponding magnitude is 11.5. This reflects that while mothers overstate willingness to vaccinate in the control group, they do not after receiving the scientifically framed leaflet in stratum 2. Further descriptive statistics that compare intention to vaccinate (on a seven‐point Likert scale) and actual vaccination by stratum are presented in Supporting Information S1: Section O in the Appendix.

In the Appendix, we also show that these results are robust to several modifications of the empirical strategy. First, Supporting Information S1: Section H.2 shows that estimates are robust to excluding covariates, with minor differences due to imbalances in paternal characteristics. Second, since part of the related literature provides Linear Probability Model (LPM) estimates, in Supporting Information S1: Section H.1 we re‐estimate Equation (1) by OLS/LPM for comparability. We obtain qualitatively comparable results, although one significant estimate is downward biased when estimated by OLS/LPM. The OLS/LPM also allows us to estimate the results for willingness to vaccinate measured on the full Likert scale (1–7), which confirm the findings from the binary variable and Logit estimator.

Third, we add the following “structural” linear specification:

(2) $$Y_{i}=\alpha +\tau_{1}T1_{i}+\tau_{2}T2_{i}+X_{i}^{\prime }\beta +\eta_{m}+\varepsilon_{i}$$

The structural interpretation of Equation (2) follows from the fact that by design, T1 and T2 are never simultaneously equal to 1. The results are quantitatively comparable.^32^ Finally, in Supporting Information S1: Section I, we provide and discuss qualitative evidence that COVID‐19 impacted our study, making younger mothers less responsive to our treatments.

## 6 Heterogeneity Analysis: Causal Forest

The high variance of our estimates, even with a large sample, suggests the effects might be characterized by a large degree of heterogeneity. Moreover, understanding which baseline characteristics moderate the effect of framed information is key to planning targeted information campaigns. To investigate heterogeneity while exploiting the high number of pre‐treatment covariates in our data, we resort to causal forests (Athey and Imbens 2016). The method builds upon supervised machine learning, and in particular on CART models, to estimate conditional treatment effects. In our case, we estimate the Conditional Intention To Treat effect (CITT), as we focus on actual uptake, our primary outcome, as the dependent variable.

The estimand is defined as $E\left[Y_{1i}-Y_{0i}|X_{i}=x\right]$, where $Y_{1i}$ and $Y_{0i}$ are potential outcomes under treatment and control, respectively, and $X_{i}$ are observable characteristics of individual i. For estimation, we compare T1 and T2 individually against C. Following Athey and Wager (2019), we adopt an “honest approach”: we use half the sample to grow a forest with 10,000 trees, where each tree's leaf can contain no less than five observations, and the other half of the sample for the actual estimation. We estimate causal forests in the entire Swedish‐born sample, letting the data speak about the importance of education as a driver of heterogeneity. Immigrants are not included because of the different set of covariates. In Supporting Information S1: Section Q of the Appendix, we first show that causal forest estimates are consistent with the main results in terms of how causal effect distributions change across different educational strata, then we show their robustness if estimated within strata rather than in the whole Swedish‐born sample—including, in this case, also immigrant mothers.

**FIGURE 4:**
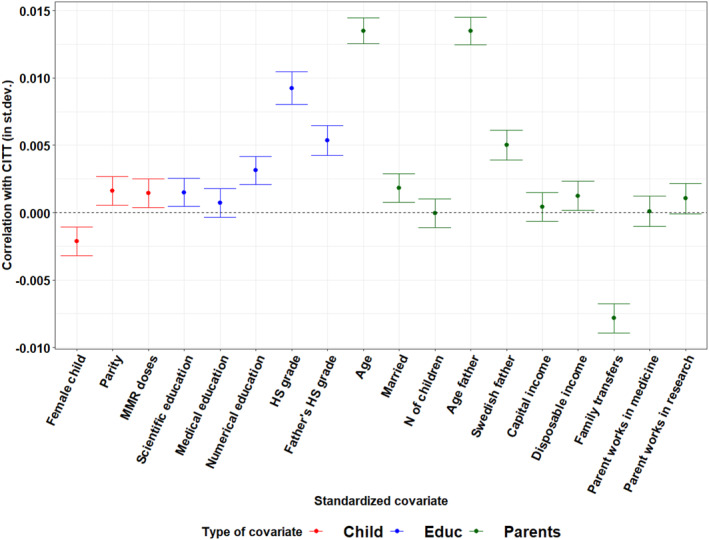
CITT estimates by baseline covariates: Emotional framing (T1). The figure shows the correlation—estimate and 95% C.I.—between individual CITT estimates from causal forests and baseline covariates, estimated by OLS. Covariates are standardized (i.e., correlations are expressed in standard deviations).

**FIGURE 5:**
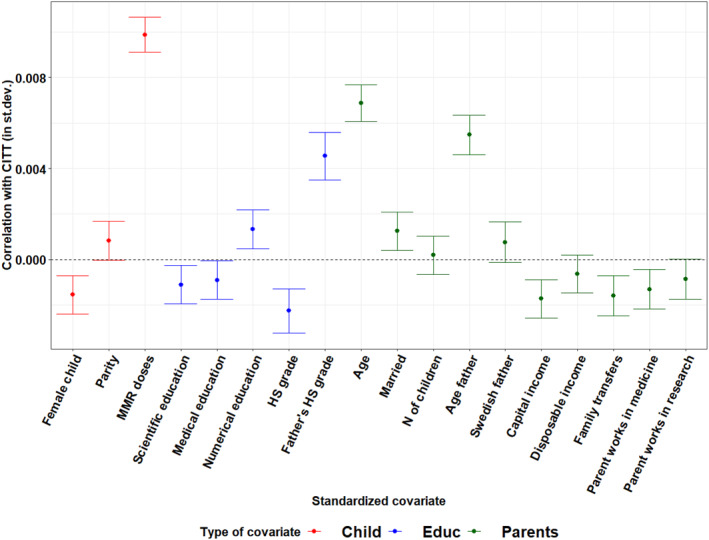
CITT estimates by baseline covariates: Scientific framing (T2). The figure shows the correlation—estimate and 95% C.I.—between individual CITT estimates from causal forests and baseline covariates, estimated by OLS. Covariates are standardized (i.e., correlations are expressed in standard deviations).

The results are summarized in Figures 4 and 5, which show the correlation between CITT estimates and baseline covariates, obtained by OLS regression. Covariates are standardized so that coefficients can be interpreted in standard deviations and easily compared. We make several observations. Overall, the effects of emotional framing (T1) are more volatile, as several covariates are associated with larger changes in CITT. In terms of policy recommendations, this implies that emotional framing is more difficult to implement, requires more careful targeting, and produces more unpredictable results, depending on how receivers' characteristics are combined. Importantly, while there is no economically significant association with overall income after including education, emotional framing has particularly negative effects among disadvantaged families, who are likely to be in most need of information: one standard deviation increase in the amount of government transfers is associated with a decrease in the effect of almost 1 percentage point.

With the exception of government transfers, treatment effects are more strongly associated with parental education than income. In particular, parental final high school grades show large associations with the effect of both emotional and scientific framing (between half and 1 percentage point for a standard deviation change in grades). While the association is always positive for paternal grades, it becomes negative (but small) for maternal grades exposed to scientific framing. Causal forests estimated within strata (reported in Supporting Information S1: Section Q.2 of the Appendix) reveal that both the sign and magnitude of this association vary across strata and that the negative association is driven mainly by highly educated mothers, remarking again on the importance of considering educational background in assessing the effect of framed informational campaigns. For immigrant mothers, education level and whether they attained a formal degree in Sweden are also the main moderators, alongside country of origin dummies and years since immigration, that reveal substantial heterogeneity across immigrant communities and the level of integration. Interestingly, the negative education gradient is only confirmed for emotional framing (T1), possibly reflecting difficulties in understanding scientifically framed content; yet, large differences driven by country of origin indicate that more research on specific targeting techniques is needed to address suboptimal coverage among immigrant communities.

Parental age is an important moderator of framed information effect, as parents react better to both the emotional and scientific treatments: since children's age is fixed, this likely reflects that becoming parents at older ages implies greater attention to children's outcomes, which is unsurprising. Another possibility is that younger mothers changed their vaccination attitudes more strongly following the COVID‐19 pandemic (Eichengreen et al. 2021). We find some suggestive evidence in this sense in Supporting Information S1: Section I in the Appendix. On the other hand, the number of MMR doses received by the child before treatment plays a different moderating role depending on the treatment, fostering the absorption of scientifically framed information more than the emotionally framed. In the estimation of causal forests used for this heterogeneity analysis, MMR doses are the only proxy of attitudes toward science and vaccines we can include, since all other measures are only observed for survey respondents. In the next section, we investigate attitudes toward vaccines and health authorities more thoroughly as a mechanism of engagement with our framed information treatment, hence of its absorption.

## 7 Mechanisms

In this section, we explore two sets of mechanisms to rationalize our main results. First, we will show that the positive effect of scientifically framed information (T2) and the negative effect of emotionally framed information (T1) are experienced by different types of parents. The positive effects of T2 are only found in parents who are less hesitant toward vaccines at baseline and are more engaged with our leaflets. Second, we show that both the positive and negative effects are driven by parents who had little or no previous knowledge of HPV, precisely those that should be targeted by informational campaigns. Taken together, these results show that framing makes a difference: nevertheless, emotional framing should be avoided for its negative effect on the less engaged and more hesitant parents and the lower efficacy on the more engaged.

### 7.1 Pre‐Treatment Vaccine Hesitancy and Engagement With the Leaflets

In our main analysis, the intention to vaccinate results only mirror the positive effect of scientifically framed information (T2) in stratum 2. Yet, self‐reported intention to vaccinate is only measured among survey respondents. Responding, therefore, probably proxies some determinants of mothers' reaction to information framing. To better understand who drives the positive reaction to scientific framing and the negative reaction to emotional framing, we re‐estimate our main ITT analysis from Equation (1) separately for subjects who never replied to our survey and subjects who replied at least to the first one. As we will discuss below, whether mothers reply proxies both their reluctance to be a target of an informational campaign—similar to J. Hirani (2021)—, and how much they engage and absorb the information in our leaflets. In turn, this provides empirical evidence to support dual‐process theories as a mechanism behind the effects of scientific and emotional framing (Evans 2008; Evans and Frankish 2009; Taute et al. 2011; J. Hirani 2021).^33^

In this section, we report the main findings, although the full tables and graphs are reported in Supporting Information S1: Section N in the Appendix. Consistent with the evidence from the intention to vaccinate, the positive effect of scientifically framed information (T2) in stratum 2 is only significant among respondents, with a higher magnitude compared to the overall sample (16 percentage points instead of 6). Among non‐respondents, the estimate is 4.4 and not statistically significant. The confidence intervals overlap, also due to the reduced sample size—and precision—in performing these heterogeneity analyses. In contrast, the difference in magnitudes is large: we interpret this as respondents driving the overall positive estimates in the full sample. Similarly, at the 90% significance level, the negative effect of emotionally framed information (T1) in stratum 3 is mainly driven by non‐respondents.

Distinguishing between respondents and non‐respondents also sheds light on the dynamics in stratum 5 that emerged from causal forest estimates. Indeed, highly educated mothers react the opposite way of lowly educated ones in stratum 2: emotionally framed information (T1) raises uptake among non‐respondents (by 6.5 percentage points). In this case, the 90% confidence intervals do not overlap.

To further investigate the mechanisms behind these differences, we then proceed to characterize respondents and non‐respondents. We find that respondents are slightly less vaccine‐hesitant, less expert in health or science, and have a higher socioeconomic status. In particular, respondents have a higher uptake of the second dose of the MMR vaccine. To clarify whether different MMR uptake proxies vaccine hesitancy and attitudes toward informational campaigns like ours, we rely on the comparison between respondents to the first survey only (R) and respondents to both surveys (RR), in order to look at differences in answers to survey questions that measure hesitancy more clearly. This would require comparable self‐selection mechanisms into responding twice instead of once or once rather than never. While we cannot test this formally, we provide quantitative support by highlighting that the demographic, educational, and occupational characteristics that change significantly between never‐respondents and respondents also change with the same sign when comparing R and RR, although the difference, in this case, is not always statistically significant.^34^ The full tables are shown in Supporting Information S1: Section N in the Appendix. Table 4 compares some crucial indicators of vaccine hesitancy and engagement with our leaflets between R and RR.

**TABLE 4:** First survey answers by treatment and number of surveys answered (N=2204).

First survey answers	Control (C)			Emotional framing (T1)			Scientific framing (T2)		
	ASD	Replied once	Replied twice	ASD	Replied once	Replied twice	ASD	Replied once	Replied twice
Believes vaccines cause the disease	−0.217**	1.854	1.532	−0.202***	1.867	1.567	−0.158**	1.859	1.616
Believes vaccines weaken the immune system	−0.179**	1.747	1.504	−0.17***	1.859	1.604	−0.165**	1.822	1.580
Trusts health authorities	0.236***	4.237	4.540	0.116**	4.353	4.500	0.123**	4.333	4.494
% of leaflet read	0.040	7.705	7.878	0.161**	7.679	8.358	0.177**	7.634	8.371
Distraction question	0.15**	0.948	0.986	0.059	0.936	0.955	0.078	0.935	0.959
Heard of HPV before the study	0.107	0.824	0.878	0.088*	0.850	0.892	0.163**	0.828	0.906

*Note:* Significance is assessed with two‐tailed tests for differences in means with different variances. The table shows, separately for each treatment status, the difference in first survey answers between subjects who only replied to the first survey, and subjects who replied to both the first and the second survey. The Average Standardized Difference (ASD), also known as Cohen's D, is computed as $ASD\left(X\right)=\frac{{\bar{X}}_{RR}-{\bar{X}}_{R}}{\sqrt{{\mathrm{Var}}_{RR}\left(X\right)+{\mathrm{Var}}_{R}\left(X\right)}}$ where X is the variable, RR denotes respondents to both surveys and R denotes respondents to the first survey only. In other words, an ASD of 0.10 means that the difference in means between the two groups equals 0.1 of the pooled standard deviation. The distraction question asked subjects to select the first option from a list of two options. The self‐reported % of the leaflet read is measured on a 1‐10 scale where 1 is “between 0% and 10%” and 10 is “between 90% and 100%”. Trust in health authorities and beliefs about vaccines are measured with a one to five Likert scale. All other variables are dummies.

* *p* < 0.1.

** *p* < 0.05.

*** *p* < 0.01.

The comparison between R and RR reveals that those who answer twice (RR) are significantly more likely to have correct beliefs about vaccines and have a higher uptake of the MMR vaccine at baseline. Moreover, the beliefs are more correct for RR in all treatment statuses, suggesting that these answers reflect baseline beliefs and are not affected by the information we provide with both our treatments, T1 and T2. In Supporting Information S1: Section K in the Appendix we test this formally: we find that among those who replied to at least the first survey, misconceptions around vaccines are largely unaffected by our treatments. Moreover, in all treatment groups, answering more surveys is associated with higher trust in health authorities. This confirms J. Hirani (2021)'s finding that people who do not vaccinate following a written reminder are reluctant. These findings are also interesting in light of the opposite reactions of stratum 3 (high school education, where non‐respondents *decrease* uptake following emotional framing), and stratum 5 (postgraduate education, where non‐respondents *increase* uptake following emotional framing). Non‐respondents are more vaccine‐hesitant, but their reaction to framed information changes by educational background. We cannot fully characterize their habitual sources of information or levels of trust in institutions because we cannot observe survey answers. Yet, two additional hypotheses arising from this evidence that could interest future research are (i) whether exposure to framed disinformation reduces the credibility of subsequent information with the same framing, and (ii) whether the identity of senders interacts with framing in systematically different ways by educational background.

Nevertheless, by comparing the results across treatment groups, we find evidence that, once framed information is delivered on top of a reminder (i.e., in T1 and T2), engagement with the leaflets acts as a mechanism. Indeed, RR subjects reported having read a significantly higher percentage of our leaflet compared to R only in our treatment groups and not in our control group, which also shows that the results are unlikely to be affected by social desirability bias. Finally, previous knowledge of HPV is only significantly different for R and RR in the treatment groups. In the next subsection, we will show evidence that engagement is associated with subjects' baseline knowledge of HPV and their exposure to previous informational campaigns.

### 7.2 Previous Knowledge of HPV

**FIGURE 6:**
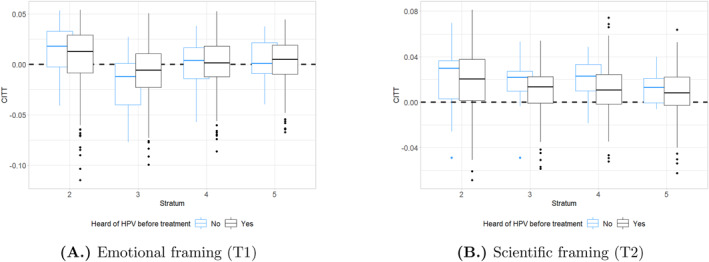
Effect of framing (CITT) by pre‐treatment exposure to HPV information. The figure shows, separately for the two treatments and strata, boxplots of individual causal effects (CITT) by pre‐treatment knowledge of HPV. The sample includes only survey respondents, for whom pre‐treatment knowledge of HPV is observed. Stratum one is not included because the CITT is estimated on the sample of Swedish‐born mothers. Results are comparable using causal forests estimated within strata (including stratum 1, where 0 always falls within the interquartile range).

Pre‐treatment exposure to HPV information is associated not only with higher engagement with our leaflets but also with weaker treatment effects. Figure 6 shows CITT estimates from the causal forest by baseline knowledge of HPV. The positive effects of scientifically framed information (T2) in stratum 2 and the negative effect of emotionally framed information (T1) in stratum 3 are less dispersed for mothers who had not heard of HPV before our intervention. In line with previous findings that out of many informational campaigns, only the first will have lasting impacts (e.g., in the case of COVID‐19 vaccinations, see Bartoš et al. 2022), we interpret this as vaccine information having diminishing returns. In other words, the more information a parent has already been exposed to, the less effective new campaigns will be in increasing their uptake.

In our data, two pieces of evidence support this hypothesis. First, boys' mothers—who had less chance of being targeted by informational campaigns since boys were only included in 2020 in the HPV vaccination program—respond more to our treatments. This is suggested by causal forests (Figures 4 and 5), whereas in Supporting Information S1: Section P of the Appendix we show that boys' mothers drive the significant effects in strata 2 and 3 and find formal evidence of differences in the pre‐treatment exposure to HPV information by the child's gender. Second, mothers who were not previously exposed to HPV information tend to have more incorrect beliefs about vaccines: descriptive evidence is shown and discussed in Supporting Information S1: Section K in the Appendix. We remain agnostic about the behavioral mechanisms that we cannot measure, and that could drive diminishing returns, such as anchoring or belief perseverance.

This complements our heterogeneity analysis results, which showed a higher treatment effect for those segments of the population that are less vaccinated, on average. In other words, our intervention is effective where information is needed. From a policy perspective, our results underline that people's attention to information is limited: ineffective or counterproductive informational campaigns are not just an economically inefficient policy intervention; they can also backfire by reducing the effectiveness of future, more impactful campaigns. This leads to two policy recommendations: sticking to scientifically framed information (since emotional framing can be counterproductive) and avoiding campaigns that are not informative.

## 8 Discussion and Conclusions

In this paper, we investigate how emotionally and scientifically‐framed information interacts with recipients' educational and cultural backgrounds in raising the uptake of the HPV vaccine: while it is the main preventive tool against a large number of cancers, this vaccine is still the target of intensive disinformation campaigns. We did so after a pandemic that has radicalized pre‐existing attitudes toward vaccinations and has increased the policy relevance of contrasting vaccine hesitancy. During the summer of 2021, we sent a written leaflet to mothers living in Stockholm County, Sweden, whose children were due to receive the HPV vaccine in the autumn. The leaflets were written following the example of actual campaigns used by Swedish government authorities, which are more suitable to target under‐vaccinated populations directly in schools relative to other digital media. To study the interaction of the information with recipients' education, our pre‐registered design stratified mothers into four strata defined by education level and one stratum dedicated only to mothers born in the largest non‐European origin countries of immigrants. We observe rich administrative information on HPV uptake (our primary outcome) and parental baseline characteristics, and we ask mothers about their intention to vaccinate and their previous exposure to HPV information in a short survey.

Our experimental intervention varies the framing of leaflets, mirroring techniques that are also employed by vaccine disinformation: emotional framing (T1) relies on emotionally charged anecdotes from cervical cancer survivors, whereas scientific framing (T2) uses medical and statistical jargon that is not immediately understandable for all laypeople. To estimate the effect of framed information disentangled from the effect of receiving one extra reminder, our control group also received a leaflet of the same length containing the reminder plus some content that is unrelated to HPV, about the history of vaccines in Sweden: since we cannot exclude this content also impacts trust in vaccines, our treatment effect estimates are a lower bound with respect to a comparison with a short, simpler reminder. Content‐wise, both scientific and emotional leaflets shift the focus away from the vaccine's adverse effects and toward the dire consequences of catching HPV‐induced cancers.

In line with our expectations, framed information has a null effect on the overall population that hides policy‐relevant heterogeneity by maternal education. Mothers with compulsory education (less than high school) increased uptake if exposed to scientifically framed information, an effect driven by those who responded to our survey, who engaged more with reading the leaflet, and had more favorable vaccine attitudes at baseline. Conversely, mothers with a high school degree showed decreased uptake following emotionally framed information, driven by more vaccine‐skeptic mothers who did not respond to our survey. Heterogeneity by survey participation and causal forests also revealed positive reactions to emotionally framed information by non‐responding mothers with postgraduate education, who essentially reacted the opposite way from high school graduates. These differences depend on education rather than subsequent income characteristics, and underline that targeting framed informational campaigns must consider complex heterogeneity patterns. While framed information can improve uptake beyond the effects of simple reminders, there is no one‐size‐fits‐all solution. We also argued that the effects were driven by mothers with little previous knowledge of HPV who most need informational campaigns, and that the effectiveness of framed information (both positive and negative) is characterized by diminishing returns: later campaigns will have increasingly smaller effects.

In terms of methodology, we show that results can be affected by how vaccination outcomes are measured. Indeed, in all strata—and especially among immigrants and lowly educated mothers—the self‐reported intention to vaccinate in the control group overstates actual vaccination uptake, but not in treated groups, leading to a lower estimated effect of treatment. This is particularly important since access to objective vaccination records that do not suffer from attrition is often impossible or strongly limited. In those cases, results based on self‐reported intentions are likely lower‐bound estimates of the effects on actual uptakes.

Our results should be interpreted with some limitations in mind, which could constitute interesting starting points for future research. First, while we highlight the important differences across educational backgrounds, our data do not allow us to pinpoint the mechanisms that drive opposite reactions to emotionally framed information between lowly and highly educated non‐respondents. Similarly, although the within‐strata analysis was pre‐registered, the sample was randomly selected from the full population, and attrition was absent for the primary outcome, with results consistent across multiple robustness checks, it is important to note that the robustness of our findings to multiple hypothesis testing may vary depending on the testing procedure employed. We also conducted our experiment as COVID‐19 vaccines became available and provide suggestive evidence that the saliency of the pandemic might have impacted our results: our results are relevant to the unfortunate but possible scenario of future pandemics, yet new evidence could focus on the post‐pandemic period. Finally, this type of randomized trials adopt specific definitions of emotional and scientific framing by construction and must, by European law, inform subjects that they are part of a study. While Sweden has a high level of trust in scientists and should not be particularly affected by this, we cannot tell if results would change in a counterfactual experiment with unaware subjects.

On the positive side, two limitations actually imply that our estimates are conservative, suggesting possibly higher magnitudes in actual applications of framed infomation campaigns. First, our control group received an extra reminder by a recognized health authority, which also included some basic information to mimic real campaigns in our context: as a result, one could expect larger effects from running a framed campaign vis‐à‐vis not receiving anything. Second, our focus on the Stockholm County meant to strengthen internal validity due to the over‐representation of immigrants and highly educated mothers, coupled with significant effects in lowly educated strata, suggests larger effects in more peripheral areas where low education is more common.

Still, our study mimicked actual informational campaigns in our setting along several dimensions and generated four readily implementable policy recommendations. First, informational campaigns should use scientific framing and target parents with a lower educational background, since low education stands out as the main driver of the effectiveness of framed information. Indeed, the positive effects of emotional framing are modest, and concentrated among a small group of less informed parents with postgraduate education, for whom, however, coverage is already above the policy target before our intervention; on the other hand, we find counterproductive effects on mothers with high school education, who represent an under‐vaccinated group. Based on this evidence, our second recommendation is to avoid emotional framing: its effects are more volatile and less predictable, and never significantly outperform scientific framing. Our third recommendation is to design fewer targeted campaigns rather than more numerous and generally‐oriented campaigns. Indeed, the parents who react more strongly to scientific framing are those who have little previous exposure to information and lower vaccination uptakes: this suggests that recipients' responsiveness will decrease over time when exposed to campaigns focused on the same content; at the same time, it shows the our scientific framing intervention effects are found precisely where they were most needed from a policy perspective. Finally, engagement with leaflets matters: written leaflets allow for targeting hard‐to‐reach groups, but they should be delivered in contexts that combine the possibility of targeting socioeconomic backgrounds and monitoring information absorption, such as schools and local general practitioners.

## Ethics Statement

The trial has ethics approval from the Swedish Ethical Review Authority (*Etikprövningsmyndigheten*, number 2020‐01368), and was pre‐registered as AEARCTR‐0007668 (Dahlström and Dominici 2024).

## Conflicts of Interest

The authors declare no conflicts of interest.

## Supporting information


**Supporting Information S1:** hec70036‐sup‐0001‐suppl‐data.pdf.

## Data Availability

The data are not publicly available due to privacy or ethical restrictions.

## Endnotes

^1^
Vaccine hesitancy was listed among the World Health Organization's *Ten threats to global health*.

^2^
For instance, see Salmon et al. (2023); Jarrett et al. (2015); v. Dubé et al. (2015); Thomson et al. (2016).

^3^
As of January 2019, according to the World Health Organization fact sheet.

^4^
Fake news even claim that vaccinated people might affect women's fertility by standing close to them (e.g., https://www.nbcnews.com/tech/internet/viral‐vaccine‐infertility‐misinformation‐finds‐home‐social‐media‐n1267310), and have now spread to also consider men (e.g., https://eu.usatoday.com/story/news/health/2021/06/21/pfizer‐moderna‐covid‐vaccines‐do‐not‐decrease‐sperm‐count‐study‐says/7770928002/).

^5^
Kids who are absent on the day of vaccination can still be vaccinated by the school nurse when they come back to school: school absences do not constitute a problem in our setting.

^6^
Supporting Information S1: Section A in the Appendix provides extra information on the Swedish vaccination program and uptake measures.

^7^
The governmental leaflet can be found at this link.

^8^
Indeed, J. Hirani (2021) and J. C. Hirani and Wüst (2024) find that reminder letters are effective for parents who lag behind with their children's vaccinations in Denmark.

^9^
In particular, J. C. Hirani and Wüst (2024) shows that a 2019 reform that introduced reminders of upcoming vaccination opportunities increased uptake by 9.4% for the HPV vaccine and 7.2% for the MMR vaccine. Despite the differences with the Swedish Vaccination Program, most notably the fact the in Denmark the HPV vaccine is inoculated by the general practitioner instead of school nurses, adding the extra non‐monetary cost for parents to set up an appointment and choose a timing of vaccination, Denmark shares the fundamental feature of offering the HPV vaccine for free: as shown by Humlum et al. (2024), this has a huge positive impact on coverage (53.2 percentage points for girls and 36.0 percentage points for boys). Turning to the U.S. context and to the flu vaccine, which is not free, megastudies on several types of reminders in the United States found on average a 5% increase for vaccines administered during doctor appointments (Milkman et al. 2021) and 6.8% (2 percentage points) for vaccines administered at pharmacies (Milkman et al. 2022).

^10^
For instance, besides the Swedish survivor association we relied on to compile our emotional leaflets, emotional survivor testimonies are used by U.S. state and federal health authorities (e.g., Minnesota Department of Health 2024; Centers for Disease Control and Prevention 2024), by WHO agencies (Pan American Health Organization 2024), by global advocacy groups, collecting testimonies from all continents (Cervivor and Inc mar. 2025), and by NGOs (e.g., for two authoritative examples from the UK and Australia, see Cancer Research UK 2024; Cancer Council Victoria 2024).

^11^
Relative to COVID‐19, in the US, Rabb et al. (2022) and Dai et al. (2021) test several nudges in short messages and find mixed evidence, highlighting the role of recipients' levels of hesitancy and its interaction with timing and mode of message delivery. Galasso et al. (2022) test a series of nudges in 9 countries to identify which informational contents are most effective in raising intentions to vaccinate. In Sweden, Campos‐Mercade et al. (2021) find that nudges were mildly effective but not as much as monetary incentives. Abstracting from COVID‐19, Banerjee et al. (2021) also test several nudges on the uptake of a childhood vaccine—measles –, in the context of India. Randomized informational interventions are also present in the field of epidemiology (Tiro et al. 2015; Nyhan et al. 2014; Nyhan and Reifler 2015; Horne et al. 2015). In particular, Cox et al. (2010) investigated the effect of framing HPV vaccine information on 471 mothers of girls between ages 11 and 16. Treatments vary in whether statistical information is presented at all and, if so, whether in a graphical or written form. The authors do not focus on mothers' education, and their U.S.‐based experiment may have income‐related confounding effects.

^12^
Nudges—applied to contents or framing alone—are receiving attention in several fields of economics. Closer to our work, eliciting emotional responses increases charitable donations (Zarghamee et al. 2017). Two more recent examples are Delfino (2021), in labor economics, which uses pictures and information about the degree of competitiveness to increase male applications to female‐dominated jobs, while in finance Barboni et al. (2022) test several written nudges to encourage repayment from debt holders.

^13^
In Europe, there are 33,000 annual cases and 15,000 deaths caused by cervical cancer (ECDC. European Centre for Disease prevention and Control 2018).

^14^
Bruni et al. (2023) report the age‐standardized incidence per 100.000 inhabitants of all HPV‐induced cancers in Sweden compared to the world figure. It has a higher incidence for the following cancers: anal (0.61, vs. 0.49), vulvar (1.34 vs. 0.85), penile (0.91 vs. 0.80), oropharyngeal (3.85 vs. 1.79 for men, 1.31 vs. 0.40 for women). Instead, cervical and vaginal cancer incidences are close to the global incidence rate (respectively 10.4 vs. 13.3 and 0.26 vs. 0.36).

^15^
We focus on mothers because they are more often responsible for decisions on children's health and for managing doctor appointments (Case and Paxson 2001; Daly and Groes 2017). We discuss this point again in Section 4 and in the Supporting Information S1: Appendix.

^16^
Note that our ethical approval does not allow us to extract data outside of this sample: this prevents us from observing mothers who did not receive any leaflet during our intervention, or to assess spillovers outside of sampled individuals.

^17^
This strengthens internal validity at the potential detriment of external validity: in light of our results and on scenarios on a distribution of mothers' education closer to the national average (Supporting Information S1: Section S of the Appendix), we hypothesize that our study might actually underestimate the positive effects of a scientifically framed campaign and the negative effects of emotionally framed information aimed at the less educated strata of the population.

^18^
The original governmental leaflet can be found at this link: https://www.folkhalsomyndigheten.se/contentassets/6401e69460ef411faba95ac12b225662/hpv‐fakta‐eng‐20052‐3.pdf.

^19^
This might imply that reminding parents HPV is sexually transmitted might also decrease uptake: in the absence of previous quantitative literature, we had no prior on the effect for immigrant mothers. Similar moral hazard phenomena, known as the Peltzman effect (Peltzman 1975) have been documented following increased access to condoms, emergency contraception, and HIV tests (e.g., Girma and Paton 2011; Durrance 2013; Buckles and Hungerman 2018; Philipson and Posner 1995). Using data from our endline survey, we can verify that immigrant mothers in our sample attribute higher importance to premarital virginity compared to Swedish‐born mothers. However, the small and positively selected sample in terms of vaccine attitudes (N=96) is such that we cannot detect a difference in the perceived role played by the HPV in incentivizing premarital sex.

^20^
These have been extracted with the consent of their authors from the stories published by *Nätverket mot gynekologisk cancer*, a Swedish association of gynecological cancer patients, survivors and their relatives. It can be inspected online at: https://gyncancer.se/category/dinhistoria/.

^21^
Note also that the experiment was carried out before the spread of generative AI.

^22^
All educational materials on vaccinations aimed at both parents and health professionals can be found on the Swedish Public Health Agency's website: https://www.folkhalsomyndigheten.se/smittskydd‐beredskap/vaccinationer/kommunicera‐om‐vaccinationer/.

^23^
While a full cost‐benefit analysis goes beyond the scope of this study, in Supporting Information S1: Section S we provide estimates in light of our results.

^24^
The differences between survey respondents and non‐respondents, as well as the characteristics of respondents to the second survey, are further discussed in our mechanisms' analysis in Section 7 and in Supporting Information S1: Section R in the Appendix.

^25^
The choice of mid‐June for the first contact is meant to maximize the number of mothers found at home before vacations. The most important summer holiday in Sweden, *midsommar*, marks the beginning of summer: from then until August, there is no customary period when most of the population goes on vacation. Scattering reminders over the entire period maximized the probability that all sampled mothers receive an invitation while at home.

^26^
In Supporting Information S1: Section R of the Appendix, we complement with a third measure collected in the second survey: after vaccination took place, mothers who replied to the first survey are contacted again to answer a second survey (in November). We ask them “Did you authorize HPV vaccination for your child in the past months?”. Due to the reduced sample size (N=694), we only consider this measurement as qualitative evidence.

^27^
As discussed further below, results are comparable under an LPM/OLS estimation.

^28^
The second dose of the MMR vaccine is offered approximately 3 years before the intervention, under the national vaccination program, and thus under the same conditions. It constitutes a conservative measure of vaccine hesitancy, since the most recent coverage data indicate an uptake consistently above 95% across all cohorts for the second dose of MMR (whereas the most recent figure for the second dose of the HPV vaccine is only 82.9%).

^29^
Supporting Information S1: Section M in the Appendix describes in detail the missingness in the data and the imputation. Power calculations that take this into account are reported in Section L of the Appendix.

^30^
The coefficients of baseline covariates are omitted to enhance readability and reported in Supporting Information S1: Section G of the Appendix.

^31^
The BH procedure is applied to *p*‐values obtained by pooling test statistics from multiple imputations (MI), following the procedure described by Rubin (1987), which takes into account the variability of estimates across imputations. Significant *p*‐values, instead, do not pass the BH procedure if the latter is applied directly to *p*‐values obtained by comparing T‐statistics to a standard distribution manually without taking into account the variability across imputation, a more conservative approach. Nevertheless, all the hypotheses and their tests within strata were pre‐registered, as well as the plan to independently sample individuals in each stratum. Since sampling variance drives the probability of false positives as the number of hypotheses increases, we believe the correct number of hypotheses should not be obtained by multiplying by the number of strata. In terms of outcomes, we effectively have one outcome measured with two indicators, and the two measures are almost perfectly correlated in the subsample of survey respondents, where correlation can be assessed directly (see Supporting Information S1: Section O in the Appendix).

^32^
While this specification exploits the covariates on the entire sample in every comparison, we still consider Equation (1) our main specification for comparability reasons, since it is the model used for causal forest estimation in our heterogeneity analysis (Section 6) and to discuss some of our mechanism analysis (Section 7).

^33^
This analysis is not explicitly included in the pre‐registered pre‐analysis plan if not under the more generic intention to investigate heterogeneity along various indicators. The rationale for comparing respondents and non‐respondents stems from observing our main results and lies in investigating how responding relates to engagement with our campaign.

^34^
The RR sample, however, is limited in size, and might be driving the statistical insignificance of some comparisons.
