# Supplementary material for: Targeting Vaccine Information Framing to Recipients' Education: A Randomized Trial
Source: Health Econ. 2025 Sep 17;34(12):2317–37. doi: 10.1002/hec.70036 (PMC12579523; doi:10.1002/hec.70036)
Supplement: Supplementary file 1 — Supporting Information S1: hec70036‐sup‐0001‐suppl‐data.pdf. [file HEC-34-2317-s001.pdf]

ONLINE APPENDIX

A Additional background information

A.1 The HPV vaccine in Sweden

The following figure shows the evolution of the HPV vaccine uptake (first dose) in Stockholm County relative to the national average, by birth cohort and gender. For each birth cohort, the uptake is measured one year after the vaccine has been offered in school (grade 5) as part of the national vaccination program: the vaccine is offered at approximately age 12. After several childhood vaccinations in pre-school age, offered through vaccination clinics (free of charge), children are offered the second dose of the MMR vaccine in schools between the 1st and 2nd grade, then the HPV vaccine in the 5th grade, and finally the fourth dose against diphtheria, tetanus and pertussis between the 8th and 9th grade. Therefore, parents already know the school-based vaccination program by the time they are offered the HPV vaccine.[35]

In school classes where vaccinations are offered, children are handed in paper consent forms that detail the nature of the vaccine and some basic information. The English version is presented in Figure A.1. The form clearly states that it is valid for both HPV doses and does not specify a date for inoculation: this is because the school nurse is based on school premises, and the vaccine can be inoculated on different days, ensuring that children who miss school days can be vaccinated as well. The consent form must also be returned by a pre-specified date in case the consent is negated. Parents who decide to deny their consent can still arrange vaccination later, free of charge, if the child is less than 18. After that, the vaccine can be obtained in private vaccine clinics, paying for its cost. Still, our leaflets underline the importance of vaccinating early, before any sexual activity.

[35] The full vaccination schedule can be consulted on the Swedish Public Health Agency's website: https://www.folkhalsomyndigheten.se/the-public-health-agency-of-sweden/communicable-disease-control/vaccinations/vaccination-programmes/

Figure A.1: Schools' reminder and consent form

Information and offer of vaccination against HPV

In 2010, vaccinations against human papillomavirus (HPV) for girls were introduced in the Swedish childhood vaccination programme. As of August 2020, the HPV vaccination will be offered for all children in year 5 of compulsory school. An HPV infection can cause cellular changes which in the long term can lead to cancer of the cervix, throat, genital organs and anus.
For the vaccine to have full effect, it is given twice over an interval of at least 6 months. If you start the course of vaccinations at 15 years or older, three doses are required.
Using this form, you can notify us of whether your child is to be vaccinated or not by ticking the boxes below. If you do not provide your consent, your child cannot be vaccinated.
This consent includes all doses. In preparation of the vaccinations, we ask that you also answer the questions in the health declaration form below. Please notify us if circumstances change in between doses. If you want more information, or you have further questions, please contact your school nurse.

Vaccination for HPV ***Return to school nurse no later than***

Child's name ***Personal identity number Class***

☐ Yes, I consent to my child being vaccinated

☐ No, I don't want my child to be vaccinated

Health declaration *(If you answered No to vaccination, you do not need to answer)*

– Does the child suffer from severe allergy? Yes ☐ No ☐
– Has the child ever had a serious allergic reaction to any other vaccine? Yes ☐ No ☐
– Does the child have any serious chronic illness? Yes ☐ No ☐

you answered yes to any of the questions above, please leave supplementary information on the back of the sheet.

Signatures Place: Date:

Signature of parent/guardian Name in block letters Telephone, daytime

---

Signature of parent/guardian Name in block letters Telephone, daytime

To be signed by both guardians if they have shared custody.

In accordance with the Act on Registers of National Vaccination Programmes (2012:453) all vaccinations within the childhood vaccination programme are reported in the national vaccination registry. More information is available at www.folkhalsomyndigheten.se

form vacc in school.png

Figure A.2: HPV vaccine (first dose) uptake over time: Stockholm County *vs* Swedish average

vs Swe coverage.png

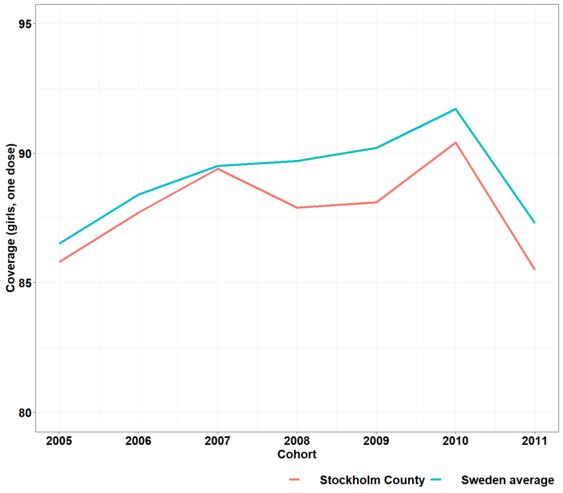

(A.) Girls

vs swe coverage boys.png

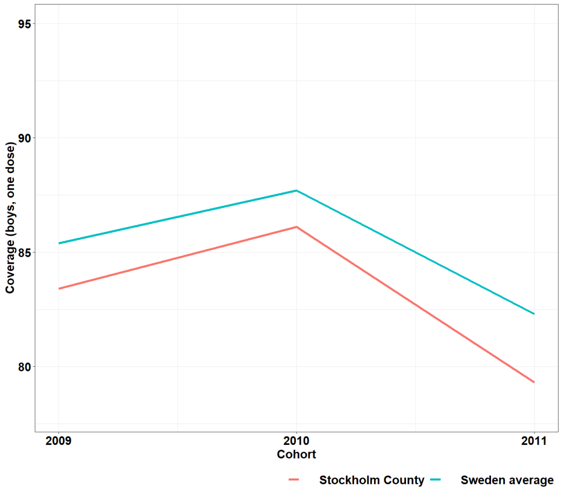

(B.) Boys

Notes: Data are publicly available on the website of the *Public Health Agency of Sweden*: https://www.folkhalsomyndigheten.se/globalassets/statistik-uppfoljning/smittsamma-sjukdomar/hpv/statistik-om-hpv-vaccinationer_svevac-2015.pdf.

A.2 Trust indicators: Sweden relative to Western Europe

Trust in government and health institutions, as well as attitudes towards science, can impact the success of informational campaigns on vaccines and, thus, the external validity of our findings. This is especially important in light of COVID-19, which impacted countries and these indicators differently. How does Sweden fare relative to other countries?

Table A.1 reports some key indicators from the "Wellcome Global Monitor 2020: Covid-19" survey (Wellcome Trust, 2021), comparing Sweden with other Nordic countries (Norway, Denmark) and with the Western Europe average. Sweden is generally comparable to them when it comes to trusting science and scientists, but the effects of the COVID-19 pandemic are visible. In particular, Swedes report the lowest trust in their government, the lowest reliance on doctors and nurses when it comes to decisions related to COVID-19, and the lowest acceptance of COVID-19 vaccines (the survey was run before those became available). In many regards, Swedish figures are closer to Western Europe than other Scandinavian countries, which are reported to be among the most trusting of governments and science worldwide.

Table A.1: Trust of scientists, doctors and authorities: Sweden and Europe

Wellcome question	Indicator	Sweden	Western Europe (avg)	Norway	Denmark
**Attitudes towards science**					
W1	(%) who says to know science a lot	17.98	15.32	18.20	24.29
W5C	(%) who trusts scientists in the country a lot	60.07	59.13	72.03	62.75
W6	(%) who trusts science a lot	62.20	58.55	63.57	55.15
W7B	(%) that believes a lot of scientists work to benefit the public	33.46	45.82	58.32	51.81
W8	(%) who believes most scientists benefit people in their country	69.12	49.57	64.15	61.81
W9	(%) who believes most scientists benefit people like them	67.05	51.20	56.89	67.78
**Attitudes towards the government**					
W5B	(%) who trusts their government a lot	24.74	27.69	61.66	38.23
W7C	(%) that believes the government values the opinion of scientists/experts	30.16	21.50	27.95	24.29
**(%) basing a lot of COVID-related decisions on advice from:**					
W15_A	The national government	47.05	43.41	74.61	56.68
W15_C	The WHO	60.93	56.95	84.74	71.12
W15_D	Doctors and nurses	70.07	75.90	92.75	83.08
WP21768	(%) would have accepted a free COVID vaccine	58.93	64.01	73.73	84.60

Notes: The source is Wellcome Trust (2021). Beyond "A lot", respondents could indicate "Some", "Not much", "Nothing at all" or "Don't know".

B Invitation letter

Guardians' attitudes and willingness to vaccinate children against HPV

Any questions?
You are welcome to contact us!

Questions on information delivery
Statistics Sweden reporting service
010-479 63 30
enkat@scb.se
SCB, INS/IHU, 701 89 Örebro
www.scb.se

Opening hours
Monday-Thursday: 8.00 – 21.00
Friday: 8.00 – 17.00
Sunday: 16.00 – 21.00

Questions on the study
Lisen Arnheim Dahlström
lisen.arnheim.dahlstrom@ki.se
Alice Dominici
alice.dominici@eui.eu

Vill du svara på svenska?
På hemsidan kan du välja huruvida du vill svara på svenska eller engelska. Du kan svara på frågorna här:
www.insamling.scb.se
Logga in med ditt användarnamn och lösenord. Du behöver inte svara på alla frågor på en gång utan kan också logga in flera gånger.

SCB describes Sweden
Statistics Sweden provides society with statistics for decision-making, debate and research. We do this on behalf of the government, authorities, researchers and the business community. Our statistics contribute to a fact-based public debate and well-founded decisions.

Dear guardian,

You are receiving this letter because researchers at Karolinska Institute want to investigate attitudes of guardians towards the HPV vaccination in the context of the national vaccination program, and the role of information.
You are one of 7,616 guardians who were randomly selected to participate in the survey. In September, you will be offered to vaccinate your child against HPV as part of the national vaccination program.

You have received an information sheet about the HPV vaccine in this envelope. The information sheet is available in several versions. The information sheet that guardians receive is randomly selected. The researchers assure that the information you receive is always truthful: if you want to know more about the study or the information you received, contact the researchers Lisen A. Dahlström or Alice Dominici. We would be grateful if you could read the short information sheet and then answer some questions.

The survey is conducted by Statistics Sweden on behalf of Karolinska Institute. On the next page, you will find more information about the survey.

Your answers are important

It is voluntary to participate in the survey, but we hope you want to participate, because your answers are very important. You help to give a complete picture of the willingness to vaccinate and we are interested in all opinions.

How you can answer

First, read the information sheet on the HPV vaccine contained in the envelope. Then, go to **www.insamling.scb.se** to answer the questions. Online, you can answer in Swedish or English.

Your credentials are:

**Användarnamn:**	
**Lösenord:**	

If you instead choose to answer on paper, you can send the paper form in the postage-free reply envelope that you received in this letter.

You can log in several times and save your progress each time.

Sincerely,

Joakim Stymne	Lisen Arnheim Dahlström
General Director *SCB*	*Principal Investigator, Karolinska Institute*
	Alice Dominici, *Project leader, Karolinska Institute*

SCB Karolinska Institutet

Consent to participate in the study

By answering this survey, you confirm your consent to participate in this study. This has been approved by the Ethics Review Board with decision number 2021-01225. You can cancel your participation in the study at any time without any consequences, and you do not need to state why. To cancel your participation, please contact the person responsible for the study (lisen.arnheim.dahlstrom@ki.se). Everyone who chooses to participate in this survey will be invited to a follow-up survey this autumn. This means that Statistics Sweden saves information about who participated until the follow-up study is completed.

How are my answers protected?

When survey results are published, your individual answers will never be identifiable. Information about individuals' personal and financial circumstances is protected by confidentiality and everyone who works with the survey has a duty of confidentiality. Confidentiality applies according to ch. Section 8 of the Public Access to Information and Secrecy Act (2009: 400). The same confidentiality regulations apply to Karolinska Institute.

How is personal data processed?

SCB is responsible for personal data processing performed by SCB. Karolinska Institute is responsible for personal data processing performed by Karolinska Institute. Rules for personal data processing are laid out in the EU Data Protection Regulation, in the Act (2001: 99) and in Ordinance (2001: 100) on official statistics. In addition, law (2018: 218) provides supplementary provisions to the EU Data Protection Regulation and to regulations that have been issued in connection with that law. More information on how SCB processes personal data can be found at www.scb.se/personal data. You have the right to receive a free copy, in the form of a so-called register extract, of the personal data processed by SCB in your capacity as personal data controller. More information can be found at www.scb.se/registerutdrag. If you believe that SCB has processed your personal data in a way that violates the EU Data Protection Regulation, you have, under certain conditions, the right to have the data deleted. You have the same rights towards Karolinska Institute. The number at the top, in the middle of the first page of the letter, allows SCB to see who has responded during the data collection, among other things.

Do you have questions on personal data?

Contact the data protection officer:
SCB: 010-479 40 00, dataskyddsombud@scb.se,
701 89 Örebro
Karolinska Institutet: 08-524 864 73,
dataskyddsombud@ki.se,
UF Universitetsförvaltningen, UF JA JUR, 171 77
Stockholm

Possible consequences and risks of participation

As your answers are analyzed anonymously and presented at the aggregate level, there is no risk that your answers may end up in the wrong hands or be used in an unintended way. There is no link to information that could reveal your identity in an unwanted way.

Where will the results be published?

The results of the study will be summarized scientifically and published in scientific journals. A summary will also be published on Karolinska Institute's website. If you are interested in results, you can contact the researchers by e-mail (alice.dominici@eui.eu).

Who uses the answers and how?

SCB processes and deidentifies the data and then submits them to Karolinska Institute for further processing and analysis. SCB and Karolinska Institute may disclose the information to researchers or others who produce statistics. Such research and statistical activities are also covered by confidentiality. In order not to ask more than necessary, we will supplement your answers with information that is already available at SCB. This is information about your age, marital status, education, occupation, income, number of children and any year of immigration. We also supplement your answers with information about the child who might be vaccinated. It is information about gender, the child's order of birth, if the child is your biological or adopted child and a deidentified code of the school attended by the child. Information on vaccinations is obtained from the Swedish Public Health Agency's vaccination register. We also supplement your answers with information about the child's other guardians. It is information about education, occupation and income. Your answers and the other information are saved at SCB. Submitted information is stored at SCB for 12 months after the completion of the assignment.

C List of variables from population registers

C.1 List of variables

The following variables are extracted and elaborated from Swedish population registers:

For both parents:

- Demographic variables: is married (dummy), was born in Sweden (dummy), country of origin, age;
- Education variables: highest educational attainment, grade at national high school examination, graduation year, has received medical education (dummy), has received scientific education (dummy), has received a numerical education (dummy);
- Labour variables: is an active worker (dummy), is retired (dummy), is a medical doctor (dummy), has an occupation in healthcare (dummy), is a nurse or a dentist, has an occupation in research;
- Economic variables: disposable income (earned from labour income and any property income in the 12 months before treatment), capital income (net financial gains in the 12 months before treatment), amount of government transfers received in the 12 months before treatment;
- **Only for immigrant parents:** has received any medical education (dummy), has received any scientific education (dummy), has received any numeric education (dummy), has received a formal degree in Sweden, years since immigration date. *Note*: the educational variables are extracted from immigration registers and are meant to account for the education received prior to immigration.

Child: is female (dummy), is adopted (dummy), birth order (relative to the mother's children), number of MMR vaccine doses received at baseline.

School: anonymized code, anonymized code for the municipality where it is located.

C.2 Classification of education fields

We classify education as:

Scientific: Natural sciences high school track. Degrees and specializing degrees in any of the following subjects: Biology, Chemistry, Biochemistry, Physics, Environmental Sciences, Geology, Mathematics, Statistics, Engineering, Food Sciences, Agronomy, Botany, Veterinary science, Medicine, Nursing, Pharmacy, Dietology, Logopedy, Naturopathy;
Medical: Degrees and specializing degrees in any of the following subjects: Veterinary science, Medicine, Nursing, Pharmacy, Dietology, Logopedy, Naturopathy;
Numerical: Degrees and specializing degrees in any of the following subjects: Mathematics, Statistics, Engineering, Economics, Finance, Business.

D Pre-registered analyses and deviations

The trial was pre-registered on the AEA RCT Registry in August 2021 as "Education, Immigration and HPV Vaccination: an Informational Randomized Trial", ID AEARCTR-0007668.

The Pre-Analysis Plan (PAP) reports the intended treatments, the stratified design, the envisaged sample sizes, the hypotheses to be tested within each stratum, the outcomes and secondary analyses. This section lists deviations from the PAP and the reasons for deviating:

- **Primary outcomes:** The PAP reports beliefs on vaccines as a primary outcome. In this paper, instead, those analyses are reported in the Appendix, as they do not contribute as clearly to the main results as other analyses – especially heterogeneity analyses obtained with causal forests and the study of the determinants of survey response.
- **Sample sizes:** We obtained a larger sample size than expected for the main analysis (7616 instead of 7500) and a lower response rate in the endline survey, which prevented us from running a complete analysis of mechanisms as pre-registered (suggestive, qualitative results are still reported in this Appendix).
- **Choice of the estimator:** We did not specify an estimator for our main analyses in the PAP, but we did specify the inclusion of baseline covariates to increase precision, providing

their list. As a result, while we choose logit as the preferred estimator given our binary primary outcome and its greater precision, we include the LPM (including the "structural" specification) as robustness checks, as detailed in Section 5 of the main text.

- **(T *vs* C) hypothesis:** The PAP reports three hypotheses to test within each stratum: each individual treatment against the control (T1 *vs* C and T2 *vs* C), the two treatments against each other (T2 *vs* T1) and any treatment, T1 or T2, denoted T, against the sole reminder in the control group. The tests of this last hypothesis are not included in the main analysis for improved readability, but the results and their interpretation are mentioned. The full results are in this Appendix instead, in Section H.3.

- **Heterogeneity:** We did not specify a method for performing heterogeneity analyses in the PAP. We eventually chose causal forests, as they entail minimal choices from the researchers (all pre-treatment covariates could be assessed at the same level) and are non-parametric.

- **Determinants of participation:** while pre-registered, we did not anticipate including the analysis of determinants of participation (i.e., comparing baseline covariates between survey respondents and non-respondents) in the heterogeneity analysis section of the paper. We did so when the main analysis suggested that these differences were key to interpreting our ITT estimates. Moreover, rather than using R^2 estimates that summarised the overall impact of all covariates, we adopted more specific balance tests for each covariate as suggested by attendants of presentations and discussants.

- **School FE dummies:** while we initially envisaged to include school FE dummies in our models to account for school-level differences, this was not feasible in practice. Since our sampling was not clustered within schools but at the individual level, we ended up having too many schools relative to the number of observations. We instead control for municipality dummies.

E Balance and attrition

For each baseline covariate included in the main analysis X, the following tables report, separately for the full sample and those of respondents to the first survey, the Absolute Standardized Difference

(ASD), computed as:

$$ASD(X) \equiv \frac{|\bar{X}_T - \bar{X}_C|}{\sqrt{Var_T(X) + Var_C(X)}}$$

Note that ASD is also known as Cohen's D and can be interpreted as the difference in means expressed in pooled standard deviations. Its statistical significance is assessed by performing a two-tailed T-test for differences in means on separate samples (i.e., taking into account that covariates in treatment and control groups have different variances). For variables observed only on a subgroup (e.g., high school grade), we computed means and ASD only where available.

Figure E.3 shows that attrition in answering the first survey (hence the sample composition in the full sample *vs* the sample of respondents) is not driven by treatment status.

Figure E.3: Attrition in the first survey by treatment

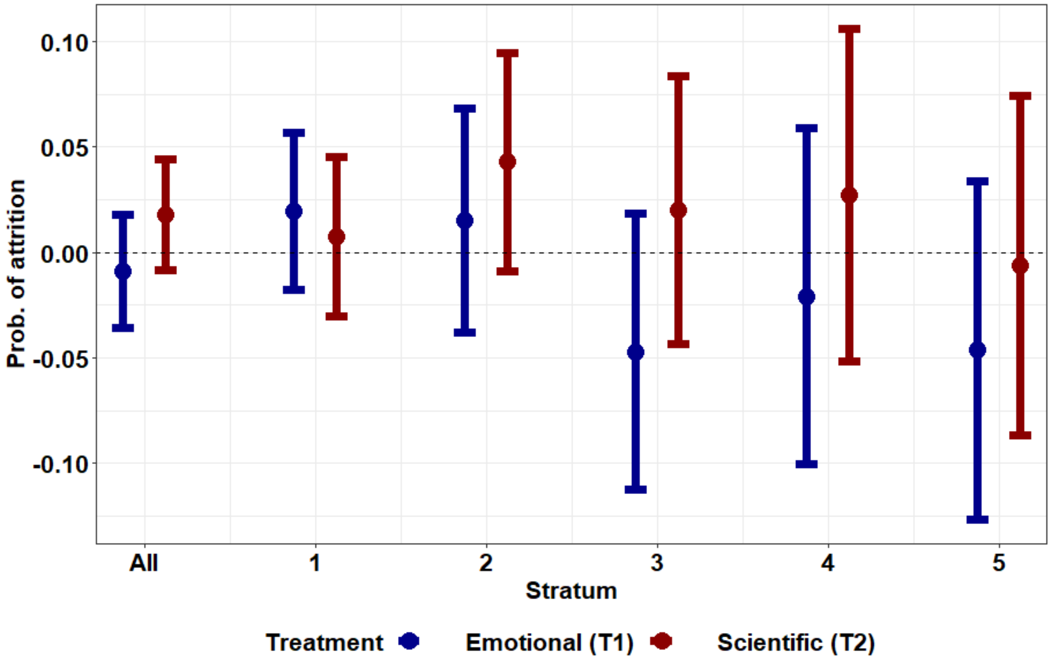

Notes: The figure shows estimates and 95% C.I. from OLS regressions where the dependent variable is a dummy for attrition (i.e., not responding to the first survey), and the regressors are the two treatment dummies (the control group only includes control units in both cases).

Table E.2: Balance table: overall sample (used to estimate main ITT results)

**Covariate**	**ASD: T1 *vs* C**	**Mean (C)**	**Mean (T1)**	**ASD: T2 *vs* C**	**Mean (T2)**
**Mother's characteristics**					
Age	0.019	41.095	40.933	0.014	40.977
Married (Dummy)	0.009	0.591	0.597	0.002	0.592
Capital income (Thousands SEK)	0.018	-7.571	519.213	0.017	63.581
Disposable income (Thousands SEK)	0.019	3,222.662	3,613.435	0.023	3,313.980
Scientific educ. (Dummy)	0.019	0.205	0.215	0.023	0.218
Medical educ. (Dummy)	0.018	0.134	0.143	0.021	0.145
Numerical educ. (Dummy)	0.008	0.163	0.167	0.012	0.157
High school grade	0.029	13.685	13.538	0.045	13.464
Job in research (Dummy)	0.008	0.005	0.004	0.030	0.002
N of children	0.034	2.084	2.128	0.094**	2.204
**Child's characteristics**					
Child order	0.022	1.029	1.024	0.041	1.040
Female (Dummy)	0.031	0.475	0.497	0.027	0.494
Doses of MMR	0.030	1.936	1.925	0.064*	1.912
**Father's characteristics**					
Age	0.036	45.178	44.881	0.062	44.673
Swedish citizen (dummy)	0.044	0.945	0.930	0.019	0.939
High school grade	0.081**	12.876	13.269	0.043	13.090
**Both parents' characteristics**					
Government transfers (Thousands SEK)	0.050	0.059	0.077	0.052	0.078
Job in healthcare (Dummy)	0.124***	0.091	0.147	0.068*	0.121
Job in research (Dummy)	0.034	0.012	0.018	0.009	0.011
**Immigrants' extra controls**					
Educ level (mother)	0.000	3.392	3.393	0.003	3.400
Educ level (father)	0.010	3.242	3.265	0.050	3.359

Notes: *** $p< 0.01$, ** $p< 0.05$, * $p< 0.10$ on two-tailed tests of difference in means on separate samples. The overall sample is used to estimate the main results (where the dependent variable is actual vaccination choice and the estimand is an ITT). Denoting treated units as T and untreated units as C, The Absolute Standardized Difference (ASD) for variable X is computed as: $ASD \equiv \frac{|\bar{X}_T - \bar{X}_C|}{\sqrt{Var_T(X)+Var_C(X)}}$.

Table E.3: Balance table: respondents sample (used to estimate secondary ATE results)

Covariate	ASD: T1 *vs* C	Mean (C)	Mean (T1)	ASD: T2 *vs* C	Mean (T2)
**Mother's characteristics**					
Age	0.013	42.518	42.420	0.016	42.401
Married (Dummy)	0.015	0.640	0.630	0.006	0.636
Capital income (Thousands SEK)	0.006	17.652	7.921	0.022	64.070
Disposable income (Thousands SEK)	0.039	3,634.945	3,749.006	0.040	3,786.175
Scientific educ. (Dummy)	0.019	0.205	0.216	0.065	0.243
Medical educ. (Dummy)	0.071	0.104	0.136	0.067	0.134
Numerical educ. (Dummy)	0.001	0.211	0.212	0.001	0.210
High school grade	0.050	14.373	14.584	0.092	13.963
Job in research (Dummy)	0.054	0.013	0.006	0.072	0.004
N of children	0.038	2.097	2.053	0.044	2.148
**Child's characteristics**					
Child order	0.027	1.019	1.014	0.042	1.028
Female (Dummy)	0.028	0.499	0.479	0.051	0.535
Doses of MMR	0.081	1.956	1.929	0.071	1.933
**Father's characteristics**					
Age	0.057	45.788	45.353	0.080	45.164
Swedish citizen (dummy)	0.024	0.947	0.939	0.024	0.954
High school grade	0.126**	13.300	13.850	0.006	13.270
**Both parents' characteristics**					
Government transfers (Thousands SEK)	0.018	0.062	0.056	0.008	0.065
Job in healthcare (dummy)	0.106*	0.071	0.114	0.133**	0.127
Job in research (dummy)	0.013	0.018	0.020	0.008	0.016
**Immigrants' extra controls**					
Educ level (mother)	0.049	3.814	3.931	0.012	3.785
Educ level (father)	0.013	3.474	3.504	0.073	3.646

Notes: *** p< 0.01, ** p< 0.05, * p< 0.10 on two-tailed tests of difference in means on separate samples. The sample of survey respondents is used to estimate the secondary results (where the dependent variables are self-reported intention to vaccinate and misconceptions about vaccines, and the estimand is an ATE). Denoting treated units as T and untreated units as C, The Absolute Standardized Difference (ASD) for variable X is computed as: $ASD \equiv \frac{|\bar{X}_T - \bar{X}_C|}{\sqrt{Var_T(X)+Var_C(X)}}$.

F Survey

The survey has been re-programmed by *Statistics Sweden* and administered via both their internal software (online) and on paper. It was available in English, Swedish, Persian, Arabic and Farsi. Before the questions, parents will see a screen containing the consent form for participation in the study.

F.1 First survey - administered right after treatment

1. Have you heard about HPV (Human Papilloma Virus) before receiving our letter?
   (Yes/No/I am not sure)

2. As of now, how likely is it that you will authorize HPV vaccination for your child in September?
   (Definitely not/ Unlikely/ Slightly unlikely/ I don't know yet/ Slightly likely/ Likely/ For sure)

3. Up until now, have you read any information on the HPV vaccine? (You can select multiple answers)
   Yes, I was given information from the school, my clinic or other health professionals / I am not sure or I don't remember/ I haven't received nor searched for any information on the HPV vaccine/ Other (open field)

4. If you have received information, from which of the following sources? (You can select multiple answers)
   School nurse/ 1177 (Swedish public information service)/ My local public clinic/ Social networks/ Radio and television/ Friends and family/ Other (open field)

5. If you have searched information, which of the following sources did you consult?
   School nurse/ 1177 (Swedish public information service)/ My local public clinic/ Social networks/ Radio and television/ Friends and family/ Other (open field)

6. Is any of your close friends and relatives a doctor or a health professional?
   Yes, a medical doctor/ Yes, a nurse or other health professional/ No

7. Before September, do you think you will look for more information about the HPV vaccine? (You can select multiple answers)
   Yes, from the school nurse/ Yes, from 1177/ Yes, from my local public clinic/ Yes, from friends and family/ Yes, on social networks/ Yes, on health authorities websites (Public Health Agency, World Health Organization etc.)/ Yes, on other websites and private blogs/ No, I am sufficiently informed already/ No, I am not interested/ Other (open field)

8. Please, indicate how much you agree with the following statements (Likert scale: Strongly agree/ Agree/ Somewhat agree/ Neither agree nor disagree/ Somewhat disagree/ Disagree/ Strongly disagree):
   - Vaccines weaken and overload the immune system
   - Vaccines can cause the disease against which they protect
   - Vaccines can produce serious side-effects
   - I trust the opinion of health professionals and health authorities about vaccines
   - I am an informed parent when it comes to vaccines

9. Please select the first option below:
   - First option
   - Second option

F.2 Second survey - administered at endline

Last summer, you received some information on the HPV vaccine. Recently, you had to decide whether to vaccinate your child against HPV. We'd like to ask a few more questions, and we thank you for participating in this study.

1. How important is it to you that your child (Likert scale: Not at all important/ Slightly important/ Moderately important/ Very important/ Extremely important):
   - Can become a parent one day
   - Does not have sex before marriage
   - Does not have any serious illness while young (less than 35)

- Does not have cancer while young (less than 35)
- Does not have any serious illness when older (more than 35)
- Does not have cancer when older (more than 35)
- Does not need to undergo invasive and distressing medical procedures

2. In your opinion, what is the effect of doing the HPV vaccine on the probability of these events (Scale (1-5): Decreases the probability/ Slightly decreases the probability/ No effect/ Slightly increases the probability/ Increases the probability):
   - Your child has sex before marriage
   - Your child develops a serious health issue before age 35
   - Your child gets cancer before age 35
   - Your child develops a serious health issue when older than 35
   - Your child gets cancer after age 35
   - Your child needs to undergo invasive and distressing medical procedures

3. Please, indicate how much you agree with the following statements (Likert scale: Strongly agree (1)/ Agree (2)/ Somewhat agree (3)/ Neither agree nor disagree (4)/ Somewhat disagree (5)/ Disagree (6)/ Strongly disagree (7)):
   - Vaccines weaken and overload the immune system
   - Vaccines can cause the disease against which they protect
   - Vaccines can produce serious side-effects
   - I trust the opinion of health professionals and health authorities about vaccines
   - I am an informed parent when it comes to vaccines

G Main analysis: baseline covariates' coefficients

In this section, we show estimates from our main analysis including coefficients for baseline covariates. We begin with our ITT estimates where the outcome is actual HPV vaccination uptake and then proceed to ATE estimates among respondents, where the outcome is self-reported intention to vaccinate. Results are presented by stratum.

G.1 Primary analysis: actual vaccination (ITT)

Table G.4: ITT estimates on actual vaccination: stratum 1

	(T1 vs C)		(T2 vs C)		(T2 vs T1)	
	**Coeff.**	**(s.e.)**	**Coeff.**	**(s.e.)**	**Coeff.**	**(s.e.)**
Treatment (dummy)	-0.016	(0.02)	-0.013	(0.02)	0.002	(0.017)
Mother's age (years)	-0.002	(0.002)	0.001	(0.002)	-0.003*	(0.002)
Father's age (years)	0.004**	(0.002)	0.001	(0.002)	0.004**	(0.002)
Child order (for mother)	0.166**	(0.072)	-0.048	(0.041)	-0.095**	(0.038)
HH disposable income (th. SEK)	-0.003	(0.009)	0	(0.009)	0.003	(0.008)
MMR vaccine doses received	0.062**	(0.022)	0.033	(0.023)	0.042**	(0.02)
Mother's education level	-0.006	(0.005)	0.004	(0.005)	0.005	(0.005)
Father's education level	-0.006	(0.006)	-0.001	(0.006)	-0.004	(0.006)
Swedish father (dummy)	-0.029	(0.055)	0.011	(0.056)	-0.008	(0.05)
Female child	0.073***	(0.019)	0.057**	(0.019)	0.064***	(0.017)
HH capital income (th. SEK)	0.01	(0.027)	0	(0.031)	0.012	(0.021)
Married mother (dummy)	-0.005	(0.023)	0.001	(0.024)	0	(0.022)
Mother has medical educ. (dummy)	0.027	(0.047)	-0.01	(0.056)	0.005	(0.046)
Mother n. of children	-0.001	(0.007)	0.001	(0.007)	-0.001	(0.006)
Mother has numeric educ. (dummy)	0.064**	(0.032)	0	(0.042)	-0.002	(0.034)
Parent has medical occupation (dummy)	0.049**	(0.024)	-0.013	(0.029)	0.011	(0.023)
Parent has research occupation (dummy)	0.114	(0.27)	0.071	(0.443)	-0.031	(0.235)
Mother has scientific educ. (dummy)	0.012	(0.043)	0.057	(0.045)	0.023	(0.039)
HH welfare transfers (SEK)	-0.013	(0.016)	0.001	(0.016)	-0.004	(0.014)
Origin dummy: Eritrea	-0.066	(0.047)	0.026	(0.047)	0.057	(0.045)
Origin dummy: Iraq	-0.081**	(0.032)	-0.015	(0.04)	0.019	(0.038)
Origin dummy: Iran	-0.001	(0.038)	0.094**	(0.043)	0.118**	(0.04)
Origin dummy: Somalia	-0.377***	(0.047)	-0.314***	(0.054)	-0.293***	(0.05)
Origin dummy: Syria	-0.026	(0.032)	0.021	(0.04)	0.062	(0.038)

Notes: *** $p< 0.01$, ** $p< 0.05$, * $p< 0.10$. Results are estimated on the entire sample of invited subjects: they can be interpreted as an ITT effect for the entire population of reference. Immigrants in stratum 1 are mothers born in Iraq, Iran, Syria, Afghanistan, Eritrea or Somalia. Country of origin dummies are relative to Afghanistan (default category).

Table G.5: ITT estimates on actual vaccination: stratum 2

	**(T1 vs C)**		**(T2 vs C)**		**(T2 vs T1)**	
	**Coeff.**	**(s.e.)**	**Coeff.**	**(s.e.)**	**Coeff.**	**(s.e.)**
Treatment (dummy)	0.037	(0.025)	0.057**	(0.024)	0.029	(0.021)
Mother's age (years)	-0.001	(0.002)	-0.003	(0.002)	0.001	(0.002)
Father's age (years)	-0.001	(0.002)	0	(0.002)	-0.001	(0.002)
Child order (for mother)	0.112	(0.082)	0.093	(0.069)	0.25*	(0.132)
HH disposable income (th. SEK)	0.013	(0.01)	0.01	(0.009)	0.011	(0.009)
MMR vaccine doses received	0.146***	(0.03)	0.211***	(0.026)	0.178***	(0.024)
Swedish father (dummy)	0.074**	(0.03)	0.051*	(0.028)	0.066**	(0.026)
Female child	0.085***	(0.024)	0.085***	(0.023)	0.046**	(0.02)
Married mother (dummy)	0.066**	(0.025)	0.045*	(0.024)	0.065**	(0.022)
Mother has medical educ. (dummy)	-0.229	(0.156)	-0.179	(0.126)	-0.055	(0.083)
Mother n. of children	-0.01	(0.01)	-0.008	(0.01)	-0.013	(0.009)
Mother has numeric educ. (dummy)	-0.064	(0.044)	0.022	(0.037)	0.011	(0.036)
Parent has medical occupation (dummy)	0.01	(0.035)	-0.014	(0.034)	0.006	(0.029)
Parent has research occupation (dummy)	0.048	(0.367)	0.011	(0.62)	0.096	(0.456)
Mother has scientific educ. (dummy)	0.115	(0.072)	0.067	(0.069)	-0.009	(0.067)
HH welfare transfers (SEK)	-0.051*	(0.027)	-0.018	(0.026)	-0.063**	(0.021)

Notes: *** p< 0.01, ** p< 0.05, * p< 0.10. Results are estimated on the entire sample of invited subjects: they can be interpreted as an ITT effect for the entire population of reference. Stratum 2 comprises mothers with at most 3 years of high school: this corresponds to Swedish *högstadiet* (grades 7-9), the last compulsory grades under Swedish law.

Table G.6: ITT estimates on actual vaccination: stratum 3

	**(T1 vs C)**		**(T2 vs C)**		**(T2 vs T1)**	
	**Coeff.**	**(s.e.)**	**Coeff.**	**(s.e.)**	**Coeff.**	**(s.e.)**
Treatment (dummy)	-0.048**	(0.022)	0.004	(0.021)	0.041**	(0.020)
Mother's age (years)	-0.003	(0.003)	-0.001	(0.003)	-0.003	(0.003)
Father's age (years)	0.001	(0.002)	-0.003	(0.002)	0.001	(0.002)
Child order (for mother)	-0.046	(0.103)	0.092	(0.1)	0.013	(0.074)
HH disposable income (th. SEK)	0.026**	(0.01)	0.035***	(0.009)	0.031***	(0.009)
MMR vaccine doses received	0.127***	(0.031)	0.145***	(0.03)	0.155***	(0.026)
Swedish father (dummy)	0.024	(0.028)	0.059*	(0.031)	0.033	(0.026)
Female child	0.063**	(0.022)	0.026	(0.021)	0.043**	(0.02)
HH capital income (th. SEK)	-0.037**	(0.016)	-0.013	(0.022)	-0.028**	(0.013)
Married mother (dummy)	0.002	(0.024)	-0.024	(0.022)	-0.015	(0.021)
Mother has medical educ. (dummy)	0.032	(0.05)	-0.05	(0.073)	0.012	(0.051)
Mother n. of children	0.001	(0.012)	0.002	(0.01)	0.005	(0.01)
Mother has numeric educ. (dummy)	-0.012	(0.031)	0.032	(0.025)	0.014	(0.025)
Parent has medical occupation (dummy)	0.058**	(0.029)	0.071**	(0.022)	0.069**	(0.023)
Parent has research occupation (dummy)	0.138***	(0.011)	0.068	(0.657)	0.06	(0.342)
Mother has scientific educ. (dummy)	-0.075	(0.057)	0.001	(0.043)	-0.038	(0.048)
HH welfare transfers (SEK)	-0.086**	(0.028)	0.009	(0.029)	-0.03	(0.027)

Notes: *** p< 0.01, ** p< 0.05, * p< 0.10. Results are estimated on the entire sample of invited subjects: they can be interpreted as an ITT effect for the entire population of reference. Mothers in stratum 3 completed high school (*gymnasium*, grades 10-12), which is not compulsory and comprises different tracks, including vocational ones.

Table G.7: ITT estimates on actual vaccination: stratum 4

	(T1 vs C)		(T2 vs C)		(T2 vs T1)	
	**Coeff.**	**(s.e.)**	**Coeff.**	**(s.e.)**	**Coeff.**	**(s.e.)**
Treatment (dummy)	-0.016	(0.026)	-0.021	(0.025)	-0.010	(0.023)
Mother's age (years)	-0.002	(0.003)	0.001	(0.003)	0.001	(0.003)
Father's age (years)	0.001	(0.003)	-0.003	(0.003)	-0.001	(0.002)
Child order (for mother)	0.03	(0.103)	0.004	(0.117)	1.517	(176.401)
HH disposable income (th. SEK)	0.014	(0.009)	0.005	(0.008)	0.011	(0.008)
MMR vaccine doses received	0.092**	(0.036)	0.132***	(0.035)	0.141***	(0.031)
Swedish father (dummy)	0.017	(0.035)	0.033	(0.038)	0.051	(0.034)
Female child	0.042*	(0.025)	0.024	(0.025)	0.026	(0.023)
HH capital income (th. SEK)	-0.007	(0.014)	-0.015	(0.009)	-0.016	(0.011)
Married mother (dummy)	-0.012	(0.026)	-0.005	(0.026)	0.001	(0.024)
Mother has medical educ. (dummy)	0.007	(0.065)	0.091**	(0.032)	0.041	(0.048)
Mother n. of children	0.015	(0.015)	-0.003	(0.015)	0.005	(0.014)
Mother has numeric educ. (dummy)	0	(0.031)	0.014	(0.03)	-0.021	(0.031)
Parent has medical occupation (dummy)	0.067**	(0.033)	-0.039	(0.052)	-0.035	(0.046)
Parent has research occupation (dummy)	-0.2	(0.165)	0.113***	(0.012)	-0.084	(0.122)
Mother has scientific educ. (dummy)	-0.006	(0.046)	-0.004	(0.046)	0.017	(0.039)
HH welfare transfers (SEK)	0.051	(0.039)	-0.019	(0.045)	0.024	(0.041)

Notes: *** $p < 0.01$, ** $p < 0.05$, * $p < 0.10$. Results are estimated on the entire sample of invited subjects: they can be interpreted as an ITT effect for the entire population of reference. Mothers in stratum 4 have attended university, but not beyond an undergraduate degree.

Table G.8: ITT estimates on actual vaccination: stratum 5

	(T1 vs C)		(T2 vs C)		(T2 vs T1)	
	**Coeff.**	**(s.e.)**	**Coeff.**	**(s.e.)**	**Coeff.**	**(s.e.)**
Treatment (dummy)	-0.016	(0.026)	-0.021	(0.025)	-0.010	(0.023)
Mother's age (years)	-0.002	(0.003)	0.001	(0.003)	0.001	(0.003)
Father's age (years)	0.001	(0.003)	-0.003	(0.003)	-0.001	(0.002)
Child order (for mother)	0.03	(0.103)	0.004	(0.117)	1.517	(176.401)
HH disposable income (th. SEK)	0.014	(0.009)	0.005	(0.008)	0.011	(0.008)
MMR vaccine doses received	0.092**	(0.036)	0.132***	(0.035)	0.141***	(0.031)
Swedish father (dummy)	0.017	(0.035)	0.033	(0.038)	0.051	(0.034)
Female child	0.042*	(0.025)	0.024	(0.025)	0.026	(0.023)
HH capital income (th. SEK)	-0.007	(0.014)	-0.015	(0.009)	-0.016	(0.011)
Married mother (dummy)	-0.012	(0.026)	-0.005	(0.026)	0.001	(0.024)
Mother has medical educ. (dummy)	0.007	(0.065)	0.091**	(0.032)	0.041	(0.048)
Mother n. of children	0.015	(0.015)	-0.003	(0.015)	0.005	(0.014)
Mother has numeric educ. (dummy)	0	(0.031)	0.014	(0.03)	-0.021	(0.031)
Parent has medical occupation (dummy)	0.067**	(0.033)	-0.039	(0.052)	-0.035	(0.046)
Parent has research occupation (dummy)	-0.2	(0.165)	0.113***	(0.012)	-0.084	(0.122)
Mother has scientific educ. (dummy)	-0.006	(0.046)	-0.004	(0.046)	0.017	(0.039)
HH welfare transfers (SEK)	0.051	(0.039)	-0.019	(0.045)	0.024	(0.041)

Notes: *** $p < 0.01$, ** $p < 0.05$, * $p < 0.10$. Results are estimated on the entire sample of invited subjects: they can be interpreted as an ITT effect for the entire population of reference. Mothers in stratum 4 have attended university beyond an undergraduate degree (postgraduate education).

G.2 Secondary analysis: vaccination intentions among survey respondents (ATE)

Table G.9: ATE estimates on vaccination intentions: stratum 1

	**(T1 vs C)**		**(T2 vs C)**		**(T2 vs T1)**	
	**Coeff.**	**(s.e.)**	**Coeff.**	**(s.e.)**	**Coeff.**	**(s.e.)**
Treatment (dummy)	-0.039	(0.053)	-0.003	(0.047)	0.003	(0.048)
Mother's age (years)	0.008	(0.006)	0.003	(0.006)	0.009	(0.006)
Father's age (years)	-0.002	(0.005)	-0.002	(0.004)	-0.003	(0.005)
Answered in Swedish (dummy)	-0.021	(0.081)	-0.138**	(0.05)	-0.16**	(0.059)
Child order (for mother)	2.268	(587.3)	314.11	(225.881)	2.382	(351.951)
HH disposable income (th. SEK)	-0.017	(0.026)	0.012	(0.021)	-0.003	(0.021)
MMR vaccine doses received	-0.08	(0.068)	-0.074	(0.19)	-0.042	(0.06)
Mother's education level	-0.018	(0.014)	-0.003	(0.014)	-0.012	(0.014)
Father's education level	0.004	(0.018)	0.007	(0.016)	0.034**	(0.017)
Swedish father (dummy)	0.204	(3.924)	0.005	(0.15)	0.149*	(0.079)
Female child	0.029	(0.05)	-0.015	(0.046)	-0.023	(0.046)
HH capital income (th. SEK)	0.111	(0.129)	0.007	(0.1)	0.125	(0.11)
Married mother (dummy)	0.036	(0.076)	-0.068	(0.056)	-0.023	(0.063)
Mother has medical educ. (dummy)	-0.059	(0.132)	0.019	(0.09)	-0.042	(0.112)
Mother n. of children	-0.006	(0.023)	-0.014	(0.02)	-0.024	(0.02)
Mother has numeric educ. (dummy)	-0.038	(0.082)	-0.001	(0.077)	-0.158*	(0.086)
Parent has medical occupation (dummy)	0.121*	(0.065)	-0.071	(0.072)	-0.045	(0.075)
Parent has research occupation (dummy)	0.21	(1.406)	0.209	(0.734)	0.22	(0.397)
Mother has scientific educ. (dummy)	0.123	(0.08)	-0.025	(0.082)	0.129*	(0.072)
HH welfare transfers (SEK)	-0.052	(0.049)	-0.043	(0.033)	0	(0.045)
Origin dummy: Eritrea	-0.079	(0.142)	-0.178	(0.141)	-0.128	(0.119)
Origin dummy: Iraq	-0.173**	(0.081)	-0.119	(0.086)	-0.111	(0.089)
Origin dummy: Iran	0.034	(0.089)	0.142*	(0.076)	0.106	(0.089)
Origin dummy: Somalia	-0.244	(0.175)	0.05	(0.102)	-0.095	(0.157)
Origin dummy: Syria	-0.038	(0.081)	-0.012	(0.089)	-0.006	(0.09)

Notes: *** p< 0.01, ** p< 0.05, * p< 0.10. Results are estimated on the sample of respondents to the first survey: they can be interpreted as an ATE effect for this specific subpopulation. Immigrants in stratum 1 are mothers born in Iraq, Iran, Syria, Afghanistan, Eritrea or Somalia. Country of origin dummies are relative to Afghanistan (default category).

Table G.10: ATE estimates on vaccination intentions: stratum 2

	(T1 vs C)		(T2 vs C)		(T2 vs T1)	
	**Coeff.**	**(s.e.)**	**Coeff.**	**(s.e.)**	**Coeff.**	**(s.e.)**
Treatment (dummy)	0.002	(0.045)	0.115**	(0.046)	0.025	(0.036)
Mother's age (years)	-0.003	(0.005)	-0.008**	(0.004)	-0.002	(0.004)
Father's age (years)	-0.003	(0.005)	0.013**	(0.004)	-0.001	(0.004)
Child order (for mother)	1.587	(452.66)	1.018	(371.07)	1.174	(295.985)
HH disposable income (th. SEK)	0.016	(0.018)	0.012	(0.019)	0.021	(0.018)
MMR vaccine doses received	0.153**	(0.062)	0.068	(0.062)	-0.017	(0.296)
Swedish father (dummy)	0.048	(0.057)	0.031	(0.053)	-0.011	(0.042)
Female child	0.008	(0.044)	0.007	(0.041)	0.037	(0.036)
HH capital income (th. SEK)	-0.039	(0.03)	-0.013	(0.033)	-0.022	(0.084)
Married mother (dummy)	0.053	(0.048)	-0.032	(0.04)	0.033	(0.039)
Mother has medical educ. (dummy)	-0.039	(0.183)	-0.569	(15.513)	0.107**	(0.048)
Mother n. of children	0.009	(0.023)	0.043**	(0.021)	-0.003	(0.022)
Mother has numeric educ. (dummy)	0.02	(0.065)	0.01	(0.054)	0.043	(0.044)
Parent has medical occupation (dummy)	0.005	(0.061)	-0.075	(0.081)	0.001	(0.052)
Parent has research occupation (dummy)	0.053	(0.075)	0.04	(0.058)	0.057	(12.094)
Mother has scientific educ. (dummy)	0.053	(0.109)	0.177	(2.381)	-0.037	(0.143)
HH welfare transfers (SEK)	0.05	(0.067)	0.037	(0.061)	0.017	(0.045)

Notes: *** p< 0.01, ** p< 0.05, * p< 0.10. Results are estimated on the sample of respondents to the first survey: they can be interpreted as an ATE effect for this specific subpopulation. Stratum 2 comprises mothers with at most 3 years of high school: this corresponds to Swedish *högstadiet* (grades 7-9), the last compulsory grades under Swedish law.

Table G.11: ATE estimates on vaccination intentions: stratum 3

	(T1 vs C)		(T2 vs C)		(T2 vs T1)	
	**Coeff.**	**(s.e.)**	**Coeff.**	**(s.e.)**	**Coeff.**	**(s.e.)**
Treatment (dummy)	-0.021	(0.033)	0.022	(0.032)	0.029	(0.028)
Mother's age (years)	0.006	(0.049)	0.006	(0.004)	0.005	(0.005)
Father's age (years)	-0.001	(0.019)	-0.004	(0.004)	0.001	(0.003)
Child order (for mother)	0.002	(581.338)	0.9	(407.386)	1.017	(389.325)
HH disposable income (th. SEK)	0.013	(0.075)	0.011	(0.013)	0.006	(0.014)
MMR vaccine doses received	0.042	(0.675)	0.088	(0.056)	0.088**	(0.044)
Swedish father (dummy)	0	(0.044)	-0.05*	(0.03)	0.015	(0.044)
Female child	0.001	(0.032)	-0.01	(0.031)	0.008	(0.029)
HH capital income (th. SEK)	-0.054	(0.186)	0.009	(0.025)	-0.009	(0.017)
Married mother (dummy)	0.018	(0.034)	0.016	(0.033)	-0.013	(0.028)
Mother has medical educ. (dummy)	-0.568	(35.145)	-0.456	(102.72)	-0.621	(171.368)
Mother n. of children	0.002	(0.123)	-0.012	(0.021)	-0.02	(0.018)
Mother has numeric educ. (dummy)	-0.028	(0.05)	-0.008	(0.043)	0.004	(0.039)
Parent has medical occupation (dummy)	0.057	(0.036)	-0.029	(0.06)	0.036	(0.037)
Parent has research occupation (dummy)	0.016	(0.197)	0.044	(389.317)	-0.16	(203.272)
Mother has scientific educ. (dummy)	0.122	(2.142)	0.094	(9.049)	0.118	(18.38)
HH welfare transfers (SEK)	0.017	(0.597)	0.994	(138.301)	0.008	(0.046)

Notes: *** p< 0.01, ** p< 0.05, * p< 0.10. Results are estimated on the sample of respondents to the first survey: they can be interpreted as an ATE effect for this specific subpopulation. Mothers in stratum 3 completed high school (*gymnasium*, grades 10-12), which is not compulsory and comprises different tracks, including vocational ones.

Table G.12: ATE estimates on vaccination intentions: stratum 4

	(T1 vs C)		(T2 vs C)		(T2 vs T1)	
	**Coeff.**	**(s.e.)**	**Coeff.**	**(s.e.)**	**Coeff.**	**(s.e.)**
Treatment (dummy)	0.036	(0.036)	-0.010	(0.035)	-0.042	(0.032)
Mother's age (years)	0.001	(0.004)	-0.004	(0.005)	-0.001	(0.004)
Father's age (years)	-0.003	(0.004)	0.001	(0.006)	0.001	(0.003)
Child order (for mother)	0.975	(329.118)	0.997	(310.279)	0	(NA)
HH disposable income (th. SEK)	0.004	(0.012)	0.006	(0.01)	-0.003	(0.009)
MMR vaccine doses received	0.086	(0.093)	0.091	(0.078)	0.122**	(0.056)
Swedish father (dummy)	0.135**	(0.052)	0.067	(0.063)	0.058	(0.052)
Female child	0.02	(0.033)	0.016	(0.033)	-0.023	(0.03)
HH capital income (th. SEK)	0.065	(0.064)	-0.015	(0.018)	0	(0.014)
Married mother (dummy)	-0.038	(0.032)	-0.022	(0.036)	0.022	(0.032)
Mother has medical educ. (dummy)	-0.102	(0.151)	-0.191	(0.218)	0.078**	(0.038)
Mother n. of children	-0.011	(0.022)	0.023	(0.024)	0	(0.019)
Mother has numeric educ. (dummy)	-0.02	(0.047)	0.01	(0.041)	0.016	(0.037)
Parent has medical occupation (dummy)	0.076***	(0.016)	0.046	(0.049)	-0.017	(0.081)
Parent has research occupation (dummy)	-0.396*	(0.205)	0.063	(0.113)	-0.405	(0.252)
Mother has scientific educ. (dummy)	0	(0.06)	-0.003	(0.064)	-0.067	(0.079)
HH welfare transfers (SEK)	-0.069	(0.08)	-0.013	(0.079)	0.011	(0.057)

Notes: *** p< 0.01, ** p< 0.05, * p< 0.10. Results are estimated on the sample of respondents to the first survey: they can be interpreted as an ATE effect for this specific subpopulation. Mothers in stratum 4 have attended university, but not beyond an undergraduate degree.

Table G.13: ATE estimates on vaccination intentions: stratum 5

	(T1 vs C)		(T2 vs C)		(T2 vs T1)	
	**Coeff.**	**(s.e.)**	**Coeff.**	**(s.e.)**	**Coeff.**	**(s.e.)**
Treatment (dummy)	0.036	(0.036)	-0.010	(0.035)	-0.042	(0.032)
Mother's age (years)	0.001	(0.004)	-0.004	(0.005)	-0.001	(0.004)
Father's age (years)	-0.003	(0.004)	0.001	(0.006)	0.001	(0.003)
Child order (for mother)	0.975	(329.118)	0.997	(310.279)	0	(NA)
HH disposable income (th. SEK)	0.004	(0.012)	0.006	(0.01)	-0.003	(0.009)
MMR vaccine doses received	0.086	(0.093)	0.091	(0.078)	0.122**	(0.056)
Swedish father (dummy)	0.135**	(0.052)	0.067	(0.063)	0.058	(0.052)
Female child	0.02	(0.033)	0.016	(0.033)	-0.023	(0.03)
HH capital income (th. SEK)	0.065	(0.064)	-0.015	(0.018)	0	(0.014)
Married mother (dummy)	-0.038	(0.032)	-0.022	(0.036)	0.022	(0.032)
Mother has medical educ. (dummy)	-0.102	(0.151)	-0.191	(0.218)	0.078**	(0.038)
Mother n. of children	-0.011	(0.022)	0.023	(0.024)	0	(0.019)
Mother has numeric educ. (dummy)	-0.02	(0.047)	0.01	(0.041)	0.016	(0.037)
Parent has medical occupation (dummy)	0.076***	(0.016)	0.046	(0.049)	-0.017	(0.081)
Parent has research occupation (dummy)	-0.396*	(0.205)	0.063	(0.113)	-0.405	(0.252)
Mother has scientific educ. (dummy)	0	(0.06)	-0.003	(0.064)	-0.067	(0.079)
HH welfare transfers (SEK)	-0.069	(0.08)	-0.013	(0.079)	0.011	(0.057)

Notes: *** p< 0.01, ** p< 0.05, * p< 0.10. Results are estimated on the sample of respondents to the first survey: they can be interpreted as an ATE effect for this specific subpopulation. Mothers in stratum 4 have attended university beyond an undergraduate degree (postgraduate education).

H Robustness checks

H.1 Alternative specifications

Table H.14: ITT effect of information framing on actual vaccination uptake by LPM/OLS

**Stratum**	**Stratum definition**	**Uptake in control group**	**T1 *vs* C Emotional**	**T2 *vs* C Scientific**	**T2 *vs* T1**
1.	Immigrants	0.773	0.016	0.014	0.002
			(0.020)	(0.021)	(0.018)
**Swedish-born mothers**					
2. Educ-level-1	$\leq$ 3 yrs high school	0.786	0.033	0.049**	0.026
			(0.026)	(0.024)	(0.021)
3. Educ-level-2	Up to high school	0.887	-0.049**	0.009	0.046**
			(0.024)	(0.020)	(0.002)
4. Educ-level-3	Up to UG	0.905	-0.022	-0.028	-0.007
			(0.027)	(0.026)	(0.024)
5. Educ-level-4	Graduate	0.930	0.003	0.002	-0.009
			(0.021)	(0.021)	(0.019)
Total		0.867	-0.004	0.015	0.017
			(0.012)	(0.012)	(0.011)

Notes: *** p< 0.01, ** p< 0.05, * p< 0.10. Results are estimated on the entire sample of invited subjects: they can be interpreted as an ITT effect for the entire population of reference. Immigrants are mothers born in Iraq, Iran, Syria, Afghanistan, Eritrea or Somalia. Stratum 2 comprises mothers with at most 3 years of high school: this corresponds to Swedish *högstadiet* (grades 7-9), the last compulsory grades under Swedish law. Mothers in stratum 3 completed high school (*gymnasium*, grades 10-12), which is not compulsory and comprises different tracks, including vocational ones. The total effect in the last row refers to the totality of Swedish-born mothers.

Table H.15: ITT effect of information framing on actual vaccination uptake, structural LPM

Stratum	Stratum definition	Uptake in control group	T1 *vs* C Emotional	T2 *vs* C Scientific	T2 *vs* T1
1.	Immigrants	0.773	0.014	0.016	0.002
			(0.020)	(0.020)	(0.018)
**Swedish-born mothers**					
2. Educ-level-1	$\leq$ 3 yrs high school	0.786	0.030	0.054**	0.024
			(0.025)	(0.024)	(0.022)
3. Educ-level-2	Up to high school	0.887	-0.042*	0.005	0.047**
			(0.023)	(0.023)	(0.020)
4. Educ-level-3	Up to UG	0.905	-0.021	-0.027	-0.005
			(0.027)	(0.026)	(0.023)
5. Educ-level-4	Graduate	0.930	0.008	0.001	-0.007
			(0.021)	(0.021)	(0.018)

Notes: *** p< 0.01, ** p< 0.05, * p< 0.10. The model is a Linear Probability Model that includes both treatments as regressors: the "structural" interpretation derives from the fact that they can never be equal to 1 at the same time by design. Results are estimated on the entire sample of invited subjects: they can be interpreted as an ITT effect for the entire population of reference. Immigrants are mothers born in Iraq, Iran, Syria, Afghanistan, Eritrea or Somalia. Stratum 2 comprises mothers with at most 3 years of high school: this corresponds to Swedish *högstadiet* (grades 7-9), the last compulsory grades under Swedish law. Mothers in stratum 3 completed high school (*gymnasium*, grades 10-12), which is not compulsory and comprises different tracks, including vocational ones.

Table H.16: ATE of framing on intention to vaccinate among survey respondents, structural LPM

Stratum	Stratum definition	Uptake in control group	T1 *vs* C Emotional	T2 *vs* C Scientific	T2 *vs* T1
1.	Immigrants	0.830	-0.044<br>(0.055)	-0.033<br>(0.053)	0.011<br>(0.049)
**Swedish-born mothers**					
2. Educ-level-1	$\leq$ 3 yrs high school	0.862	0.020<br>(0.043)	0.084*<br>(0.045)	0.064<br>(0.040)
3. Educ-level-2	Up to high school	0.929	-0.022<br>(0.033)	0.006<br>(0.034)	0.028<br>(0.030)
4. Educ-level-3	Up to UG	0.931	0.024<br>(0.035)	-0.005<br>(0.036)	-0.030<br>(0.031)
5. Educ-level-4	Graduate	0.967	0.005<br>(0.024)	-0.028<br>(0.024)	-0.033<br>(0.021)

Notes: *** $p< 0.01$, ** $p< 0.05$, * $p< 0.10$. Results are estimated on the sample of survey respondents: within the corresponding population, they can be interpreted as the ATE of information framing on self-reported intention to vaccinate. Immigrants are mothers born in Iraq, Iran, Syria, Afghanistan, Eritrea or Somalia. Stratum 2 comprises mothers with at most 3 years of high school: this corresponds to Swedish *högstadiet* (grades 7-9), the last compulsory grades under Swedish law. Mothers in stratum 3 completed high school (*gymnasium*, grades 10-12), which is not compulsory and comprises different tracks, including vocational ones.

Table H.17: ATE of framing on intention to vaccinate among survey respondents, Likert Scale (1-7): LPM

Stratum	Stratum definition	Mean in control group	T1 *vs* C Emotional	T2 *vs* C Scientific	T2 *vs* T1
1.	Immigrants	6.075	-0.246	-0.022	0.044
			(0.223)	(0.190)	(0.186)
**Swedish-born mothers**					
2. Educ-level-1	≤ 3 yrs high school	6.074	0.168	0.421**	0.093
			(0.200)	(0.206)	(0.155)
3. Educ-level-2	Up to high school	6.473	-0.148	-0.077	0.082
			(0.155)	(0.151)	(0.138)
4. Educ-level-3	Up to UG	6.396	0.230	0.032	-0.175
			(0.153)	(0.148)	(0.134)
5. Educ-level-4	Graduate	6.631	0.063	-0.071	-0.180*
			(0.093)	(0.119)	(0.099)
Total (Swedish born)		6.413	0.054	-0.013	-0.051
			(0.068)	(0.071)	(0.061)

Notes: *** p< 0.01, ** p< 0.05, * p< 0.10. Intention to vaccinate is measured on a 1-7 Likert Scale, where 1 corresponds to no intention to vaccinate at all and 7 corresponds to willingness to vaccinate for sure. Results are estimated on the sample of survey respondents: within the corresponding population, they can be interpreted as the ATE of information framing on self-reported intention to vaccinate. Immigrants are mothers born in Iraq, Iran, Syria, Afghanistan, Eritrea or Somalia. Stratum 2 comprises mothers with at most 3 years of high school: this corresponds to Swedish *högstadiet* (grades 7-9), the last compulsory grades under Swedish law. Mothers in stratum 3 completed high school (*gymnasium*, grades 10-12), which is not compulsory and comprises different tracks, including vocational ones.

Table H.18: The effect of framing conditional on not responding to the first survey: LPM

Stratum	Stratum definition	Uptake in control group	T1 *vs* C Emotional	T2 *vs* C Scientific	T2 *vs* T1
1.	Immigrants	0.759	0.022	0.028	-0.010
			(0.034)	(0.034)	(0.029)
			N=619	N=635	N=784
**Swedish-born mothers**					
2. Educ-level-1	≤ 3 yrs high school	0.802	0.026	0.033	0.016
			(0.030)	(0.029)	(0.025)
			N=777	N=795	N=974
3. Educ-level-2	Up to high school	0.848	-0.054*	0.011	0.052*
			(0.030)	(0.029)	(0.028)
			N=557	N=597	N=704
4. Educ-level-3	Up to UG	0.863	-0.016	-0.017	-0.020
			(0.038)	(0.038)	(0.034)
			N=359	N=375	N=450
5. Educ-level-4	Graduate	0.922	0.044	0.019	-0.006
			(0.032)	(0.035)	(0.029)
			N=294	N=311	N=365

Notes: *** p< 0.01, ** p< 0.05, * p< 0.10. The outcome variable is the actual vaccination choice (binary indicator). Results are estimated by LPM/OLS on the subsample of mothers who did not answer our first survey. Immigrants are mothers born in Iraq, Iran, Syria, Afghanistan, Eritrea or Somalia. Stratum 2 comprises mothers with at most 3 years of high school: this corresponds to Swedish *högstadiet* (grades 7-9), the last compulsory grades under Swedish law. Mothers in stratum 3 completed high school (*gymnasium*, grades 10-12), which is not compulsory and comprises different tracks, including vocational ones.

Table H.19: The effect of framing conditional on responding to the first survey: LPM

**Stratum**	**Stratum definition**	**Uptake in control group**	**T1 *vs* C Emotional**	**T2 *vs* C Scientific**	**T2 *vs* T1**
1.	Immigrants	0.916	-0.066	-0.009	0.047
			(0.086)	(0.072)	(0.057)
			N=137	N=128	N=165
**Swedish-born mothers**					
2. Educ-level-1	$\leq$ 3 yrs high school	0.875	0.051	0.163***	0.091**
			(0.051)	(0.048)	(0.039)
			N=232	N=215	N=259
3. Educ-level-2	Up to high school	0.928	-0.037	0.016	0.048
			(0.036)	(0.031)	(0.029)
			N=315	N=281	N=372
4. Educ-level-3	Up to UG	0.916	-0.024	-0.024	0.013
			(0.039)	(0.036)	(0.035)
			N=269	N=249	N=316
5. Educ-level-4	Graduate	0.940	-0.033	-0.016	-0.010
			(0.029)	(0.028)	(0.026)
			N=335	N=321	N=412

Notes: *** $p < 0.01$, ** $p < 0.05$, * $p < 0.10$. The outcome variable is the actual vaccination choice (binary indicator). Results are estimated by LPM/OLS on the subsample of mothers who answered our first survey. Immigrants are mothers born in Iraq, Iran, Syria, Afghanistan, Eritrea or Somalia. Stratum 2 comprises mothers with at most 3 years of high school: this corresponds to Swedish *högstadiet* (grades 7-9), the last compulsory grades under Swedish law. Mothers in stratum 3 completed high school (*gymnasium*, grades 10-12), which is not compulsory and comprises different tracks, including vocational ones.

H.2 Removing and adding covariates

Table H.20: Removing covariates: main Logit specification

Specification	T1 vs C	s.e.	T2 vs C	s.e.	T2 vs T1	s.e.
**Stratum 1: Immigrants**						
No controls	0.02	(0.021)	0.011	(0.021)	-0.009	(0.019)
Demographic controls	0.017	(0.02)	0.013	(0.02)	0.004	(0.017)
Preferred specification	0.016	(0.02)	0.013	(0.02)	0.002	(0.017)
N	1572		1587		1937	
**Stratum 2: $\leq$ 3 yrs high school**						
No controls	0.035	(0.026)	0.047*	(0.026)	0.012	(0.022)
Demographic controls	0.034	(0.025)	0.054**	(0.024)	0.026	(0.021)
Preferred specification	0.037	(0.025)	0.057**	(0.024)	0.029	(0.021)
N	1009		1010		1233	
**Stratum 3: Up to high school**						
No controls	-0.041*	(0.023)	0.009	(0.022)	0.05**	(0.02)
Demographic controls	-0.039*	(0.023)	0.007	(0.022)	0.042**	(0.02)
Preferred specification	-0.048**	(0.022)	0.004	(0.021)	0.041**	(0.02)
N	872		878		1076	
**Stratum 4: Up to undergraduate**						
No controls	-0.025	(0.025)	-0.029	(0.025)	-0.004	(0.024)
Demographic controls	-0.02	(0.026)	-0.022	(0.025)	-0.011	(0.023)
Preferred specification	-0.016	(0.026)	-0.021	(0.025)	-0.005	(0.023)
N	628		624		766	
**Stratum 5: Graduate**						
No controls	0.008	(0.022)	-0.004	(0.021)	-0.012	(0.018)
Demographic controls	0.012	(0.022)	0.005	(0.021)	-0.014	(0.018)
Preferred specification	0.003	(0.021)	0.005	(0.021)	-0.006	(0.018)
N	629		632		777	

Notes: *** p< 0.01, ** p< 0.05, * p< 0.10. The outcome variable is the actual vaccination choice (binary indicator). Results are estimated by logit on the full sample. Demographic controls include: school municipality dummies, maternal age, married status (dummy), number of children, child's gender, order of birth, and number of MMR doses received, and whether the father has Swedish citizenship (dummy). The preferred specification adds: dummies for maternal scientific, numerical or medical education, high school final grade for both parents (where available), total capital and disposable income and total government transfers in the previous year, whether any parent has a medical or research occupation. The pre-registered specification is the preferred one.

H.3 T vs C test

In compliance with the Pre-Analysis Plan, in this section we report the results from testing the (T *vs* C) hypothesis using the main specification. Namely, we test whether any treatment (T, equal to 1 if either T1 or T2 is 1) is more effective than the reminder alone (C).

Table H.21: T *vs* C

Stratum	Stratum definition	Uptake in control group	**T** *vs* **C** Any framed information
1.	Immigrants	0.759	0.014
			(0.018)
**Swedish-born mothers**			
2. Educ-level-1	$\leq$ 3 yrs high school	0.802	0.048**
			(0.023)
3. Educ-level-2	Up to high school	0.848	-0.019
			(0.019)
4. Educ-level-3	Up to UG	0.863	-0.018
			(0.023)
5. Educ-level-4	Graduate	0.922	0.007
			(0.019)

Notes: *** p< 0.01, ** p< 0.05, * p< 0.10. Results are estimated by Logit on the full sample, the outcome is actual vaccination against HPV. Immigrants are mothers born in Iraq, Iran, Syria, Afghanistan, Eritrea or Somalia. Stratum 2 comprises mothers with at most 3 years of high school: this corresponds to Swedish *högstadiet* (grades 7-9), the last compulsory grades under Swedish law. Mothers in stratum 3 completed high school (*gymnasium*, grades 10-12), which is not compulsory and comprises different tracks, including vocational ones.

H.4 Spillovers

In this section, we use our causal forest estimates to show that spillover effects between children in the same school are unlikely to drive the null results.

Our sample comprises 7616 children from 611 schools in 49 municipalities. Figure H.4 shows the distribution of the number of children in the same school. 6303 children (83% of the total) are from schools with 6 or more children included in this study. 4546 (60% of all children) are from schools attended by more than 10 children in the study. For privacy reasons, we do not know the total number of students in each school nor the class of each child and therefore cannot evaluate the likelihood that these children (or their parents) meet and exchange information. Since many of our estimates are not statistically significant, one might wonder whether this is driven by spillover effects. To exclude this possibility, we look at Conditional Intention-To-Treat effects (CITTs)

from our causal forest analysis, and we correlate them with the number of children in the school. Specifically, conditional on the number of children in the same school, we compute the mean CITT. If null results are driven by spillover effects, one would expect the mean CITT to tend to zero as the number of children increases, because children in the control group are more likely to communicate with children (or parents) in treatment groups. Figure H.5 and Figure H.6 show that is not the case, respectively for the effect of emotional Framing (T1) and scientific framing (T2). The CITT effects show no significant negative correlation with the number of children in the same school.[36] Note that while it is not shown for the sake of brevity, this result is robust to the use of CITT computed within strata.

Figure H.4: Distribution of the number of children in the same school

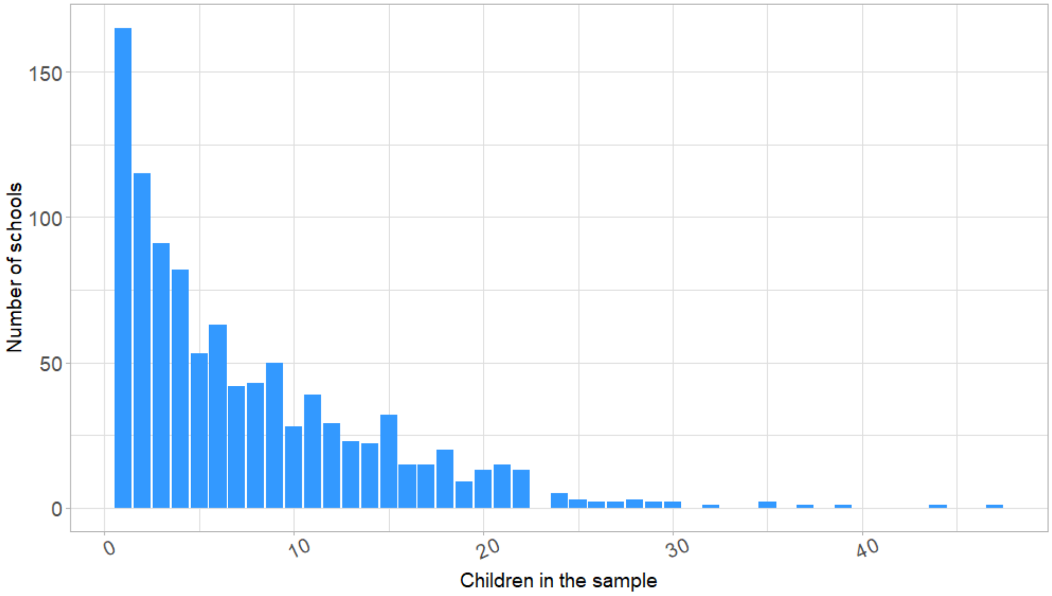

[36]The absence of correlation is maintained also by looking at individual CITT effects instead of computing the mean CITT for each number of children in school.

Figure H.5: Mean CITT (Emotional Framing - T1) by the number of children in the same school

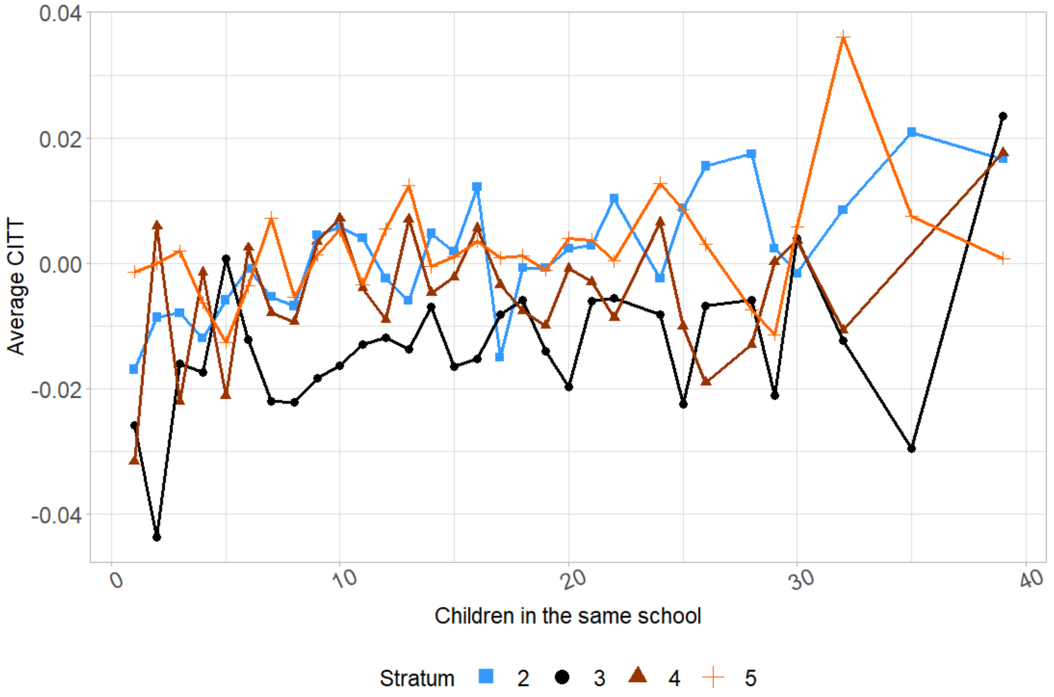

Notes: The figure shows, for each stratum, the average Conditional ITT Effect of emotional Framing (T1) on actual vaccinations by the number of children attending the same school. Namely, within each cell defined by the number of children attending the same school, we compute the average individual ITT effect from our causal forest analysis. We do not show standard errors to improve readability: the only significant effect is in stratum 3, as in the main analysis. If there were any spillover effects between the treatment groups and the control group, they should be more likely as the number of children in the same school increases.

Figure H.6: Mean CITT (Scientific Framing - T2) by the number of children in the same school

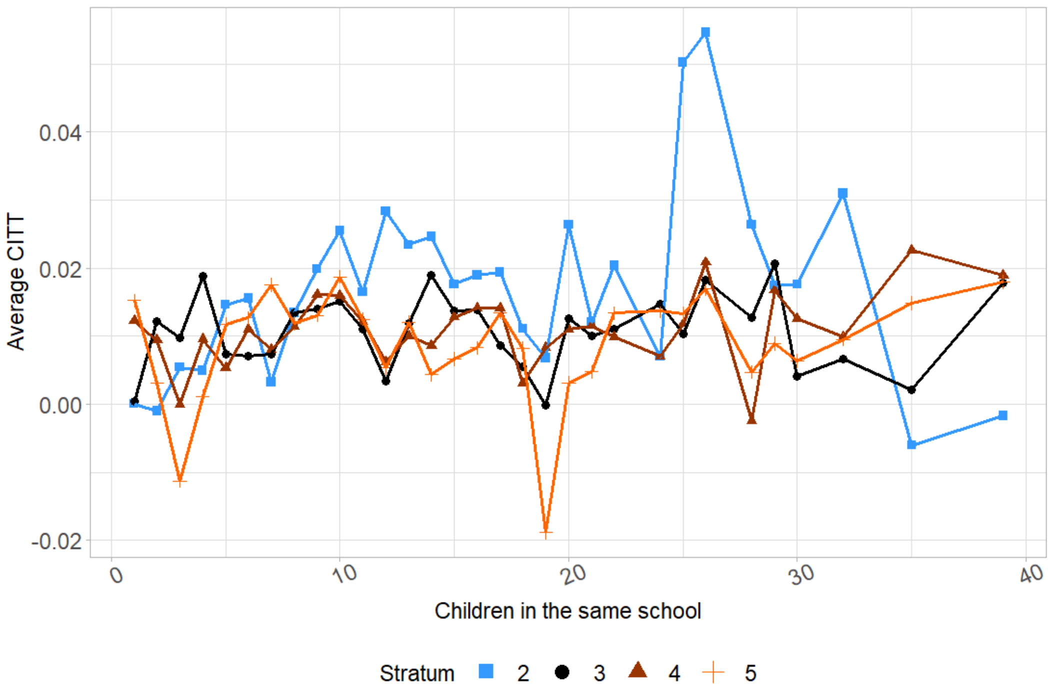

Notes: The figure shows, for each stratum, the average Conditional ITT Effect of scientific Framing (T2) on actual vaccinations by the number of children attending the same school. Namely, within each cell defined by the number of children attending the same school, we compute the average individual ITT effect from our causal forest analysis. We do not show standard errors to improve readability: the only significant effect is in stratum 2, as in the main analysis. If there were any spillover effects between the treatment groups and the control group, they should be more likely as the number of children in the same school increases.

I Interactions between our intervention and COVID-19: qualitative evidence

Our intervention concerns a childhood vaccine. It was carried out in the summer of 2021 when the volume of (dis)information on Covid-19 vaccines was high and possibly interacted with our intervention. We investigate this possibility, given the importance of pre-treatment exposure to information on vaccines for our treatment effects. To do so, we rely on the results by Eichengreen et al. (2021). Using data from 1973 to before Covid-19, the authors find that being exposed to a pandemic between ages 18 and 25 decreases trust in scientists and translates into lower uptake of vaccinations for children. The effects are driven by individuals with little or no scientific training. Moreover, it is not found among health professionals. Figure I.7 and Figure I.8 show the correlation between the individual causal effect (CITT) in strata 2 and 3 and maternal age. For emotional framing (T1), younger mothers experience significant negative effects that approach zero as age increases. Consistent with our main result, this is more pronounced in stratum 3, where the average effect is negative. The correlation is also positive for scientific framing (T2), although in this case, mothers in impressionable years (25-35) experience null or marginally negative effects.

For stratum 2, Figure I.9 shows the distribution of individual effects based on whether mothers below age 30 have a job in the healthcare industry.[37] As expected, the negative effects of emotional framing (T1) are more pronounced for mothers who do no work in healthcare, and the distribution for scientific framing (T2) is more concentrated around 0 for mothers with a healthcare job. Overall, we take these results as suggestive evidence that Covid-19 impacted our results in line with predictions from previous literature.

[37]We do not have a sufficient number of young mothers with a high school scientific major in stratum 2. In any case, stratum 2 mothers attended at most 3 years of high school and thus the major is unlikely to have a lasting impact.

Figure I.7: Effect of emotional framing (T1) by mother's age

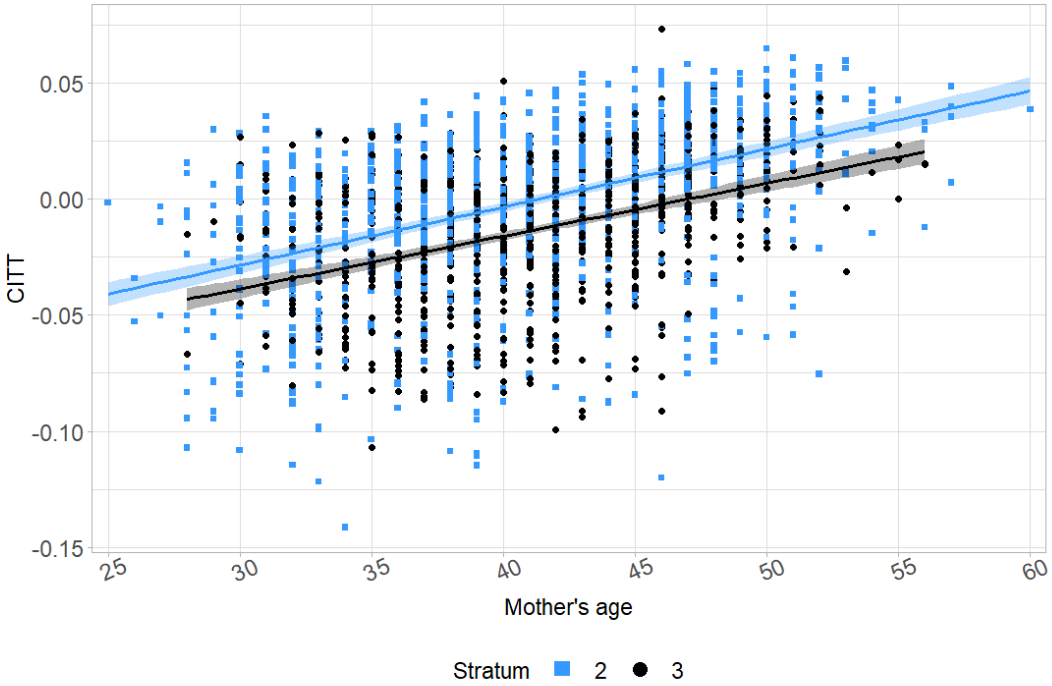

Notes: The figure shows, separately for stratum 2 and 3, the scatterplot of mother's age and the Conditional ITT effect of emotional framing (T1).

Figure I.8: Effect of scientific framing (T2) by mother's age

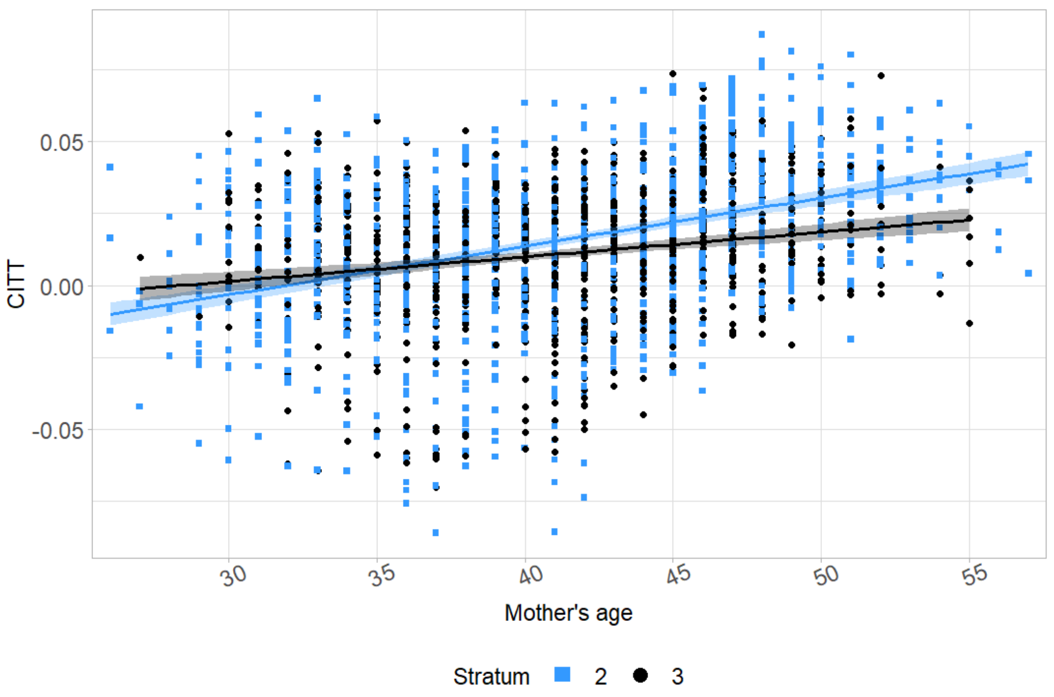

Notes: The figure shows, separately for stratum 2 and 3, the scatterplot of mother's age and the Conditional ITT effect of scientific framing (T2).

Figure I.9: CITT for young mothers in stratum 2 by healthcare occupation

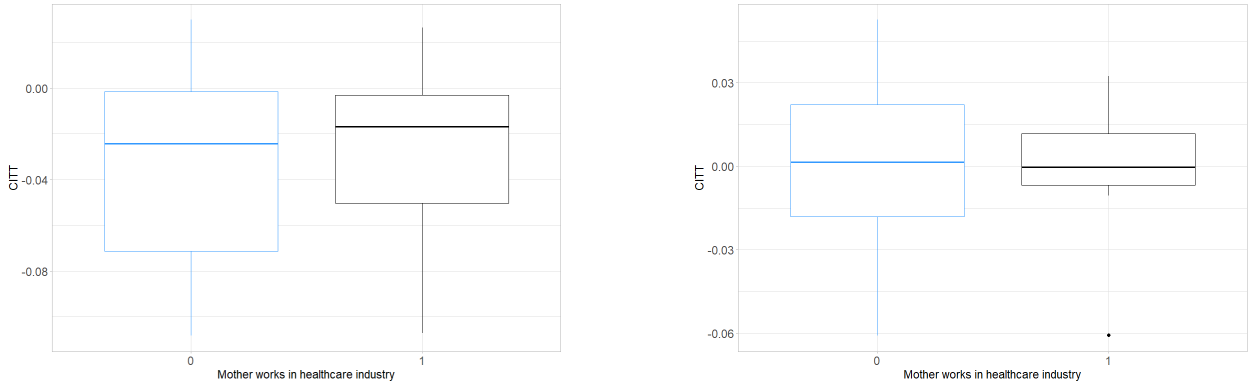

(A.) Emotional framing (T1) **(B.)** Scientific framing (T2)

Notes: The figure shows the distribution of the Conditional ITT of scientific framing (T2) for stratum 2 mothers below 30 years old, differentiating by whether they work in the healthcare industry. According to Eichengreen et al. (2021), individuals exposed to an epidemic between ages 18 and 25 who do not work in healthcare develop more negative views of vaccines.

J Information sources and intervention timeline

Table J.22: Sources of information by treatment group and topic

	**Sources**			
**Information leaflet**	**Public Health Agency of Sweden**	**International Agency for Cancer Research (2019)**	**European Medical Agency (2015)**	**Nätverket mot gynekologisk cancer**
C	History of the Swedish vaccination program			
T1	Introductory HPV information	•Swedish cervical cancer statistics<br>•Introductory HPV information	Vaccine safety and efficacy	Swedish cancer survivors' testimonies
T2	Introductory HPV information	•Swedish cervical cancer statistics<br>•Introductory HPV information	•Vaccine safety and efficacy<br>•Vaccine clinical trial statistical information	

Table J.23: Content of envelopes by date, and printed/online version

Date of dispatch	Swedish-born mothers		Immigrant mothers	
	Printed	Online	Printed	Online
16th June	• Invitation letter[1]<br>•Leaflet[1]<br>•First survey[1]	•Invitation letter[1]<br>•Leaflet[1]<br>•First survey[1]	•Invitation letter[2]<br>•Leaflet[2]<br>•First survey[2]	•Invitation letter[2]<br>•Leaflet[2]<br>•First survey[1]
5th July	Reminder letter[1]	•Invitation letter[1]<br>•Leaflet[1]<br>•First survey[1]	Reminder letter[2]	•Invitation letter[2]<br>•Leaflet[2]<br>•First survey[1]
11th August	•Reminder letter[1]<br>•First survey[1]	•Invitation letter[1]<br>•Leaflet[1]<br>•First survey[1]	•Reminder letter[2]<br>•First survey[2]	•Invitation letter[2]<br>•Leaflet[2]<br>•First survey[1]
20th August	Reminder letter[1]	•Invitation letter[1]<br>•Leaflet[1]<br>•First survey[1]	Reminder letter[2]	•Invitation letter[2]<br>•Leaflet[2]<br>•First survey[1]
**September - October**	**HPV vaccination is offered**			
15th November	•Invitation letter[1]<br>•Endline survey[1]	•Invitation letter[1]<br>•Endline survey[1]	•Invitation letter[2]<br>•Endline survey[2]	•Invitation letter[2]<br>• Endline survey[2]

Notes: 1: Swedish only
2: Swedish and Arabic/Dari depending on origin country.

All invitation letters remind the presence of other languages options online (English included).

K Misconceptions on vaccines

In our first survey, we ask parents how much they agree with two common misconceptions about vaccines typically spread by disinformation. We measure agreement with the following statements on a 5-point Likert scale, and then construct a misconception index taking the mean:

1. Vaccines weaken and overload the immune system;
2. Vaccines can cause the disease against which they protect.

Figure K.10 shows the distribution of the misconception index by respondents' previous exposure to HPV information. Both in the overall sample and within strata, the distribution is more concentrated at lower misconception levels when respondents have already heard of HPV, although differences are not statistically significant.

This result complements other pieces of evidence that diminishing returns to information might be in place. Still, there are two caveats. First, misconceptions are elicited after exposure to treatment: while previous exposure to HPV information is balanced and treatment should not drive the different distributions, it might have improved beliefs for all respondents. Second, we are only observing these distributions for respondents who engaged with our leaflets. Both caveats imply that the true unobservable differences – once considering non-respondents and netting out treatment effects – might actually be more pronounced.

Since misconceptions are measured after treatment, we then test by OLS whether our treatments affect vaccination choices through a change in beliefs about vaccines. Our intervention addresses misconceptions about vaccines by shifting the attention away from the low probability and low severity of the HPV vaccine towards the more likely and far more severe consequences of catching a preventable HPV-induced cancer. The results in Table K.24 show that there is no reduction in misconceptions. On the contrary, highly educated mothers worsen their beliefs when exposed to emotional framing (T1). It should be underlined that these results are estimated on respondents to the first survey – stratum 5 mothers who raised their uptake after emotional framing were instead non-respondents. The effects of informational framing in strata 2 and 3 do not pass through a change in misconceptions about vaccines.

Figure K.10: Misconceptions by previous exposure to HPV information

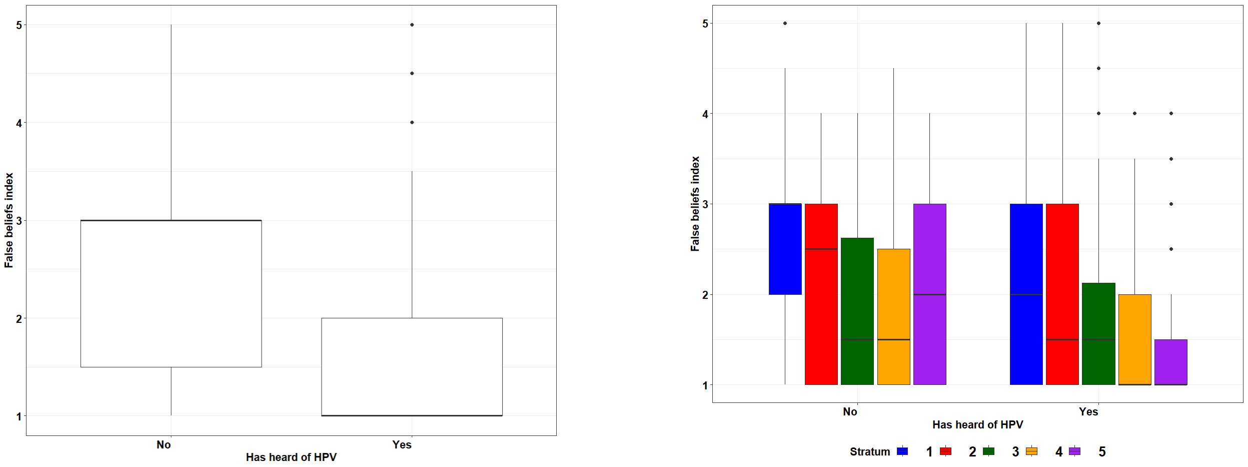

(A.) Overall respondents sample

(B.) By stratum

Table K.24: ATE of information framing on vaccine misconceptions

Stratum	Stratum definition	Value in the control group	T1 *vs* C Emotional	T2 *vs* C Scientific	T2 *vs* T1
1.	Immigrants	2.495	0.140	0.005	-0.126
			(0.150)	(0.155)	(0.137)
**Swedish-born mothers**					
2. Educ-level-1	$\leq$ 3 yrs high school	1.780	0.088	0.054	-0.031
			(0.141)	(0.147)	(0.130)
3. Educ-level-2	Up to high school	1.66	0.108	0.063	-0.027
			(0.107)	(0.118)	(0.100)
4. Educ-level-3	Up to UG	1.58	-0.098	0.008	0.042
			(0.108)	(0.111)	(0.095)
5. Educ-level-4	Graduate	1.24	0.165**	0.071	-0.059
			(0.080)	(0.075)	(0.071)

Notes: *** p< 0.01, ** p< 0.05, * p< 0.10. The outcome variable is the mean of the degree to which subjects agree with two common misconceptions about vaccines (5-point Likert scale). Results are estimated on the subsample of survey respondents for whom the outcome is observed: they can be interpreted as an ATE effect for this subpopulation. Immigrants are mothers born in Iraq, Iran, Syria, Afghanistan, Eritrea or Somalia. Stratum 2 comprises mothers with at most 3 years of high school: this corresponds to Swedish *högstadiet* (grades 7-9), the last compulsory grades under Swedish law. Mothers in stratum 3 completed high school (*gymnasium*, grades 10-12), which is not compulsory and comprises different tracks, including vocational ones.

L Power calculations

This section presents the Minimum Detectable Effects (MDE) in each stratum (1-5) and for each comparison: (T1 vs C), (T2 vs C), and (T2 vs T1), for both the primary outcome of actual vaccination records and the secondary outcome of self-reported willingness to vaccinate.

First, within the reference group – C for treatment vs placebo and T1 for treatments against each other – we compute the variance of the residual after regressing the actual HPV vaccination record on all the covariates used in the main analysis.

Then, we compute the MDE with the following formula:

$$MDE = (z_{1-\beta} + z_{\alpha}) \times \frac{1}{\sqrt{P(1-P)}} \times \frac{\sigma_{\text{res}}}{\sqrt{N}}$$

where $z_{1-\beta}$ is the critical value when $1-\beta = 0.8$, z_{α} is the critical value for a two-tailed test when $\alpha = 0.05$, $P = \frac{N_t}{N_t+N_c}$ is the proportion of treated units, σ_{res} is the standard deviation of the residual from the regressions described above, and $N = N_t + N_c$ is the total sample size.

The sample sizes and P change depending on whether we consider the analysis to be performed by reduced or structural form equations. These are respectively:[38]

$$Y_i = \alpha + \tau T_i + \boldsymbol{X_i'\beta} + \eta_m + \varepsilon_i \qquad \text{(Red.)}$$

$$Y_i = \alpha + \tau_1 T1_i + \tau_2 T2_i + \boldsymbol{X_i'\beta} + \eta_m + \varepsilon_i \qquad \text{(Struct.)}$$

[38] In the case of (T2 vs T1), the structural test consists of testing the hypothesis $H_0 : \tau_2 \neq \tau_1$.

Table L.25: Minimum Detectable Effects in the primary analysis (treatments *vs* placebo)

Stratum	σ_{res}	N_C	N_{T1}	N_{T2}	MDE T1 vs C Reduced form	MDE T2 vs C Reduced form	MDE T1 vs C Structural form	MDE T2 vs C Structural form
1	0.133	611	961	976	0.053	0.053	0.042	0.042
2	0.127	393	616	617	0.064	0.064	0.051	0.051
3	0.079	337	535	541	0.055	0.055	0.043	0.043
4	0.072	243	385	381	0.062	0.062	0.049	0.049
5	0.049	242	387	390	0.051	0.051	0.040	0.040

Notes: MDE for power $1-\beta = 0.8$ and $\alpha = 0.05$ in two-tailed comparisons. σ_{res} is the variance of residuals obtained from regressing the outcome of interest (vaccination records) on all covariates used in the main analysis, within the control group. "Reduced" and "Structural" refer to the equations used to estimate causal effects in the main analysis, Equations (Red.) and (Struct.).

Table L.26: Minimum Detectable Effects in the primary analysis (T2 *vs* T1)

Stratum	σ_{res}	N_C	N_{T1}	N_{T2}	MDE T2 vs T1 Reduced form	MDE T2 vs T1 Structural form
1	0.132	611	961	976	0.046	0.041
2	0.120	393	616	617	0.055	0.050
3	0.107	337	535	541	0.056	0.050
4	0.094	243	385	381	0.062	0.056
5	0.049	242	387	390	0.044	0.040

Notes: MDE for power $1-\beta = 0.8$ and $\alpha = 0.05$ in two-tailed comparisons. σ_{res} is the variance of residuals obtained from regressing the outcome of interest (vaccination records) on all covariates used in the main analysis, within the T1 group. "Reduced" and "Structural" refer to the equations used to estimate causal effects in the main analysis, Equations (Red.) and (Struct.).

Table L.27: Minimum Detectable Effects in the secondary analysis (treatments *vs* placebo)

Stratum	σ_{res}	N_C	N_{T1}	N_{T2}	MDE T1 vs C Reduced form	MDE T2 vs C Reduced form	MDE T1 vs C Structural form	MDE T2 vs C Structural form
1	0.133	106	148	162	0.130	0.128	0.105	0.101
2	0.127	94	138	121	0.133	0.137	0.109	0.115
3	0.079	112	203	169	0.093	0.096	0.073	0.078
4	0.072	101	168	148	0.095	0.097	0.075	0.079
5	0.049	122	213	199	0.070	0.071	0.055	0.056

Notes: MDE for power $1-\beta = 0.8$ and $\alpha = 0.05$ in two-tailed comparisons. σ_{res} is the variance of residuals obtained from regressing the outcome of interest (vaccination records) on all covariates used in the main analysis, within the control group. "Reduced" and "Structural" refer to the equations used to estimate causal effects in the main analysis, Equations (Red.) and (Struct.).

Table L.28: Minimum Detectable Effects in the secondary analysis (T2 *vs* T1)

Stratum	σ_{res}	N_C	N_{T1}	N_{T2}	MDE T2 vs T1 Reduced form	MDE T2 vs T1 Structural form
1	0.132	106	148	162	0.116	0.101
2	0.120	94	138	121	0.121	0.111
3	0.107	112	203	169	0.095	0.091
4	0.094	101	168	148	0.097	0.090
5	0.049	122	213	199	0.061	0.056

Notes: MDE for power $1 - \beta = 0.8$ and $\alpha = 0.05$ in two-tailed comparisons. σ_{res} is the variance of residuals obtained from regressing the outcome of interest (vaccination records) on all covariates used in the main analysis, within the T1 group. "Reduced" and "Structural" refer to the equations used to estimate causal effects in the main analysis, Equations (Red.) and (Struct.).

M Missing data and multiple imputation

We follow Little and Rubin (2019) and use multiple imputation to deal with missingness in baseline covariates from population registers, included in the model as per the pre-registration. Importantly, we do not need to impute the outcome variable, which would be problematic: the actual vaccination record has no missingness in our sample. We adopt CART models as a imputation method, as they are suitable for all covariates, and pick $m = 5$ (the minimum number suggested by Little and Rubin (2019) to achieve consistency). The imputation is implemented using package *mice* in R, whereas aggregation is performed manually. We estimate the regressions in each of the 5 imputed datasets, compute the estimate as their mean, and use Rubin's formula to aggregate the variance to compute standard errors, namely:

$$\text{s.e.}_{\text{pooled}} = \sqrt{W + \left(1 + \frac{1}{m}\right) B}$$

where:

- $W = \frac{1}{m} \sum_{i=1}^{m} U_i$ is the within-imputation variance, with U_i being the variance of the parameter estimate from the i-th imputation.
- $B = \frac{1}{m-1} \sum_{i=1}^{m} (\bar{\theta} - \theta_i)^2$ is the between-imputation variance, where θ_i is the parameter estimate from the i-th imputation, and $\bar{\theta} = \frac{1}{m} \sum_{i=1}^{m} \theta_i$ is the mean of the estimates across imputations.
- m is the number of imputations, in our case 5

Figure M.11-Figure M.15 describe, for each stratum, the percentage of missing observations for each baseline covariate used in our main specification, plus some extra covariates on fathers. Note that in stratum 2 mothers have only completed 3 years of high school: high school grade is thus missing for the majority of them, and when it is observed it refers to the first 3 years.

Figure M.11: Stratum 1: missingness in baseline covariates

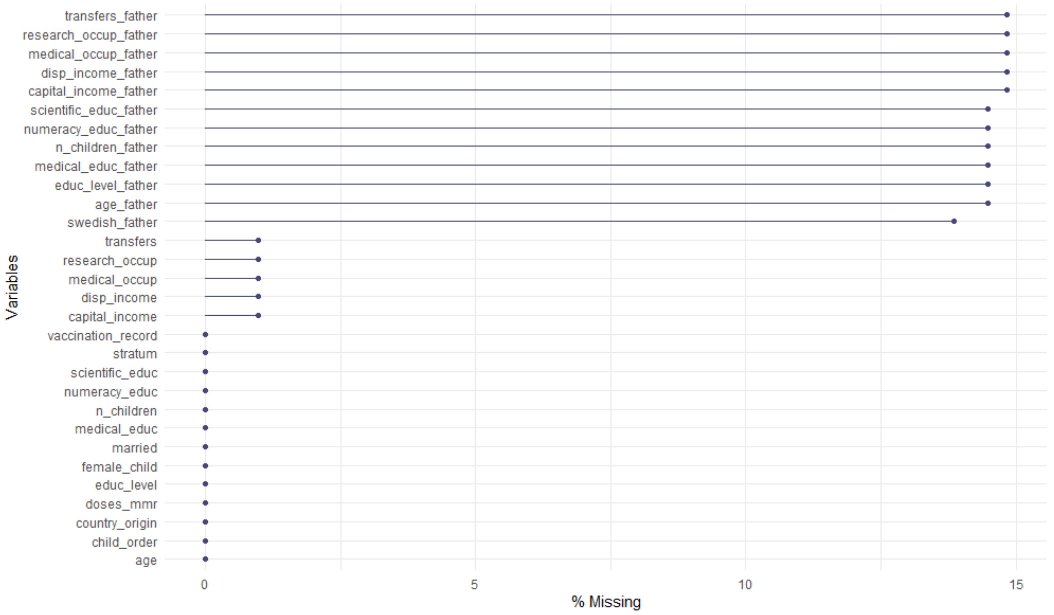

Notes: Stratum 1 contains immigrant mothers from selected origin countries.

Figure M.12: Stratum 2: missingness in baseline covariates

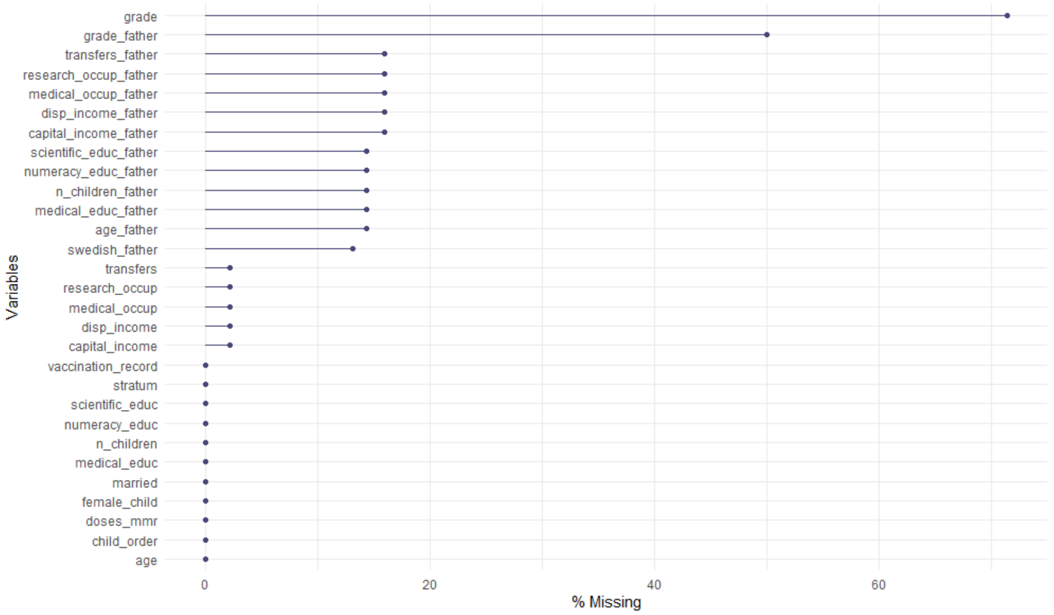

Notes: Stratum 2 contains mothers with compulsory education (3 years of high school). Grade indicates their grade at the end of those 3 years.

Figure M.13: Stratum 3: missingness in baseline covariates

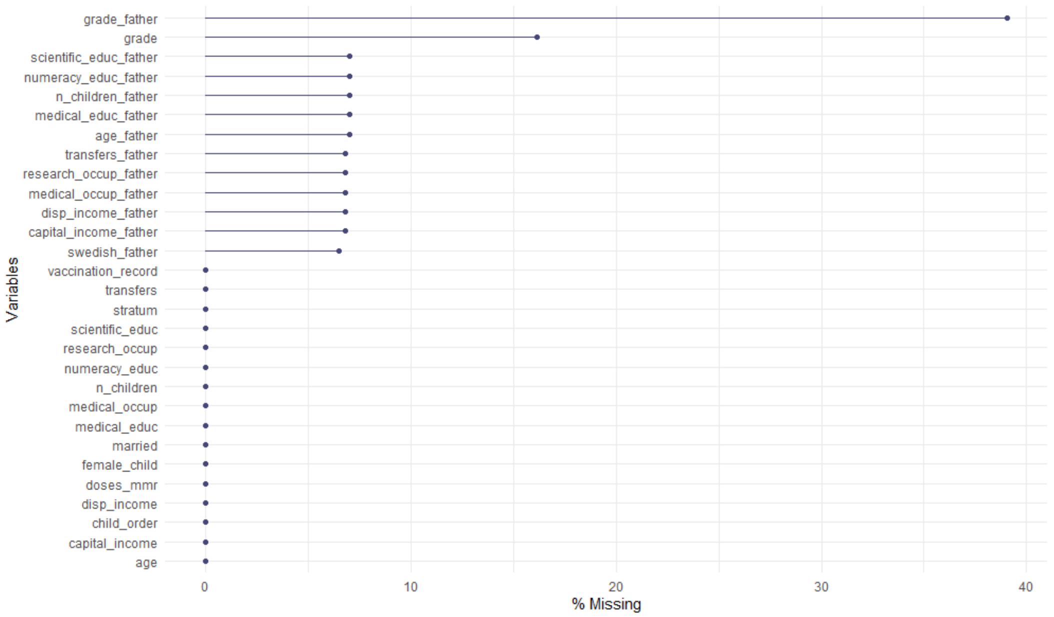

Notes: Stratum 3 contains mothers with a high school degree. "Grade" indicates their final high school degree.

Figure M.14: Stratum 4: missingness in baseline covariates

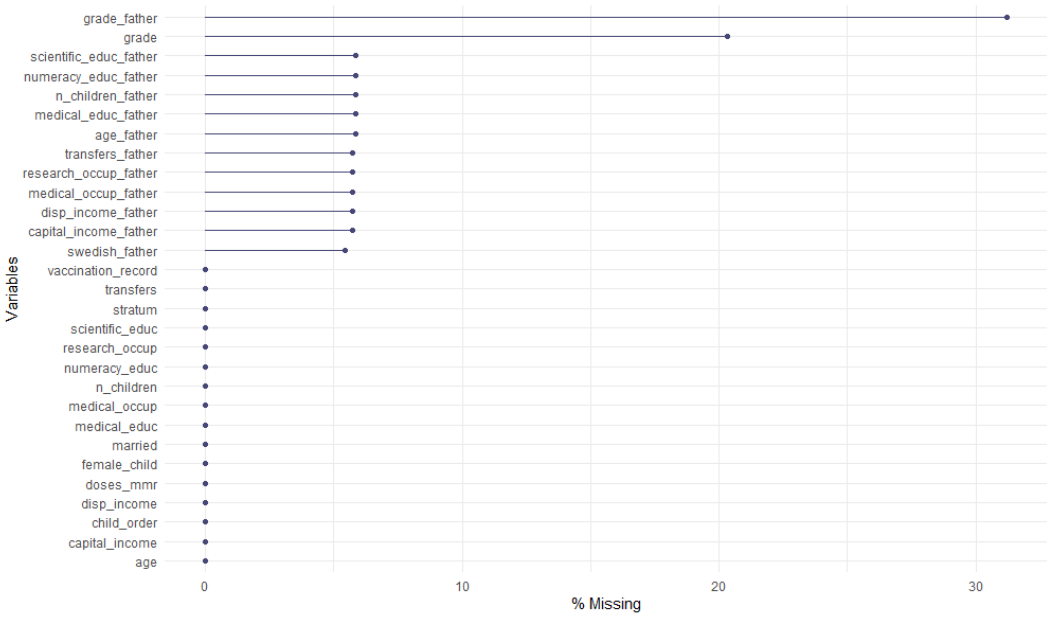

Notes: Stratum 4 contains mothers with some university education, up to a bachelor degree. "Grade" indicates their final high school degree.

Figure M.15: Stratum 5: missingness in baseline covariates

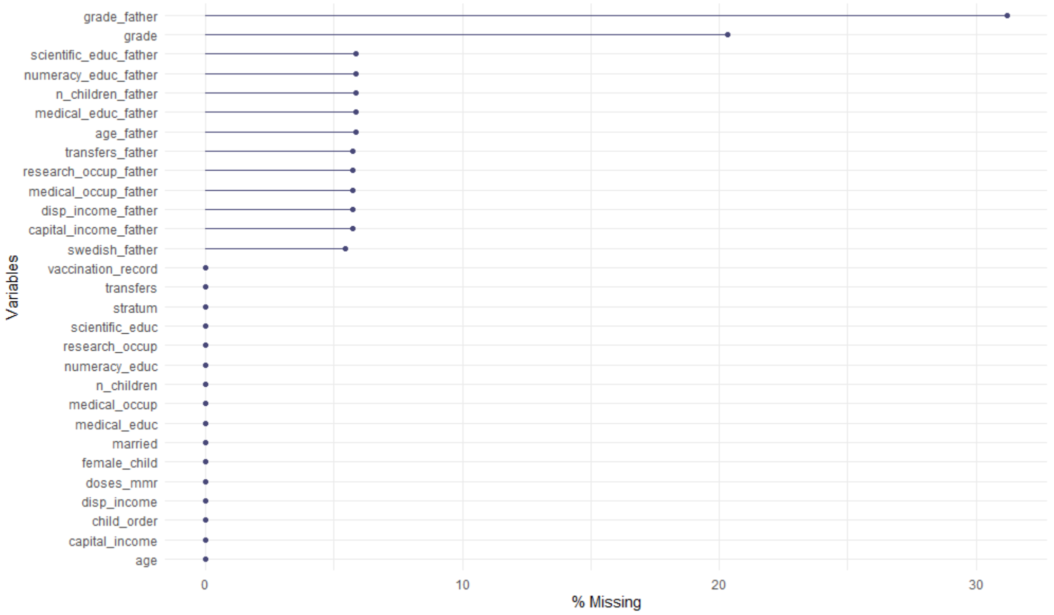

Notes: Stratum 5 contains mothers with some graduate education. "Grade" indicates their final high school degree.

N The characteristics of survey respondents

Figure N.16 shows the distribution of answers to the question "What percentage of the leaflet did you read?", for respondent mothers, differentiating between immigrant and Swedish-born mothers. Both distribution have an average answer of "between 70 and 80%". However, the median and mode answer is "between 90 and 100%", and as discussed in Section 7, answers are unlikely to be driven by social desirability bias.

Figure N.16: Percentage read among respondents

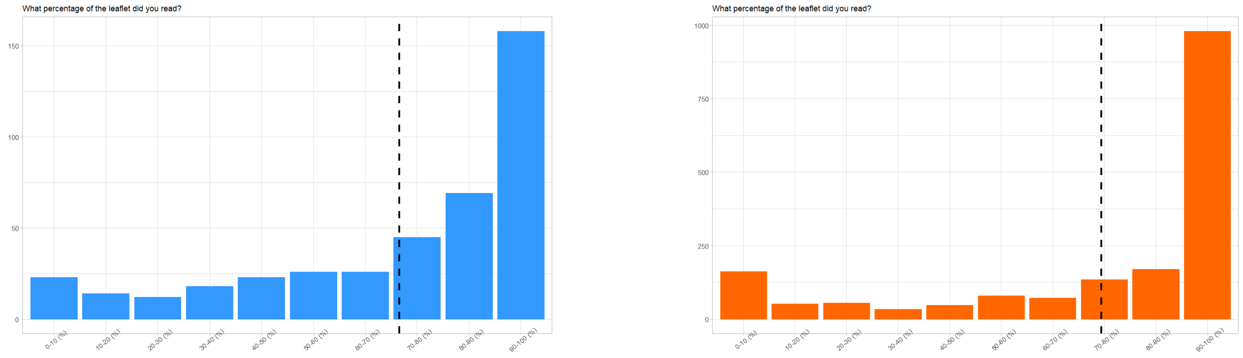

(A.) Immigrant mothers **(B.)** Swedish-born mothers

Notes: The figure shows, separately for immigrant and Swedish-born mothers, the distribution of answers to the question "What percentage of the leaflet did you read?", contained in the first survey ($N = 2204$). The vertical dashed line indicates the mean, equal to 7.66 for immigrant mothers and 7.94 for Swedish-born mothers.

Table N.29 and Table N.30 show ITT estimates for survey respondents and non-respondents, respectively. Figure N.17 and Figure N.18 present the 90% C.I. graphically to ease readability, displaying first T1 and then T2.

In the following tables, we compare baseline covariates between:

- Subjects who never replied to any survey *vs* Subjects who replied at least to the first survey;
- Subjects who replied to the first survey *vs* Subjects who replied to both the first and the second survey.

Both comparisons are presented twice: first irrespective of treatment status, and then separately by treatment status (C, T1 or T2).

Table N.29: The effect of framing conditional on responding to the first survey

Stratum	Stratum definition	Uptake in control group	T1 *vs* C Emotional	T2 *vs* C Scientific	T2 *vs* T1
1.	Immigrants	0.916	-0.041	-0.021	0.036
			(0.034)	(0.023)	(0.035)
			N=137	N=128	N=165
**Swedish-born mothers**					
2. Educ-level-1	$\leq$ 3 yrs high school	0.875	0.006	0.161***	0.094**
			(0.047)	(0.045)	(0.033)
			N=232	N=215	N=259
3. Educ-level-2	Up to high school	0.928	-0.030	0.000	0.045*
			(0.034)	(0.029)	(0.027)
			N=315	N=281	N=372
4. Educ-level-3	Up to UG	0.916	-0.025	-0.022	0.015
			(0.036)	(0.033)	(0.033)
			N=269	N=249	N=316
5. Educ-level-4	Graduate	0.940	-0.036	-0.012	0.003
			(0.026)	(0.026)	(0.025)
			N=335	N=321	N=412

Notes: *p<0.1; **p<0.05; ***p<0.01. The outcome variable is the actual vaccination choice (binary indicator). Results are estimated by Logit on the subsample of mothers who read our leaflet attentively, proxied by having answered our first survey. Immigrants are mothers born in Iraq, Iran, Syria, Afghanistan, Eritrea or Somalia. Stratum 2 comprises mothers with at most 3 years of high school: this corresponds to Swedish *högstadiet* (grades 7-9), the last compulsory grades under Swedish law. Mothers in stratum 3 completed high school (*gymnasium*, grades 10-12), which is not compulsory and comprises different tracks, including vocational ones.

Table N.30: The effect of framing conditional on not responding to the first survey

Stratum	Stratum definition	Uptake in control group	T1 *vs* C Emotional	T2 *vs* C Scientific	T2 *vs* T1
1.	Immigrants	0.759	0.027	0.017	-0.007
			(0.023)	(0.023)	(0.020)
			N=619	N=635	N=784
**Swedish-born mothers**					
2. Educ-level-1	$\leq$ 3 yrs high school	0.802	0.029	0.044	0.020
			(0.029)	(0.028)	(0.024)
			N=777	N=795	N=974
3. Educ-level-2	Up to high school	0.848	-0.057*	0.002	0.047*
			(0.030)	(0.028)	(0.027)
			N=557	N=597	N=704
4. Educ-level-3	Up to UG	0.863	-0.000	-0.008	-0.018
			(0.037)	(0.036)	(0.032)
			N=359	N=375	N=450
5. Educ-level-4	Graduate	0.922	0.065*	0.020	-0.013
			(0.035)	(0.032)	(0.028)
			N=294	N=311	N=365

Notes: *p<0.1; **p<0.05; ***p<0.01. The outcome variable is the actual vaccination choice (binary indicator). Results are estimated by Logit on the subsample of mothers who did not answer our first survey. Immigrants are mothers born in Iraq, Iran, Syria, Afghanistan, Eritrea or Somalia. Stratum 2 comprises mothers with at most 3 years of high school: this corresponds to Swedish *högstadiet* (grades 7-9), the last compulsory grades under Swedish law. Mothers in stratum 3 completed high school (*gymnasium*, grades 10-12), which is not compulsory and comprises different tracks, including vocational ones.

Figure N.17: Effect of emotional framing (T1) for respondents and non-respondents

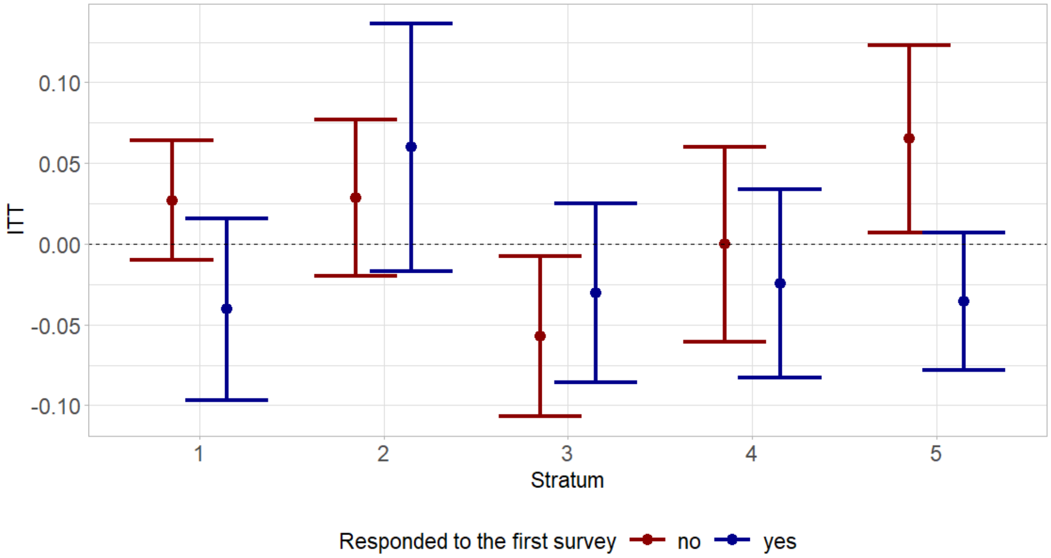

The figure shows estimates and 90% C.I. of the ITT effect of emotional framing, estimated separately among respondents and non-respondents.

Figure N.18: Effect of scientific framing (T2) for respondents and non-respondents

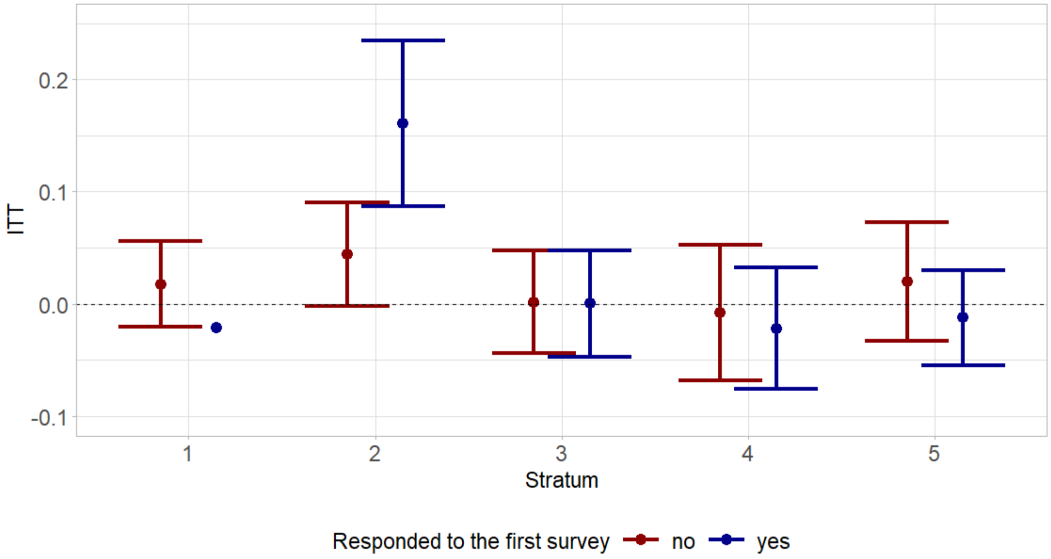

The figure shows estimates and 90% C.I. of the ITT effect of scientific framing, estimated separately among respondents and non-respondents. s.e. for stratum 1 respondents are not displayed because they are very large, due to the low response rate.

Table N.31: Respondents compared to never-respondents

Covariate	ASD	Non-respondents	Respondents
**Mother's characteristics**			
Age	0.254***	40.51	42.49
Married (dummy)	0.058**	0.63	0.669
Scientific educ. (dummy)	0.019	0.21	0.221
Medical educ. (dummy)	0.038**	0.147	0.128
Numerical educ. (dummy)	0.118***	0.15	0.214
Capital income (Thousands SEK)	0.014	341.682	25.115
Disposable income (Thousands SEK)	0.027*	3305.918	3753.373
Job in research	0.046**	0.003	0.007
Job in healthcare	0.108***	0.18	0.126
**Father's characteristics**			
Father is a researcher	0.038*	0.004	0.009
Father works in healthcare	0.083***	0.055	0.031
**Child's characteristics**			
Female (dummy)	0.026	0.484	0.502
Birth order	0.059***	1.035	1.021
Second dose of MMR (dummy)	0.121***	0.95	0.981

Notes: *** p< 0.01, ** p< 0.05, * p< 0.10 on two-tailed tests of difference in means. Respondents and non-respondents are used to estimate different estimands between primary and secondary outcomes. Responding to the survey is also used as a proxy of attentiveness to explain the mechanism behind ITT effects. Denoting respondents to the first survey as R and non-repondents as NR, The Absolute Standardized Difference (ASD) for variable X is computed as: $ASD \equiv \frac{|\bar{X}_R - \bar{X}_{NR}|}{\sqrt{Var_R(X)+Var_{NR}(X)}}$.

Table N.32: Respondents to first survey compared to respondents to both surveys

**Covariate**	**ASD**	**Replied once (R)**	**Replied twice (RR)**
**Mother's characteristics**			
Age	0.017	42.45	42.574
Married (dummy)	0.033	0.662	0.685
Scientific educ. (dummy)	0.003	0.22	0.222
Medical educ. (dummy)	0.056*	0.136	0.11
Numerical educ. (dummy)	0.016	0.211	0.221
Capital income (Thousands SEK)	0.025	8.748	59.853
Disposable income (Thousands SEK)	0.065**	3679.519	3910.129
Job in research	0.039	0.006	0.011
Job in healthcare	0.104**	0.141	0.093
**Father's characteristics**			
Father is a researcher	0.013	0.009	0.008
Father works in healthcare	0.02	0.032	0.028
**Child's characteristics**			
Female (dummy)	0.008	0.504	0.498
Birth order	0.04	1.023	1.015
Second dose of MMR (dummy)	0.03	0.979	0.985
**Treatment status**			
T1	0.039	0.385	0.412
T2	0.033	0.353	0.375
**Answers to the first survey**			
Has heard of HPV before treatment	0.122***	0.835	0.894
% leaflet read	0.141***	76.696	82.634

Notes: *** p< 0.01, ** p< 0.05, * p< 0.10 on two-tailed tests of difference in means. Denoting responding to the second survey as R and respondents only to the first survey as NR, The Absolute Standardized Difference (ASD) for variable X is computed as: $ASD \equiv \frac{|\bar{X}_R - \bar{X}_{NR}|}{\sqrt{Var_R(X) + Var_{NR}(X)}}$.

Table N.33: Respondents to compared to never respondents, by treatment

	Control (C)			Emotional framing (T1)			Scientific framing (T2)		
**Covariate**	**ASD**	**Never replied**	**Replied**	**ASD**	**Never replied**	**Replied**	**ASD**	**Never replied**	**Replied**
**Mother's characteristics**									:
Age	0.257***	40.575	42.59	0.276***	40.381	42.526	0.23***	40.593	42.379
Married	0.063*	0.625	0.667	0.048	0.635	0.667	0.063**	0.632	0.674
Scientific education	0.001	0.198	0.199	0.002	0.217	0.216	0.052*	0.21	0.241
Medical education	0.088**	0.142	0.102	0.021	0.149	0.139	0.026	0.148	0.135
Numerical education	0.117**	0.153	0.217	0.114***	0.153	0.216	0.121***	0.146	0.211
Capital income	0.02	-15.524	17.735	0.023	843.331	-4.24	0.003	78.674	62.312
Disposable income	0.215***	3079.229	3664.339	0.005	3634.074	3766.232	0.133***	3129.774	3803.973
Research occupation	0.098**	0.002	0.014	0.023	0.004	0.006	0.024	0.002	0.004
Medical occupation	0.154***	0.184	0.108	0.117***	0.182	0.122	0.067**	0.176	0.142
**Father's characteristics**									:
Age	0.121**	44.342	45.404	0.101***	44.262	45.152	0.1***	44.216	45.099
Married	0.047	0.633	0.665	0.036	0.645	0.669	0.042	0.642	0.67
Scientific education	0.059	0.313	0.353	0.074**	0.329	0.38	0.057*	0.315	0.353
Medical education	0.019	0.033	0.038	0.003	0.043	0.044	0.018	0.035	0.04
Numerical education	0.174***	0.185	0.289	0.184***	0.2	0.312	0.18***	0.196	0.305
Capital income	0.021	2268.592	857.763	0.052	303.392	966.207	0.001	541.168	527.097
Disposable income	0.01	5969.48	5312.478	0.099**	4226.021	5257.613	0.043	4512.62	5019.658
Research occupation	0.065	0.003	0.01	0.048	0.005	0.011	0.006	0.005	0.005
Medical occupation	0.14***	0.061	0.022	0.087**	0.055	0.03	0.041	0.05	0.038
**Child's characteristics**									:
Female	0.033	0.469	0.492	0.022	0.501	0.486	0.072**	0.475	0.526
MMR 1st dose	0.032	0.907	0.92	0.062**	0.897	0.923	0.124***	0.878	0.929
MMR 2nd dose	0.124***	0.948	0.98	0.146***	0.95	0.986	0.092**	0.951	0.975
Order of birth	0.047	1.03	1.02	0.06**	1.027	1.015	0.063**	1.045	1.027

Notes: *** $p< 0.01$, ** $p< 0.05$, * $p< 0.10$ on two-tailed tests of difference in means. Denoting responding to at least the first survey as R and non-repondents units NR, The Absolute Standardized Difference (ASD) for variable X is computed as: $ASD \equiv \frac{|\bar{X}_R - \bar{X}_{NR}|}{\sqrt{Var_R(X)+Var_{NR}(X)}}$.

Table N.34: Respondents to first survey compared to respondents to both surveys, by treatment

	Control (C)			Emotional framing (T1)			Scientific framing (T2)		
**Covariate**	**ASD**	**Replied once**	**Replied twice**	**ASD**	**Replied once**	**Replied twice**	**ASD**	**Replied once**	**Replied twice**
**Mother's characteristics**									:
Age	0.032	42.656	42.417	0.043	42.42	42.735	0.024	42.319	42.498
Married	0.041	0.675	0.647	0.003	0.666	0.668	0.11**	0.65	0.722
Scientific education	0.023	0.196	0.209	0.038	0.223	0.201	0.029	0.235	0.253
Medical education	0.1	0.113	0.072	0.036	0.144	0.127	0.065	0.145	0.114
Numerical education	0.09	0.231	0.18	0.02	0.212	0.224	0.076	0.196	0.241
Capital income	0.106	-29.295	140.554	0.05	-37.184	61.28	0.032	87.092	12.853
Disposable income	0.124*	3566.237	3920.532	0.072	3690.572	3916.705	0.037	3750.047	3911.604
Research occupation	0.059	0.011	0.022	0.016	0.006	0.007	0.061	0.002	0.008
Medical education	0.118*	0.121	0.072	0.11**	0.139	0.09	0.098*	0.157	0.11
**Father's characteristics**									:
Age	0.054	45.526	45.086	0.013	45.118	45.22	0.089	44.855	45.588
Married	0.022	0.669	0.655	0.014	0.666	0.675	0.119**	0.644	0.722
Scientific education	0.015	0.355	0.345	0.047	0.39	0.358	0.095*	0.331	0.396
Medical education	0.048	0.041	0.029	0.014	0.045	0.041	0.029	0.037	0.045
Numerical education	0.002	0.289	0.288	0.037	0.304	0.328	0.105*	0.282	0.351
Capital income	0.026	773.675	1077.36	0.057	1252.994	395.843	0.02	572.81	435.857
Disposable income	0.051	5123.609	5805.712	0.048	5442.537	4889.836	0.005	5034.456	4990.122
Research occupation	0.041	0.008	0.014	0.131**	0.017	0	0.086	0.002	0.012
Medical occupation	0.002	0.022	0.022	0.022	0.028	0.034	0.079	0.045	0.024
**Child's characteristics**									:
Female	0.034	0.499	0.475	0.017	0.49	0.478	0.019	0.521	0.535
MMR 1st dose	0.055	0.915	0.935	0.011	0.921	0.925	0.027	0.933	0.922
MMR 2nd dose	0.099	0.975	0.993	0.011	0.987	0.985	0.029	0.973	0.98
Order of birth	0.04	1.022	1.014	0	1.015	1.015	0.075	1.033	1.016
**First survey answers**									:
Attention	0.15**	0.948	0.986	0.059	0.936	0.955	0.078	0.935	0.959
Believes vaccines cause the disease	0.217**	1.854	1.532	0.202***	1.867	1.567	0.158**	1.859	1.616
Believes vaccines weaken the immune system	0.179**	1.747	1.504	0.17***	1.859	1.604	0.165**	1.822	1.58
Heard of HPV before the study	0.107	0.824	0.878	0.088*	0.85	0.892	0.163**	0.828	0.906
% of leaflet read	0.04	7.705	7.878	0.161**	7.679	8.358	0.177**	7.634	8.371
Searched vaccine info from unreliable sources	0.126*	0.229	0.158	0.028	0.182	0.198	0.04	0.215	0.192
Trusts health authorities	0.236***	4.237	4.54	0.116**	4.353	4.5	0.123**	4.333	4.494

Notes: *** p< 0.01, ** p< 0.05, * p< 0.10 on two-tailed tests of difference in means. Denoting responding to the second survey as RR and respondents only to the first survey as R, The Absolute Standardized Difference (ASD) for variable X is computed as: $ASD \equiv \frac{|\bar{X}_{RR} - \bar{X}_R|}{\sqrt{Var_{RR}(X) + Var_R(X)}}$.

O Intention to vaccinate and actual vaccination

The following graphs summarize, for each stratum, the mismatch between the intention to vaccinate (measured in the first survey right after treatment) and actual vaccination status from the administrative records.

Figure O.19: Stratum 1: intention to vaccinate and vaccination

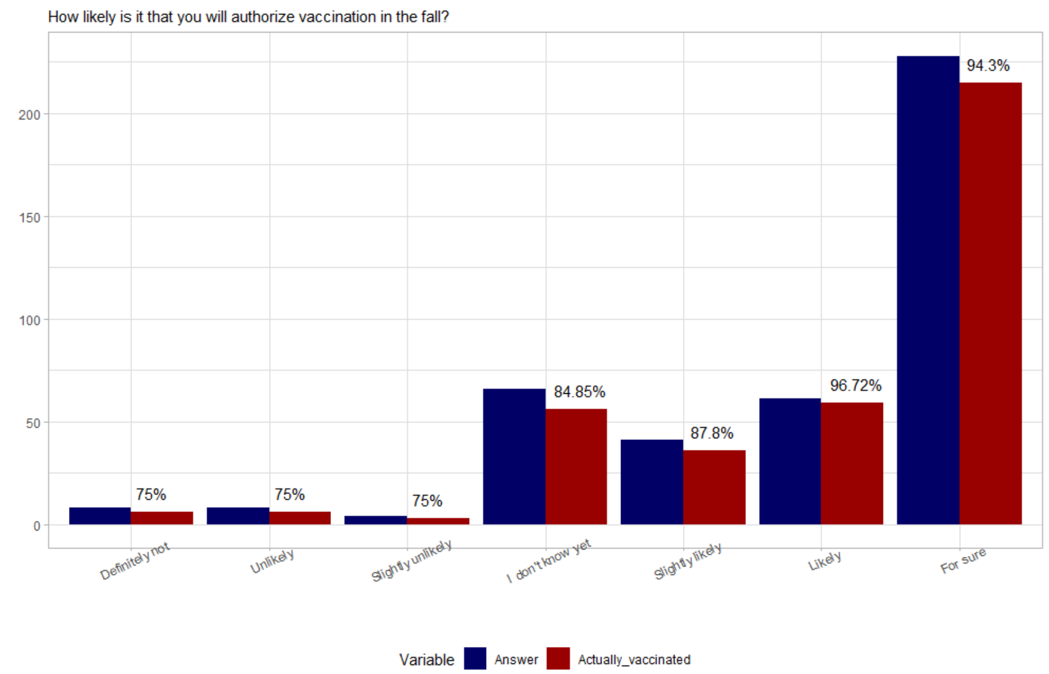

Notes: for each possible answer to the question "How likely are you to authorize vaccination in the fall?" the blue bar indicates the number of respondents, and the red bar the number of respondents who actually vaccinated in the fall. The percentage value of actually vaccinated respondents is reported on top of the red bar. Data are restricted to stratum 1 (immigrant mothers).

Figure O.20: Stratum 2: intention to vaccinate and vaccination

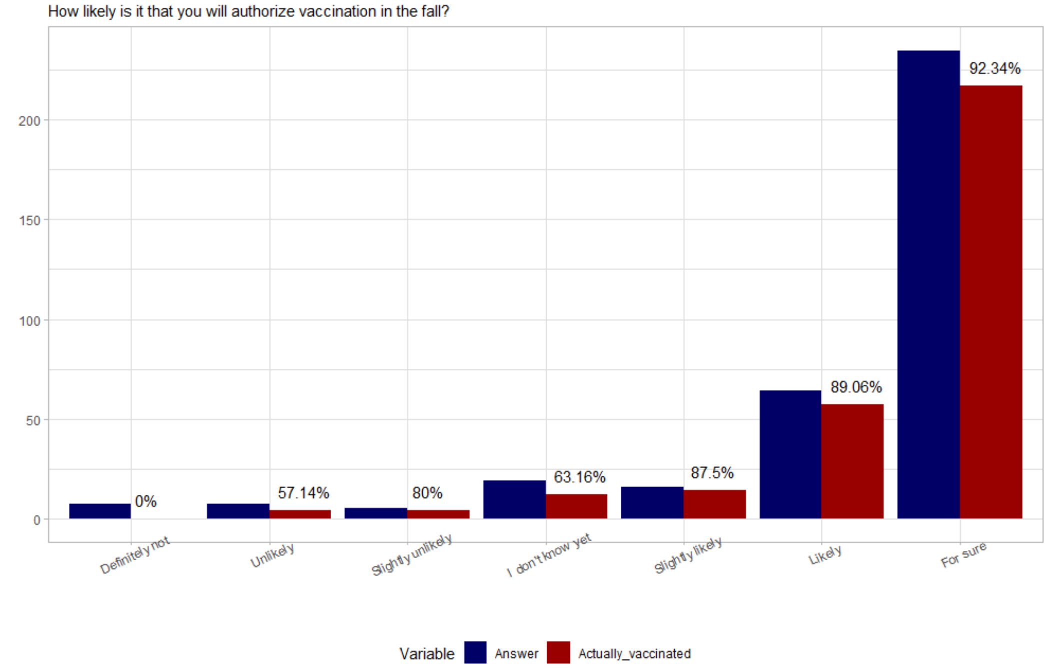

Notes: for each possible answer to the question "How likely are you to authorize vaccination in the fall?" the blue bar indicates the number of respondents, and the red bar the number of respondents who actually vaccinated in the fall. The percentage value of actually vaccinated respondents is reported on top of the red bar. Data are restricted to stratum 2 (mothers with compulsory schooling, equivalent to 3 years of high school).

Figure O.21: Stratum 3: intention to vaccinate and vaccination

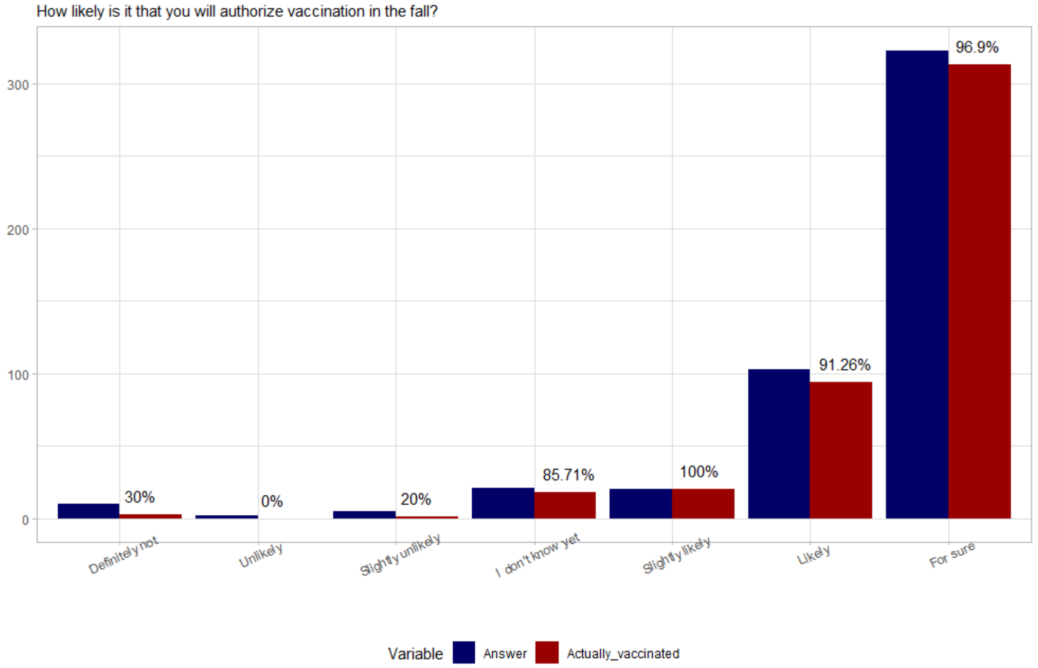

Notes: for each possible answer to the question "How likely are you to authorize vaccination in the fall?" the blue bar indicates the number of respondents, and the red bar the number of respondents who actually vaccinated in the fall. The percentage value of actually vaccinated respondents is reported on top of the red bar. Data are restricted to stratum 3 (mothers with a high school degree).

Figure O.22: Stratum 4: intention to vaccinate and vaccination

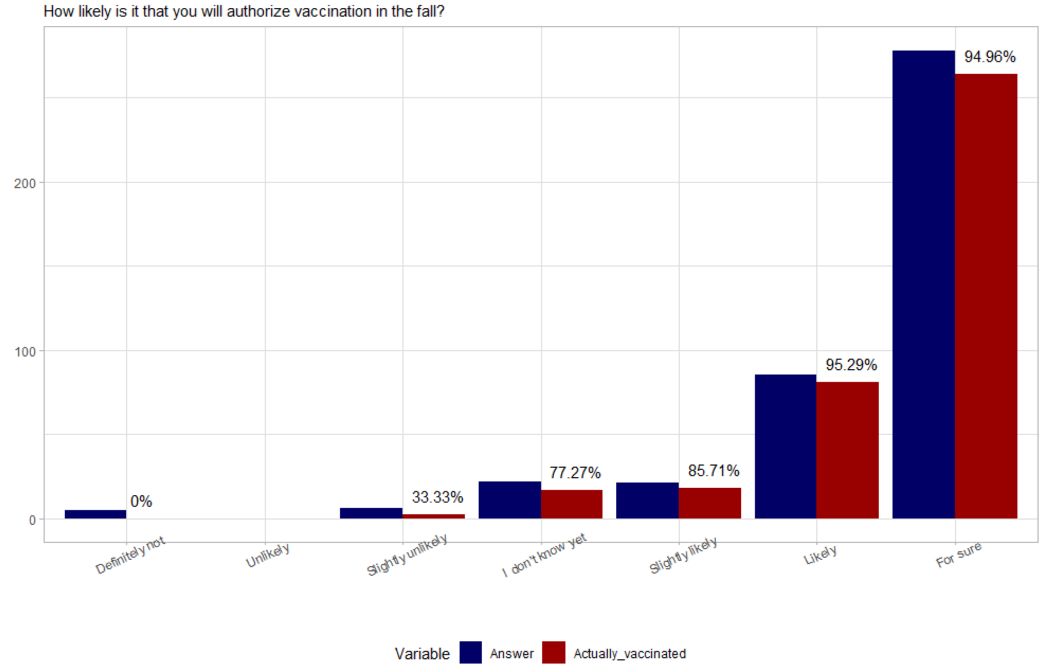

Notes: for each possible answer to the question "How likely are you to authorize vaccination in the fall?" the blue bar indicates the number of respondents, and the red bar the number of respondents who actually vaccinated in the fall. The percentage value of actually vaccinated respondents is reported on top of the red bar. Data are restricted to stratum 4 (mothers with some university education).

Figure O.23: Stratum 5: intention to vaccinate and vaccination

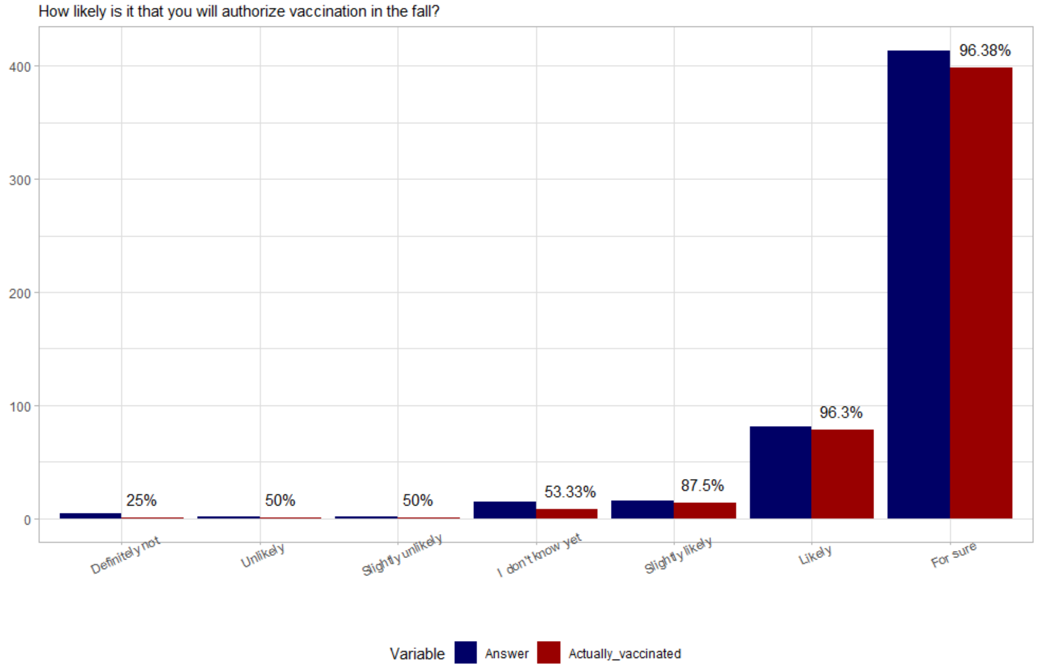

Notes: for each possible answer to the question "How likely are you to authorize vaccination in the fall?" the blue bar indicates the number of respondents, and the red bar the number of respondents who actually vaccinated in the fall. The percentage value of actually vaccinated respondents is reported on top of the red bar. Data are restricted to stratum 5 (mothers with more than a bachelor degree).

P Heterogeneity by gender

HPV is mostly known for causing cervical cancer, which only affects women. However, the incidence of HPV-induced head-neck and penile cancers is rising, and men can be asymptomatic vectors of the virus. In 2020 Sweden enlarged access to the free HPV vaccine to boys, and several European countries are planning to follow. The first HPV vaccine was launched in the US in 2006. Our mothers gave birth in 2009: unless they actively sought information about it, they were probably not targeted by informational campaigns to get vaccinated themselves. Moreover, since boys were just included in the program, it is likely that boys' mothers have been exposed to less information on the HPV vaccine, absent effects from previous children.[39] Table P.35 shows the average answer to survey questions on HPV information by child gender, and tests for significant differences in mean. Indeed, boys' mothers have received less information on HPV, have heard less often about HPV, and have read a slightly higher percentage of the leaflet. Therefore, we look at heterogeneity by gender as an additional indicator of previous information which does not depend only on self-reported survey answers.

We investigate heterogeneity by including an interaction term:

$$Y_i = \alpha + \tau_1 T_i + \gamma \text{Female}_i + \tau_2 (T \times Female)_i + \boldsymbol{X_i'\beta} + \eta_m + \varepsilon_i \qquad (1)$$

where Female_i is a binary indicator of the child's gender, τ_1 identifies the effect on males and $\tau_1 + \tau_2$ identifies the effect on females.

The following figures show the ITT effects of emotional (T1) and scientific (T2) framing on actual vaccination uptake, tested against the placebo group:

In line with our hypothesis, the significant effects in our main analysis are driven by mothers of boys.

[39] 97% of our sample is composed of children without siblings.

Table P.35: Exposure to HPV information in male and female children mothers

Indicator	Females mean	Males mean	Means difference (Males-Females)
% of leaflet read	77.60	79.85	2.25*
Has received HPV information passively before treatment	0.848	0.784	-0.064***
Has actively searched HPV information before treatment	0.51	0.475	-0.035
Intends to actively search HPV information after treatment	0.986	0.984	-0.002
Has actively searched HPV information before treatment from untrustworthy sources	0.199	0.202	0.004
Has heard about HPV before treatment	0.863	0.83	-0.034**

[-1.2ex]

Notes: *** p< 0.01, ** p< 0.05, * p< 0.10, obtained with two-tailed T-tests for difference in means from two samples with unequal variance. The survey questions from which these indicators have been obtained can be found in Section F in the Appendix.

Figure P.24: ITT effect of emotional framing (T1) on actual vaccination by child's gender

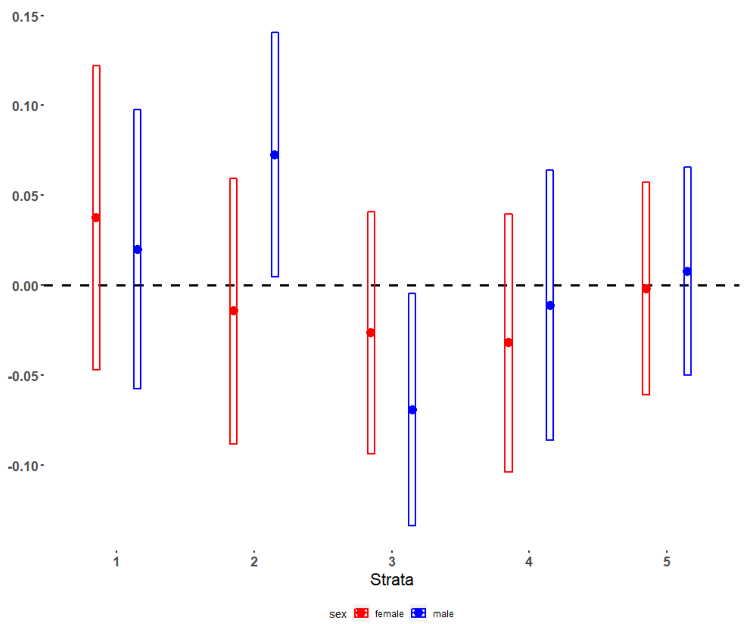

Figure P.25: ITT effect of scientific framing (T2) on actual vaccination by child's gender

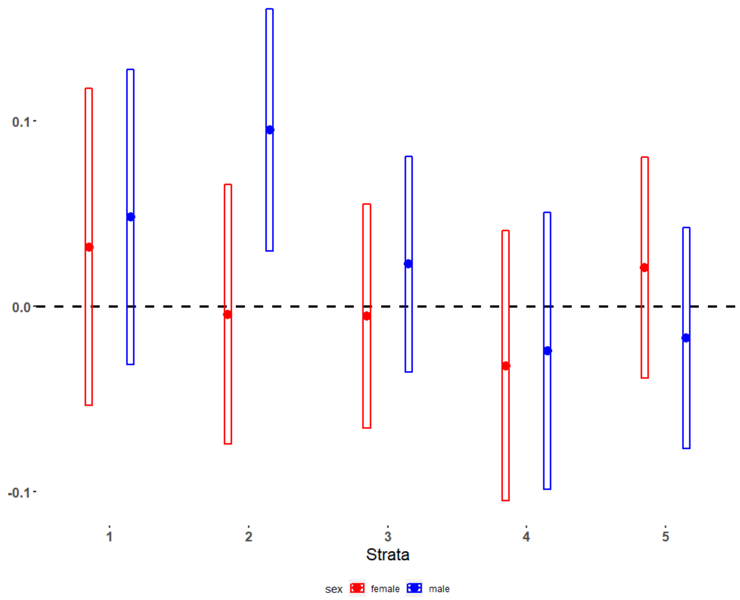

Q Causal forests: extra results

Q.1 Causal forests in the full sample: education results

In this subsection, we report causal forest estimates obtained from the full sample, where educational strata are used as covariates rather than stratifying variables. The aim is to verify that the overall direction of the effects found in our main analysis is also present using this different methodology, lending credibility to heterogeneity results based on causal forests.

First, Figure Q.26 shows the distribution of CITT of both emotional and scientific framing within each educational stratum, by mothers' response to our first survey (our proxy of engagement with materials). Similar to our main analysis results, emotional framing CITT displays more negative values among non-respondents in stratum 3, although the average estimated magnitude is more modest (-0.14). Similarly, scientific framing has a distribution generally shifted towards positive values and, in the case of respondents from stratum 2, the entire interquartile range falls above zero.

Figure Q.26: CITT distributions across educational strata, by survey response

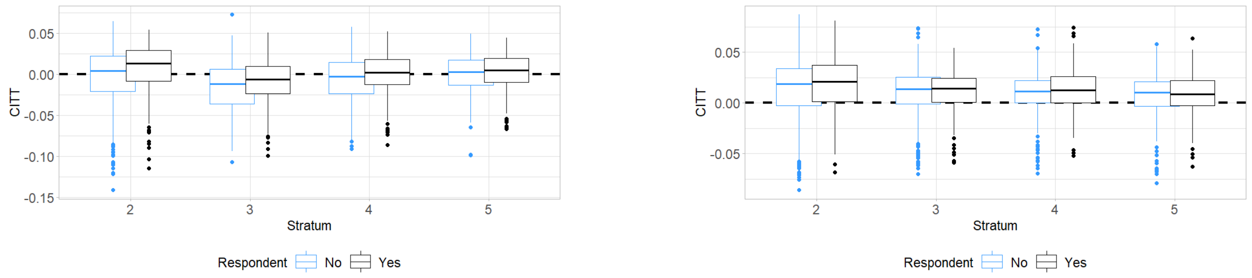

(A.) Emotional framing (T1) **(B.)** Scientific framing (T2)

Notes: The figure shows, separately for the two treatments and strata, boxplots of individual causal effects (CITT). Boxplots are plotted separately for mothers who did and did not reply to our first survey, our proxy of engagement with the leaflets, to maximize comparability with our Logit and OLS estimates. Stratum 1 is not included because the CITT is estimated on the sample of Swedish-born mothers. CITTs are obtained by estimating a causal forest in the full sample of Swedish-born mothers, including educational strata as covariates rather than stratifying variables.

Next, Figure Q.27 andFigure Q.28 replicate Figure 4 and Figure 5 in the main text including educational strata. Importantly, the coefficients of educational strata do not correspond to causal effects and are directly comparable to estimates from the main analysis. They indicate by how

much, on average, CITTs change in a stratum relative to all others, and not the difference between treated and control units in that stratum. With this caveat in mind, their sign and significance are consistent with the main results and add further insights that were not significant at the average level. When comparing mothers with the highest possible education (stratum 5) against all other educational levels, it appears as if their reaction is the opposite of what we found for lowly educated mothers, although magnitudes are small, which suggests that a few, high-leverage observations might drive the association. In other words, postgraduate education from stratum 5 is associated with a higher effect of emotionally framed information (T1) and a lower effect of scientifically framed information (T2). It should be noted that, precisely because the coefficient measures the association of CITT and postgraduate education instead of all other educational levels, the positive estimate also reflects the particularly negative association found in stratum 3 (high school degree holders). Still, we expect highly educated mothers to be already aware of the HPV vaccine and existing campaigns, which could lead them to pay more attention to communication techniques that appear as a novelty to them (T1). Conversely, they might dislike receiving scientifically-framed information they have already encountered (T2). In any case, the policy relevance of these dynamics is limited, since the vaccine uptake in the control group in this educational stratum is already above the recommended coverage of 90%.

Figure Q.27: CITT estimates by baseline covariates and Swedish-born strata: emotional framing (T1)

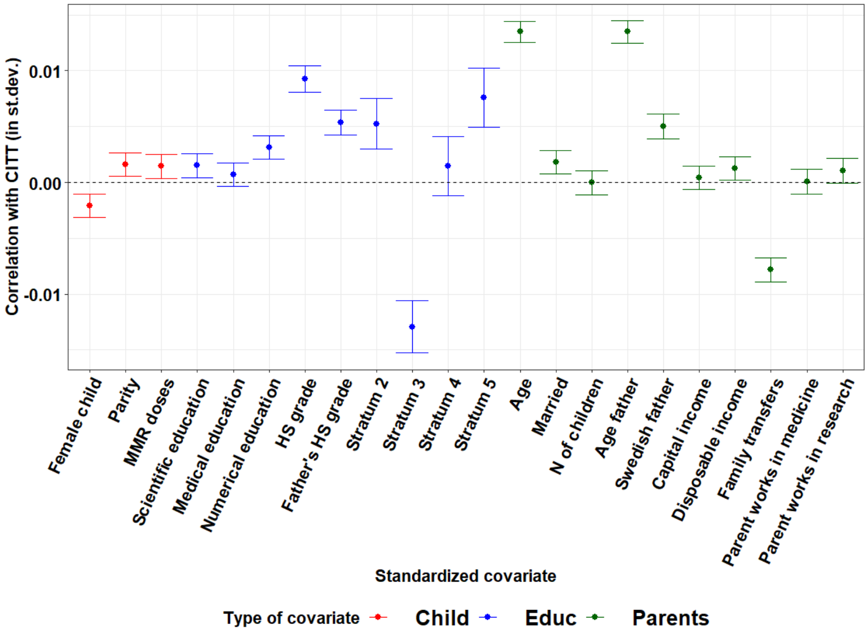

Notes: the figure shows the correlation – estimate and 95% C.I. – between individual CITT estimates from causal forests and baseline covariates, estimated by OLS. Covariates are standardized (i.e., correlations are expressed in standard deviations), except for educational strata, which are dummies.

Figure Q.28: CITT estimates by baseline covariates and Swedish-born strata: scientific framing (T2)

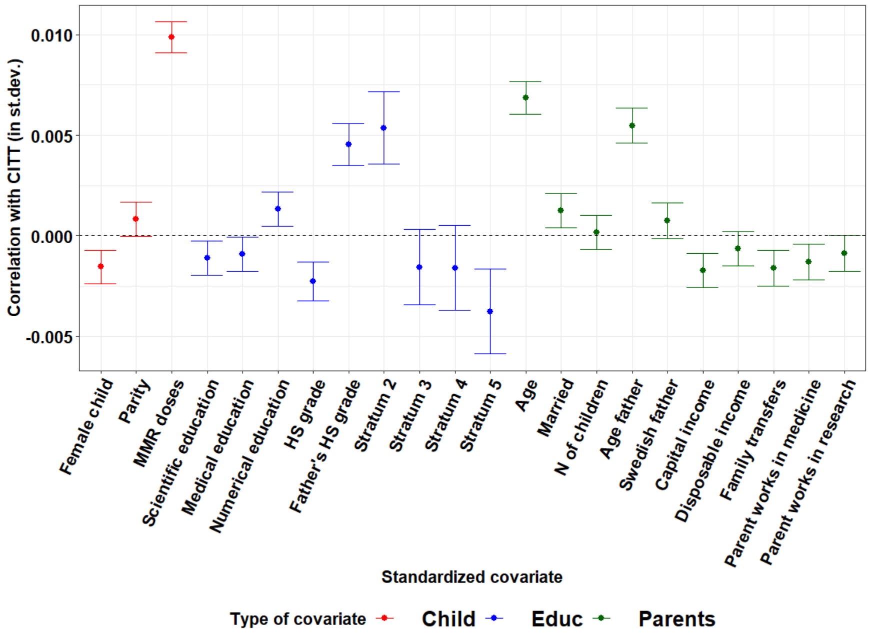

Notes: the figure shows the correlation – estimate and 95% C.I. – between individual CITT estimates from causal forests and baseline covariates, estimated by OLS. Covariates are standardized (i.e., correlations are expressed in standard deviations), except for educational strata, which are dummies.

Q.2 Causal forests estimation within strata

In this section, we report results from estimating causal forest within strata, since (i) the unrestricted causal forest estimates show that strata are important predictors of individual causal effects (CITT) and (ii) the main analysis – as per pre-registration – is conducted within strata. Since the lower sample size within strata imposes higher computational constraints, in this analysis we restrict the forest to 1000 trees.

We include the following covariates: child's gender, birth order among mother's children, the mother's and father's total number of children, mother's age, father's age, mother's civil status (married or not), whether the father is Swedish or not, number of MMR vaccine doses received by the child before treatment, whether the parents' education is specialized in scientific, numerical or medical subjects (two sets of dummies), both parents grade at the national high school's examination (proxied by GPA for parents without a high school degree, on the same scale), whether parents are employed as a researcher or as a medical doctor/nurse/dentist, parent's disposable income, net capital income and transfers in the last fiscal year, whether the mother had heard about HPV at baseline, how much of the leaflet she read, and if any of her close friends or relatives is a medical doctor, and survey variables on the reception and search of HPV information. For immigrant mothers, we also include country of origin dummies, the time since immigration date, the education level, whether they completed any formal degree in Sweden, and if they answered the survey in Swedish to proxy for integration.

To provide more detail, we follow Carlana et al. (2022) in reporting the results. Since the CITT is estimated for each subject, we split the sample in two: the subsample with a CITT equal to or above average and the subsample with a CITT below average. We then test if the difference in means of each covariate in these two subsamples is statistically significant. Given the high number of covariates and the presence of five strata, we only report the covariates that return a statistically significant difference in means at the 95% significance level.

The results concerning Swedish-born mothers reveal some interesting facts about who responds to emotional framing (T1). Emotional framing (T1) is generally more effective on mothers who have a higher labour and capital income, and who read more carefully the leaflet (measured by the self-reported percentage they read, and by whether they answered the first survey). It is less

effective for mothers with a job in healthcare. If the mother has a graduate education (stratum 5), it is more effective when their field of education is not numerically intensive.

Scientific framing (T2) relates to socioeconomic status differently depending on the highest educational attainment. For mothers with just compulsory education, it is more effective when they (and their partners) pursued a more numerical high school track and have a higher income. For mothers with a high school degree (stratum 3) instead, it is more effective when they obtained a lower grade: this is suggestive of both less memory of what was learned in school and worse job prospects. This hypothesis is confirmed by the finding that in stratum 3, scientific framing (T2) is also more effective on mothers with a lower income. Finally, in strata 4 and 5, where mothers have a university education, scientific framing is more effective when both parents have a lower income despite being more likely to be active workers, are less likely to work in research, and less likely to reply to the survey.

To provide a graphical summary of these results, Figure Q.29 and Figure Q.30 show the distribution of the estimated CITT of both treatments by parents' combined income. The figures also reveal that the causal forest estimation confirms the ITT analysis results: scientific framing (T2) has an overall positive impact on uptake in stratum 2 (compulsory education), whereas emotional framing (T1) has a negative impact on uptake in stratum 3 (high school degree). Figure Q.31 and Figure Q.32 provide a visual summary of CITT effects for parents with a medical or research occupation. The average CITT in stratum 2 is highest when at least one parent is a researcher, and is zero when at least one parent is a medical doctor. Since stratum 2 coincides with compulsory education, this sheds light on the role of fathers' education in shaping mothers' opinions. Our causal forest analysis confirms that policy-makers planning informational campaigns face non-linear heterogeneity in the response to framing, characterized by complex interactive effects between education and other parental socioeconomic characteristics.

The same graphical information for mothers with an immigrant background in stratum 1 is reported in Figures Q.33-Q.36. In this case, we find no strong association between causal effects and knowledge of HPV, vaccine attitudes, and trust in health authorities, estimated among respondents to the first survey ($N = 416$). Looking at all the invited immigrant mothers ($N = 2548$), the main predictors of CITTs are countries of origin (with substantial heterogeneity), years since immigration, and education variables. Country dummies indicate that, as suggested by qualitative studies,

immigrant communities require very specific targeting that goes beyond emotional and scientific framing. Education variables, on the other hand, highlight an interesting pattern: more education, more years spent in Sweden (a proxy of integration), and having obtained a school degree in Sweden are associated with higher CITT for emotional framing (T1), similar to Swedish-born mothers, but lower CITT for scientific framing (T2), the opposite of Swedish-born mothers. We interpret this as a possible difficulty in understanding the content of the scientifically framed leaflet. The figures corresponding to this information are presented below.

Figure Q.29: CITT effect of Emotional framing (T1) by parents' income

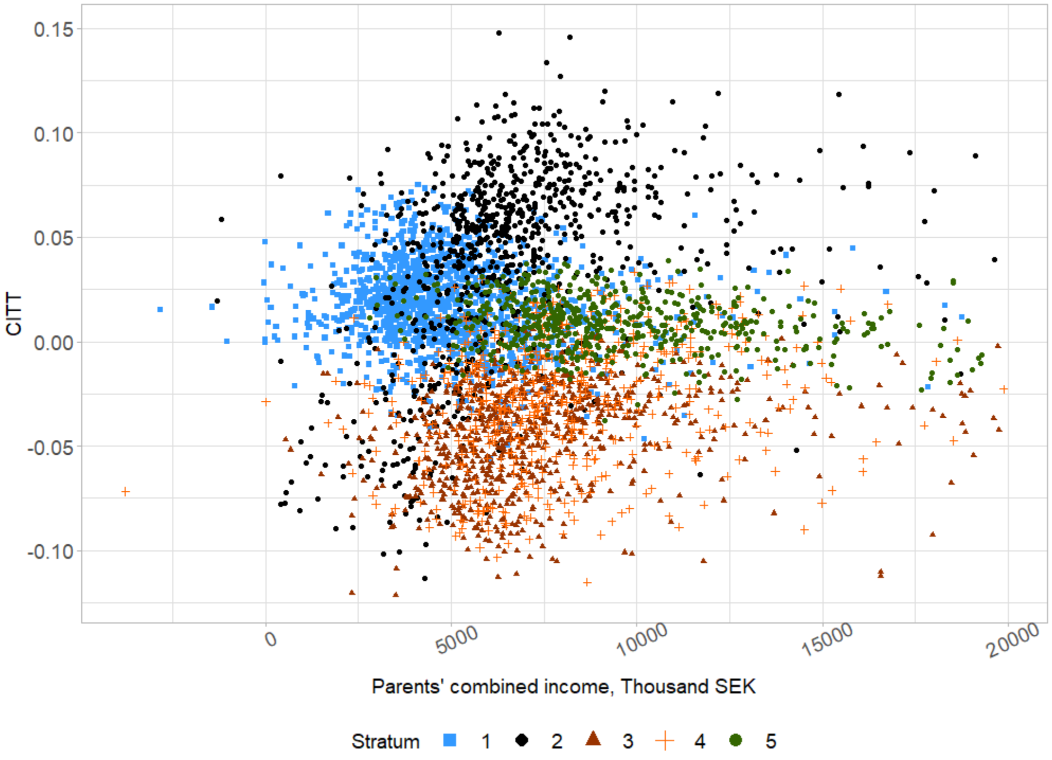

Notes: The figure is a scatterplot with combined parents' income on the x-axis (both capital and labour income) and CITT on the y-axis, restricting to the effect of Emotional framing (T1). Colours of the points indicate the stratum.

Figure Q.30: CITT effect of Scientific framing (T2) by parents' income

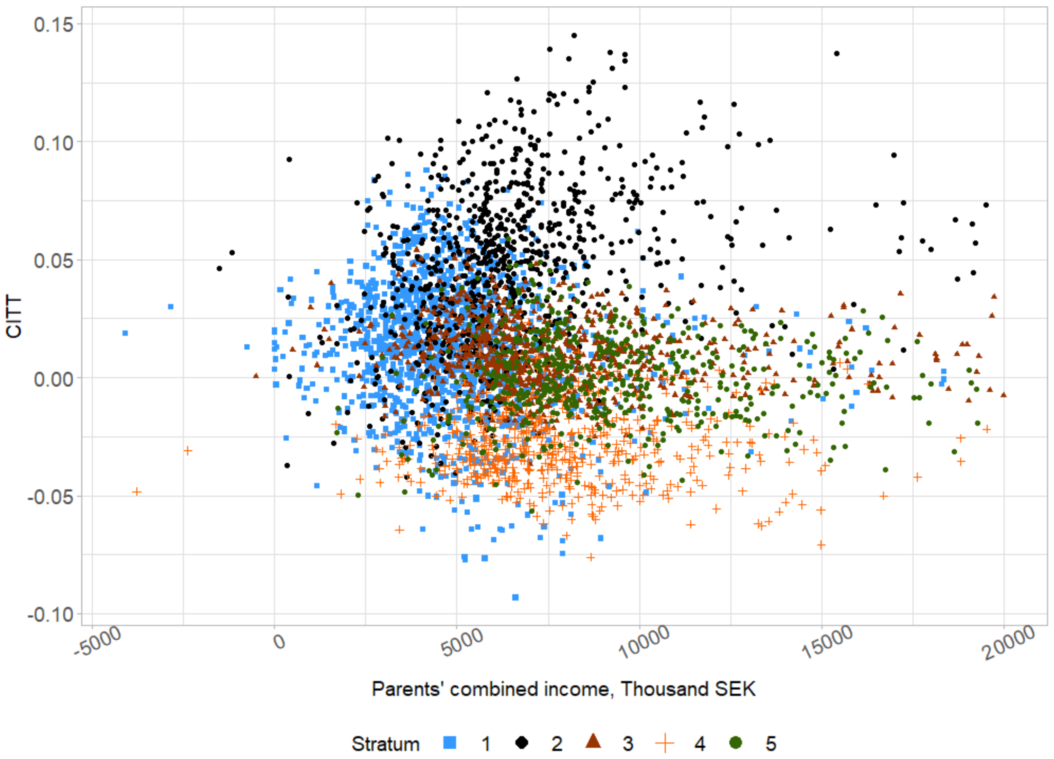

Notes: The figure is a scatterplot with combined parents' income on the x-axis (both capital and labour income) and CITT on the y-axis, restricting to the effect of Scientific framing (T2). Colours of the points indicate the stratum.

Figure Q.31: Mean CITT effect of Emotional framing (T1) by parents' occupation

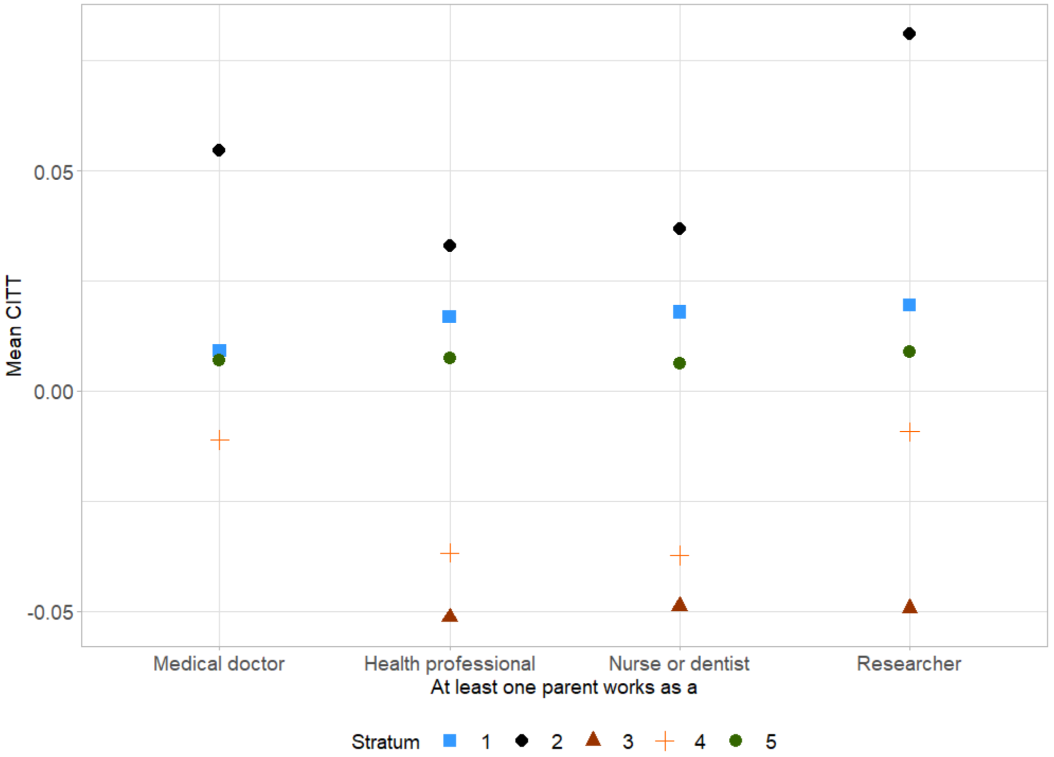

Notes: The figure shows the average CITT of Emotional framing (T1) when at least one parent has a medical or research occupation. Health professionals include medical doctors, nurses and dentists, and other occupations in the health sector. Colours of the points indicate the stratum.

Figure Q.32: Mean CITT effect of Scientific framing (T2) by parents' occupation

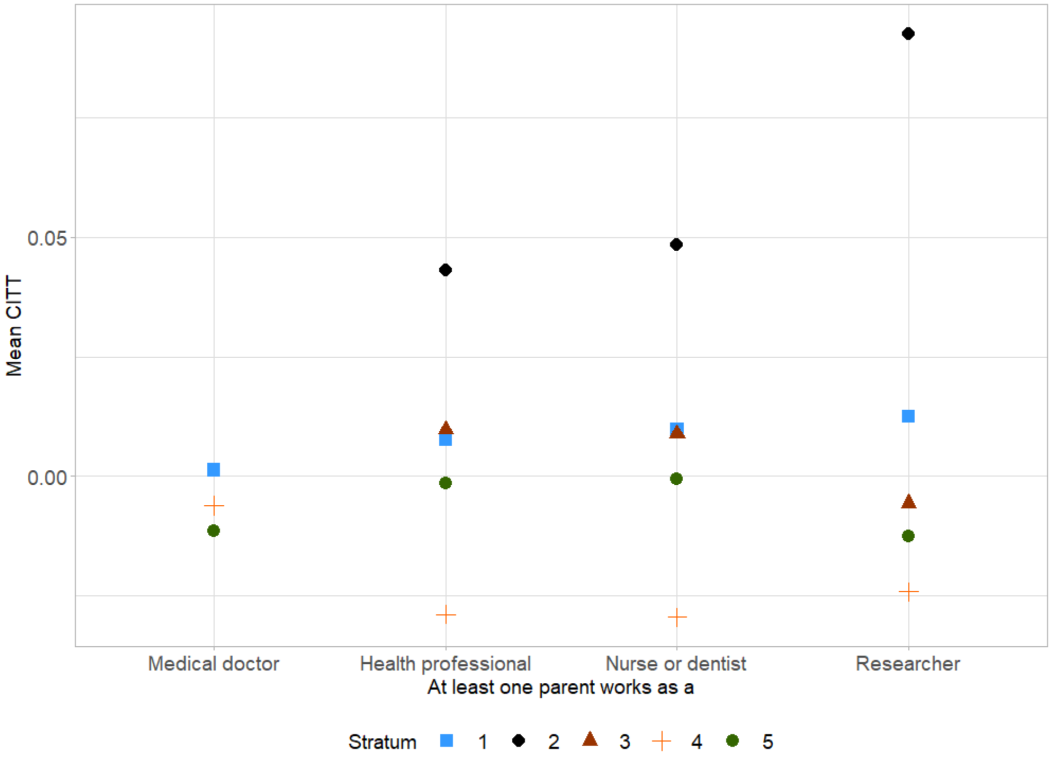

Notes: The figure shows the average CITT of Scientific framing (T2) when at least one parent has a medical or research occupation. Health professionals include medical doctors, nurses and dentists, and other occupations in the health sector. Colours of the points indicate the stratum.

Figure Q.33: CITT estimates in stratum 1 by baseline covariates: emotional framing (T1)

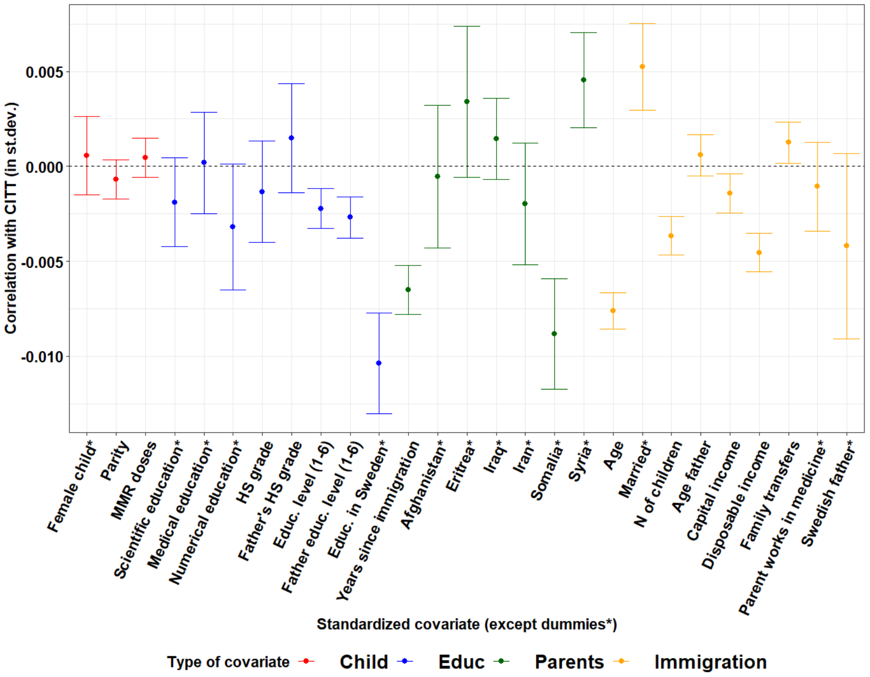

Notes: the figure shows the correlation – estimate and 95% C.I. – between individual CITT estimates from causal forests and baseline covariates, estimated by OLS for all invited mothers in stratum 1 ($N = 2548$). Covariates are standardized (i.e., correlations are expressed in standard deviations), except for dummy variables, denoted by an asterisk.

Figure Q.34: CITT estimates in stratum 1 by survey answers: emotional framing (T1)

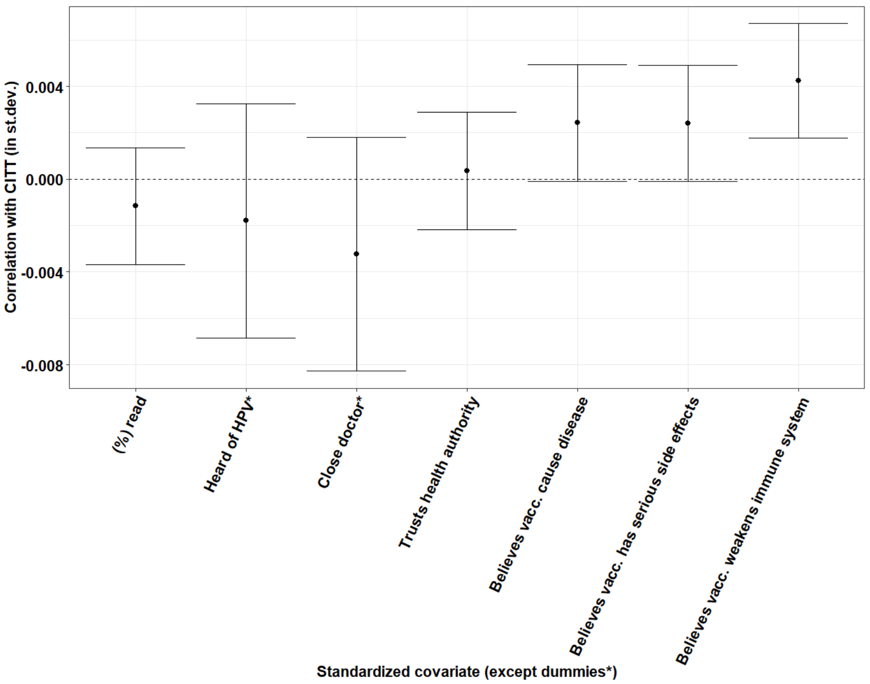

Notes: the figure shows the correlation – estimate and 95% C.I. – between individual CITT estimates from causal forests and answers to the first survey, estimated by OLS among respondents in stratum 1 ($N = 416$). Covariates are standardized (i.e., correlations are expressed in standard deviations), except for dummy variables, denoted by an asterisk.

Figure Q.35: CITT estimates in stratum 1 by baseline covariates: scientific framing (T2)

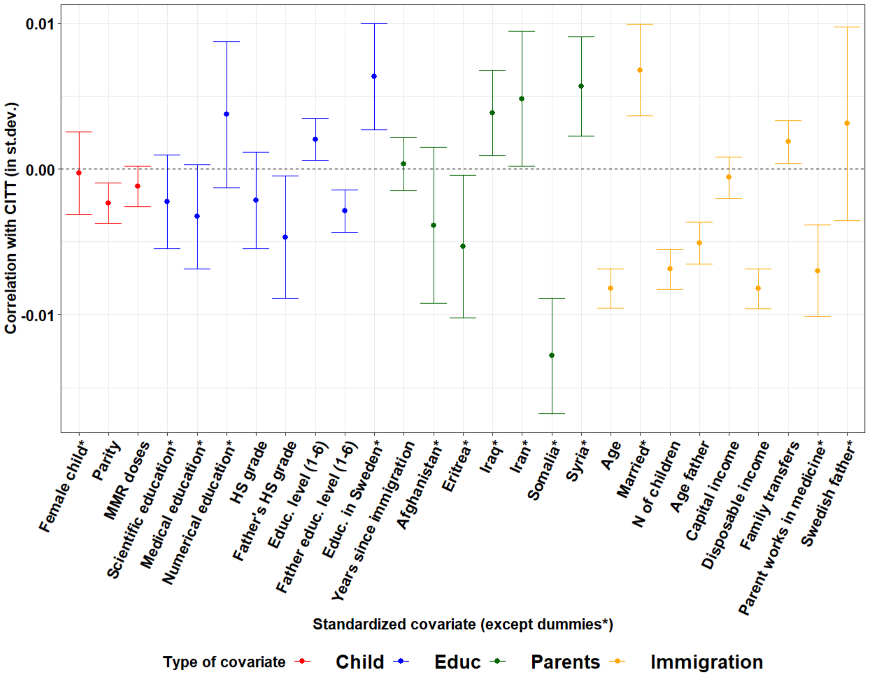

Notes: the figure shows the correlation – estimate and 95% C.I. – between individual CITT estimates from causal forests and baseline covariates, estimated by OLS for all invited mothers in stratum 1 ($N = 2548$). Covariates are standardized (i.e., correlations are expressed in standard deviations), except for dummy variables, denoted by an asterisk.

Figure Q.36: CITT estimates in stratum 1 by survey answers: scientific framing (T2)

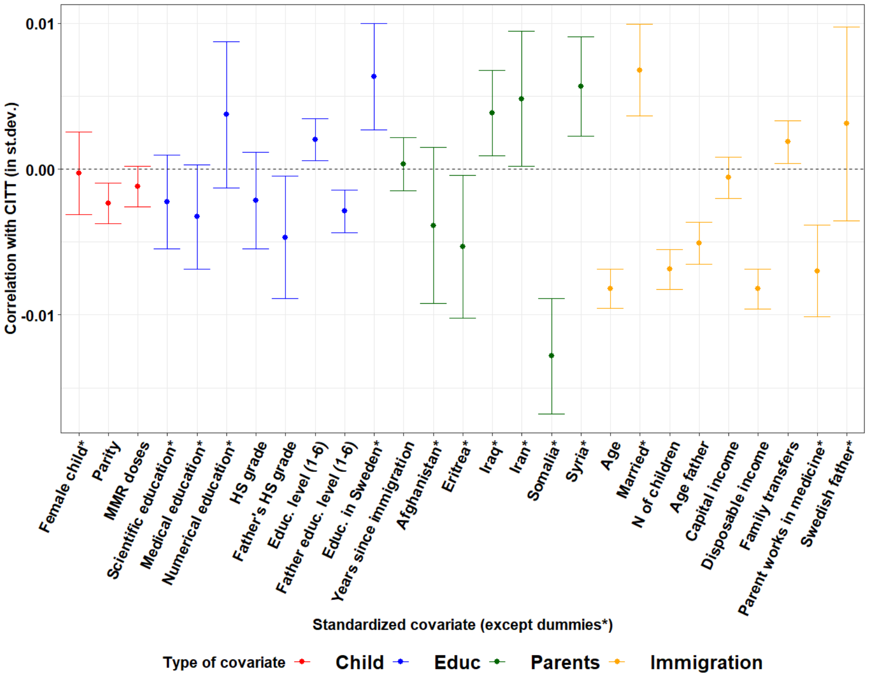

Notes: the figure shows the correlation – estimate and 95% C.I. – between individual CITT estimates from causal forests and answers to the first survey, estimated by OLS among respondents in stratum 1 ($N = 416$). Covariates are standardized (i.e., correlations are expressed in standard deviations), except for dummy variables, denoted by an asterisk.

Causal forest: Conditional ITT of Emotional framing (T1) on actual vaccination

Stratum	Variable (red for respondents only)	Mean high CITT	Mean low CITT	diff. means
1	Father's education level	3.043	3.474	-0.431∗∗∗
1	Father has scientific education	0.268	0.344	-0.077∗∗∗
1	Father is a medical doctor	0.043	0.071	-0.028∗∗
1	Father has numerical education	0.118	0.155	-0.037∗∗
1	Father works as a researcher	0.006	0	0.006∗∗
1	Father is married	0.843	0.771	0.071∗∗∗
1	Father is from a western country	0.039	0.062	-0.024∗∗
1	Father is from a Muslim majority country	0.834	0.748	0.086∗∗∗
1	Father is an immigrant	0.974	1	-0.026∗∗
1	Mother's education level	3.106	3.635	-0.529∗∗∗
1	Mother's yearly income	2174.798	2558.384	-383.586∗∗∗
1	Mother's age	37.479	41.111	-3.633∗∗∗
1	Mother's number of children	2.757	3.165	-0.407∗∗∗
1	Mother is married	0.764	0.668	0.096∗∗∗
1	Year of immigration	2003.745	1998.573	5.172∗∗∗
1	Time since immigration	17.224	22.397	-5.174∗∗∗
1	Completed formal degree in Sweden	0.354	0.579	-0.224∗∗∗
1	Mother is from Somalia	0.102	0.184	-0.081∗∗∗
1	Mother from Syria	0.244	0.185	0.059∗∗∗

1	Parents have some education in Sweden	0.849	0.932	-0.083∗∗∗
	**Survey variables observed among respondents - Stratum 1**			
1	*Believes vaccines weaken the immune system*	2.754	2.344	0.41∗∗
1	*Has been exposed to a previous informational campaign on HPV*	0.31	0.43	-0.12∗∗

Stratum	Variable	Mean high CITT	Mean low CITT	diff. means
2	Father's education level	3.402	2.902	0.501∗∗∗
2	Father has numerical education	0.205	0.105	0.1∗∗∗
2	Father's high school degree grade	12.436	11.029	1.407∗∗∗
2	Father's high school graduation year	1993.044	1997.549	-4.504∗∗∗
2	Father is an active worker	0.962	0.846	0.117∗∗∗
2	Father receives transfers	0.013	0.105	-0.092∗∗∗
2	Father's age	45.622	40.735	4.886∗∗∗
2	Father is married	0.55	0.369	0.181∗∗∗
2	Father is from a western country	0.803	0.686	0.117∗∗∗
2	Mother's education level	2.793	2.57	0.223∗∗∗
2	Mother is a medical doctor	0.114	0.158	-0.044∗∗
2	Mother has a numerical education	0.156	0.059	0.096∗∗∗
2	Mother is a nurse/dentist	0.109	0.158	-0.049∗∗
2	Mother's high school grade	12.894	3.87	9.024∗∗∗
2	Mother's high school graduation year	1991.206	2001.347	-10.141∗∗∗
2	Mother's yearly income	3512.756	2258.717	1254.039∗∗∗

2	Mother is an active worker	0.969	0.533	0.436***
2	Mother is retired	0	0.01	-0.01**
2	Mother receives government transfers	0.007	0.392	-0.385***
2	Mother has a medical occupation	0.153	0.221	-0.068***
2	Mother's age	43.737	37.114	6.623***
2	Mother is married	0.502	0.328	0.173***
2	Mother replied to the survey	0.268	0.173	0.095***
2	Total transfers received by parents	0.017	0.467	-0.45***
2	Any of the parents has another occupation in healthcare	0.192	0.275	-0.083***
2	Any of the parents is retired	0.002	0.028	-0.026***
	**Survey variables observed among respondents - Stratum 2**			
2	*% of leaflet read*	82.9	73.29	9.62**
2	*Trusts health authorities*	4.327	3.943	0.384**

Stratum	Variable	Mean high CITT	Mean low CITT	diff. means
3	Father's education level	3.881	2.995	0.886***
3	Father has numerical education	0.318	0.148	0.17***
3	Father's high school degree grade	13.277	10.054	3.222***
3	Father's high school graduation year	1993.078	1998.017	-4.938***
3	Father's capital income	638.964	109.527	529.437**
3	Father's yearly income	5451.787	4050.743	1401.043***
3	Father receives transfers	0.016	0.046	-0.031**

3	Father's age	45.779	41.003	4.777***
3	Father is married	0.619	0.529	0.091***
3	Father is from a western country	0.832	0.761	0.071***
3	Father is from a Muslim majority country	0.038	0.076	-0.038**
3	Mother's education level	3.004	3.03	-0.026**
3	Mother's high school grade	12.879	11.765	1.114***
3	Mother's high school graduation year	1996.182	2000.266	-4.084***
3	Mother's yearly income	3789.535	3014.484	775.051***
3	Mother is an active worker	0.922	0.851	0.071***
3	Mother receives government transfers	0.069	0.126	-0.056***
3	Mother has a medical occupation	0.086	0.161	-0.075***
3	Mother's age	42.676	39.01	3.666***
3	Mother is married	0.571	0.489	0.082**
3	Mother replied to the survey	0.408	0.305	0.104***
3	Total transfers received by parents	0.076	0.169	-0.093***
3	Any of the parents has another occupation in healthcare	0.108	0.184	-0.076***
	**Survey variables observed among respondents - Stratum 3**			
3	*Has searched HPV information before treatment*	0.407	0.529	-0.122**
3	*Believes vaccines cause the disease they should avoid*	1.711	1.95	-0.239**
3	*Trusts health authorities*	4.51	4.231	0.279***

Stratum	Variable	Mean high CITT	Mean low CITT	diff. means
4	Birth order	1.038	1.01	0.028**
4	Father's education level	4.097	3.653	0.444***
4	Father's high school degree grade	13.841	12.162	1.679***
4	Father's high school graduation year	1993.223	1995.77	-2.547***
4	Father's age	45.756	43.19	2.566***
4	Father is from a Muslim majority country	0.012	0.049	-0.037***
4	Mother's high school grade	15.685	11.505	4.18***
4	Mother's high school graduation year	1995.12	1997.712	-2.592***
4	Mother's capital income	305.871	-219.77	525.641***
4	Mother's yearly income	4377.141	3321.551	1055.59***
4	Mother is an active worker	0.906	0.84	0.066**
4	Mother has a medical occupation	0.044	0.108	-0.064***
4	Mother's age	43.985	41.366	2.619***
4	Mother replied to the survey	0.484	0.362	0.122***
4	Any of the parents has another occupation in healthcare	0.069	0.126	-0.057**

Stratum	Variable	Mean high CITT	Mean low CITT	diff. means
5	Father's high school degree grade	14.95	14.069	0.881***
5	Father's high school graduation year	1993.004	1994.53	-1.526***
5	Father's age	45.823	44.116	1.707***
5	Mother has a numerical education	0.241	0.317	-0.077**
5	Mother is a medical doctor	0.022	0.052	-0.03**

5	Mother's high school grade	15.036	15.703	-0.667∗∗∗
	**Survey variables observed among respondents - Stratum 5**			
5	*% of leaflet read*	82.29	69.87	12.41∗∗∗

Notes: *** $p < 0.01$, ** $p < 0.05$. The table shows the mean of baseline covariates for observations that have a CITT above average, the mean of baseline covariates for observations with a CITT below average, and a test for the difference in means. We restrict to variables where the difference is statistically different at the 95% s.l. for readability. Survey variables that are only observed among respondents are reported at the end of each stratum's table in red: they are not included as covariates in the causal forest estimation, only compared among respondents with above and below-average CITT. Immigrants (stratum 1) are mothers born in Iraq, Iran, Syria, Afghanistan, Eritrea or Somalia. Stratum 2 comprises mothers with at most 3 years of high school: this corresponds to Swedish *högstadiet* (grades 7-9), the last compulsory grades under Swedish law. Mothers in stratum 3 completed high school (*gymnasium*, grades 10-12), which is not compulsory and comprises different tracks, including vocational ones. Stratum 4 comprises mothers with some undergraduate education, and stratum 5 mothers with some graduate education.

Causal forest: Conditional ITT of Scientific framing (T2) on actual vaccination

Stratum	Variable	Mean high CITT	Mean low CITT	diff. means
1	Doses of MMR vaccine received before treatment	1.703	1.749	-0.046**
1	Father's education level	3.185	3.464	-0.279***
1	Father is a medical doctor	0.008	0.027	-0.019***
1	Father has another occupation in healthcare	0.076	0.114	-0.038**
1	Father's age	44.529	46.672	-2.143***
1	Father's number of children	2.662	2.858	-0.196**
1	Father is married	0.844	0.797	0.048**
1	Father is from a Muslim majority country	0.825	0.77	0.055***
1	Father is born in Sweden	0.023	0	0.023**
1	Mother's high school graduation year	2002.028	2000.444	1.583**
1	Mother's yearly income	2171.001	2711.842	-540.84***
1	Mother is an active worker	0.548	0.601	-0.053**
1	Mother has a medical occupation	0.216	0.267	-0.051**
1	Mother's age	38.344	40.835	-2.49***
1	Mother's number of children	2.773	3.354	-0.581***
1	Mother is married	0.765	0.689	0.076***
1	Completed formal degree in Sweden	0.504	0.401	0.103***
1	Mother from Eritrea	0.077	0.106	-0.029**
1	Mother is from Somalia	0.116	0.179	-0.062***

1	Mother from Syria	0.238	0.196	0.042∗∗
1	Any of the parents has another occupation in healthcare	0.279	0.357	-0.078∗∗∗
	**Survey variables observed among respondents - Stratum 1**			
1	*Has close friends/relatives who are doctors*	0.425	0.566	-0.141∗∗

Stratum	Variable	Mean high CITT	Mean low CITT	diff. means
2	Birth order	1.05	1.02	0.03∗∗∗
2	Doses of MMR vaccine received before treatment	1.902	1.807	0.095∗∗∗
2	Father's education level	3.367	3.037	0.33∗∗∗
2	Father has numerical education	0.241	0.139	0.102∗∗∗
2	Father's high school degree grade	12.431	11.599	0.832∗∗
2	Father's high school graduation year	1992.542	1997.761	-5.22∗∗∗
2	Father is an active worker	0.935	0.884	0.051∗∗
2	Father receives transfers	0.047	0.092	-0.044∗∗
2	Father's age	45.87	41.237	4.633∗∗∗
2	Father is married	0.535	0.411	0.124∗∗∗
2	Father is from a western country	0.817	0.654	0.162∗∗∗
2	Mother's education level	2.822	2.6	0.223∗∗∗
2	Mother has a numerical education	0.181	0.061	0.12∗∗∗
2	Mother's high school grade	12.593	6.986	5.607∗∗∗
2	Mother's high school graduation year	1990.891	1998.095	-7.204∗∗∗
2	Mother's capital income	-200.676	65.586	-266.262∗∗∗

2	Mother's yearly income	3409.541	2672.049	737.491***
2	Mother is an active worker	0.877	0.687	0.19***
2	Mother receives government transfers	0.103	0.241	-0.138***
2	Mother has a medical occupation	0.153	0.237	-0.084***
2	Mother's age	44.332	37.868	6.464***
2	Mother is married	0.498	0.335	0.163***
2	Mother replied to the survey	0.237	0.187	0.05**
2	Total transfers received by parents	0.129	0.324	-0.195***
2	Any of the parents has another occupation in healthcare	0.19	0.296	-0.106***

Stratum	Variable	Mean high CITT	Mean low CITT	diff. means
3	Doses of MMR vaccine received before treatment	1.949	1.902	0.047***
3	Father's high school graduation year	1994.093	1995.791	-1.698***
3	Mother's high school grade	11.744	12.261	-0.517**
3	Mother's capital income	-176.626	216.955	-393.581***
3	Mother's yearly income	3143.679	3928.667	-784.988***
	**Survey variables observed among respondents - Stratum 3**			
3	*% of leaflet read*	73.65	81.23	-7.58**
3	*Want to search HPV information from untrustworthy sources after treatment*	0.183	0.097	0.086**

Stratum	Variable	Mean high CITT	Mean low CITT	diff. means
4	Doses of MMR vaccine received before treatment	1.946	1.896	0.05**

4	Father's education level	4.004	3.746	0.258***
4	Father has numerical education	0.364	0.241	0.123***
4	Father is a medical doctor	0.018	0	0.018**
4	Father's high school degree grade	14.358	11.911	2.448***
4	Father's high school graduation year	1992.878	1995.906	-3.028***
4	Father's age	46.093	43.264	2.829***
4	Father is married	0.621	0.537	0.084**
4	Mother's high school grade	14.187	13.382	0.805***
4	Mother's high school graduation year	1994.836	1998.174	-3.338***
4	Mother's capital income	-69.899	190.384	-260.283**
4	Mother is an active worker	0.909	0.826	0.083***
4	Mother's age	44.125	40.902	3.223***

Stratum	Variable	Mean high CITT	Mean low CITT	diff. means
5	Birth order	1.02	1.061	-0.041**
5	Father's education level	4.369	4.579	-0.21***
5	Father's capital income	600.451	2519.909	-1919.458**
5	Mother's education level	5.017	5.058	-0.041***
5	Mother's high school grade	13.856	16.828	-2.972***
5	Mother's yearly income	4270.257	5306.508	-1036.25**
5	Mother is an active worker	0.964	0.918	0.046**
5	Mother is a researcher	0.003	0.036	-0.033***
5	Mother replied to the survey	0.462	0.55	-0.088**

5	Some parent is a researcher	0.017	0.057	-0.04***

Notes: *** p< 0.01, ** p< 0.05. The table shows the mean of baseline covariates for observations that have a CITT above average, the mean of baseline covariates for observations with a CITT below average, and a test for the difference in means. We restrict to variables where the difference is statistically different at the 95% s.l. for readability. Survey variables that are only observed among respondents are reported at the end of each stratum's table in italic: they are not included as covariates in the causal forest estimation, only compared among respondents with above and below-average CITT. Immigrants (stratum 1) are mothers born in Iraq, Iran, Syria, Afghanistan, Eritrea or Somalia. Stratum 2 comprises mothers with at most 3 years of high school: this corresponds to Swedish *högstadiet* (grades 7-9), the last compulsory grades under Swedish law. Mothers in stratum 3 completed high school (*gymnasium*, grades 10-12), which is not compulsory and comprises different tracks, including vocational ones. Stratum 4 comprises mothers with some undergraduate education, and stratum 5 mothers with some graduate education.

R Endline survey

The second survey is administered at endline, i.e. after the vaccinations took place. Its text can be read in Section F.2 of this Appendix. Only mothers who replied to the first survey are invited to participate in the second. Table R.40 shows, for each stratum, the number of respondents to the first survey (in round brackets, in blue), and the number of respondents to the second survey (in square brackets, in red).

The aim of the second survey is to investigate additional mechanisms of our effects and measure a self-reported indicator of vaccination status. However, the reduced sample size implies that any evidence from the second survey should be interpreted as qualitative and merely suggestive.

For what concerns mechanisms, based on these data we find that the only concern affected relates to the vaccine's safety. Figure R.37 shows that in stratum 2 scientific framing (T2) – which is also effective on average – reduces the wrong perception that the vaccine might increase the risk of having to recur to invasive medical procedures. This is a dimension of vaccine safety concerns that is directly tackled by our information. The concern might be that medical invasive procedures might be required following vaccine adverse effects, and we shift the focus to those required following HPV-induced cancer that the vaccine can prevent. Concerns about the effect of the vaccine on fertility or the emergence of cancer and serious illness are not affected by treatment in this specific subsample.

Table R.39 and Table R.38 report the joint distribution of the self-reported vaccination status from the second survey and (i) the intention to vaccinate expressed in the first survey; (ii) the actual vaccination record from administrative data.

Table R.38: Self-reported vaccination status and actual vaccination status

Self-reported	Actual vaccination record		
	Not vaccinated	Vaccinated	Inconsistent answers
"Did you vaccinate your child against HPV earlier this fall?"			
No	18	1	10 (5.263%)
I am not sure Probably not	0	3	3 (100%)
I am not sure Probably yes	0	10	0 (0%)
Yes	15	652	15 (2.249%)

The table reports the joint distribution of self-reported vaccination status in the second survey (administered at endline) and the actual vaccination status from administrative records.

Table R.39: Self-reported vaccination status and intention to vaccinate

Self-reported	Willingness to vaccinate		
	Does not intend to vaccinate	Intends to vaccinate	Inconsistent answers
"Did you vaccinate your child against HPV earlier this fall?"			
No	12	7	7 (36.842%)
I am not sure Probably not	1	2	2 (66.670%)
I am not sure Probably yes	2	8	2 (0.200%)
Yes	29	638	29 (4.348%)

The table reports the joint distribution of self-reported vaccination status in the second survey (administered at endline) and the willingness to vaccinate expressed in the first survey (immediately after treatment).

Figure R.37: CITT of scientific framing (T2) by beliefs on vaccine safety

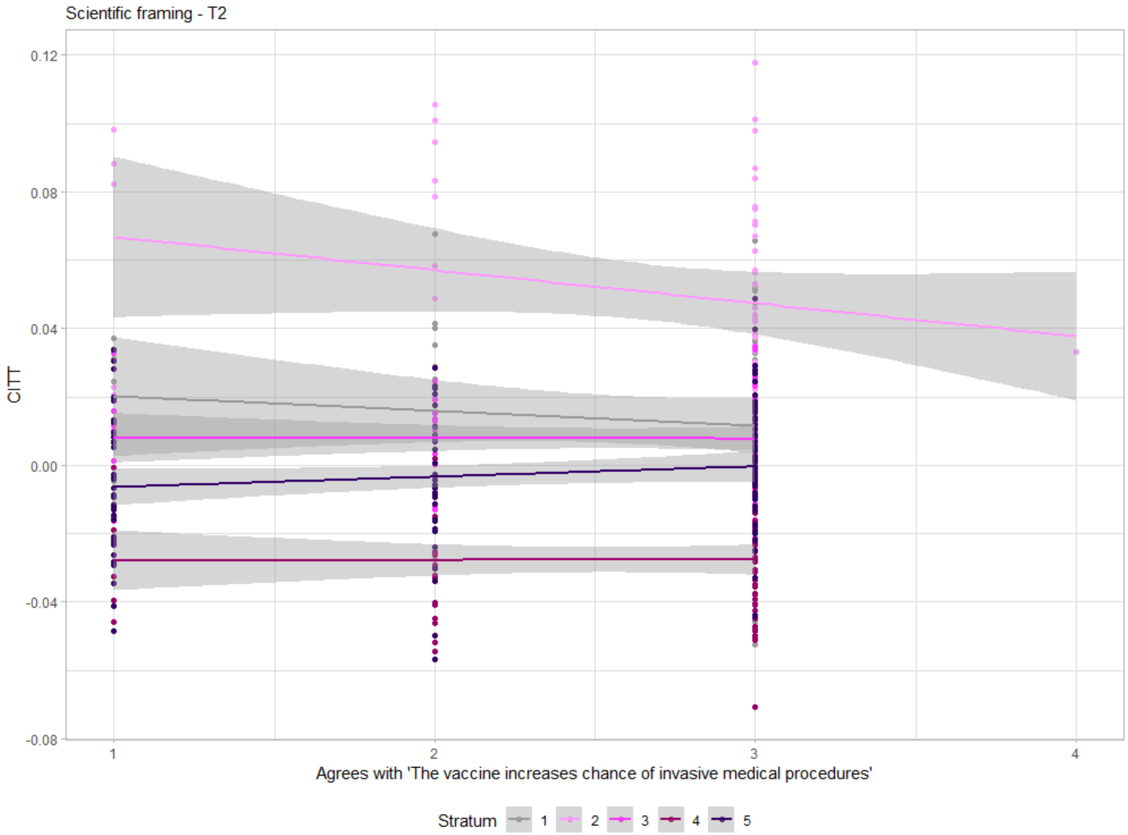

Notes: The figures shows the Conditional ITT of scientific framing (T2) by the agreement with statement "Vaccines increase the chance of invasive medical procedures". The values on the x-axis correspond to: (1) "Do not agree"; (2) "Partly disagree"; (3) "Neither disagree nor agree"; (4) "Partly agrees".

Table R.40: Sample sizes: full sample and survey respondents

Stratum	Stratum definition	N	C units Placebo	T1 units Emotional	T2 units Scientific
1. Immigrants	Selected origin countries	2548	611	961	976
		(416)	(106)	(148)	(162)
		[96]	[14]	[38]	[44]
**Swedish-born mothers**					
2. Educ-level-1	$\leq$ 3 yrs high school	1627	393	616	617
	End of compulsory schooling	(353)	(94)	(138)	(121)
		[101]	[30]	[38]	[33]
3. Educ-level-2	(3 yrs high school, high school degree]	1413	337	535	541
		(484)	(112)	(203)	(169)
		[137]	[28]	[61])	[48]
4. Educ-level-3	(High school degree, Undergrad]	1009	243	385	381
		(417)	(101)	(168)	(148)
		[144]	[26]	[65]	[53]
5. Educ-level-4	> Undegrad degree	1019	242	387	390
		(534)	(122)	(213)	(199)
		[221]	[51]	[85]	[85]
**Total**		7616	1826	2884	2905
		(2204)	(535)	(870)	(799)
		[699]	[149]	[287]	[263]

Notes: Numbers in round brackets indicate respondents to the first survey. Numbers in square brackets indicate respondents to the second survey. Immigrants are mothers born in Iraq, Iran, Syria, Afghanistan, Eritrea or Somalia. Stratum 2 comprises mothers with at most 3 years of high school: this corresponds to Swedish *högstadiet* (grades 7-9), the last compulsory grades under Swedish law. Mothers in stratum 3 completed high school (*gymnasium*, grades 10-12), which is not compulsory and comprises different tracks, including vocational ones.

S Cost-Benefit estimates

Cost-benefit estimates of implementing a targeted leaflet campaign cannot prescind from our final policy recommendations. Specifically with respect to framing, we recommend to: (i) Target scientifically framed information to lowly educated parents and (ii) Avoid emotional framing: while the benefits are negligible and only concern small subgroups that already displayed sufficient HPV vaccine coverage before our intervention, emotional framing reduces the uptake among the lowly educated, who start from sub-optimal coverage.

Importantly, our results find no recommendations for differential framing by education. In other words, we do not find evidence that it is beneficial to address different types of framing to different educational backgrounds, but rather that scientific framing prevails regardless of education, and should be targeted in particular to the less educated. This greatly simplifies logistic, hence monetary costs. Since leaflet-based and population-wide informational campaigns are already in place, especially in Sweden (for instance, the school reminder included in Section A of the Appendix contains some information on the vaccination program and HPV), following our recommendations would only require replacing the text with a scientifically framed one that covered the topics we covered, regardless of targeting. Alternatively, one could devise a targeted leaflet campaign for lower socio-economic strata following the same strategy we used for this study: in Sweden and other Nordic countries, it's enough to use population registers, which is free for governmental authorities, whereas other countries could cross-reference census data with postal zip codes, especially where catchment areas are in place.

In any case, the ubiquitous better – or at least, non-harmful – performance of scientific framing across educational strata implies that targeting the less educated is only a matter of cutting implementation costs: this is not necessary in all those contexts where some form of informational campaign is already in place and can be adapted in terms of framing.

In terms of actual cost-benefit analysis, the benefits are difficult to quantify because they should include the lower expenditures for cancer screenings programs, cancer care (which includes several kinds of therapies), and the additional contribution to the labour force from avoided cancer cases. While conducting this type of analysis in the Stockholm County is not possible in the absence of specific data, we can rely on OECD estimates for the whole of Sweden, which also allows us

to compare Swedish figures with OECD averages. The OECD estimates that if HPV vaccine coverage were at the recommended threshold of 90% between 2023 and 2050, in Sweden, cancer-related costs would fall by 4 EUR PPP per capita, and workforce output would increase by 6 EUR PPP per capita, because of the avoided cases of HPV-induced cancer – the corresponding average OECD figures are 3 and 6.4 (Organisation for Economic Co-operation and Development, 2024). Considering the the entire Swedish population is approximately 10 millions, this amounts to saving 100 million EUR PPP. The per capita cost of mailing a leaflet in the absence of bulk mailing (i.e., the most conservative cost estimate) is 15 SEK per mail, equivalent to approximately 1.38 EUR at the time of writing. The total population of children below 14 – whose coverage roughly corresponds to the 2023-2050 period in perspective – amounts to 1.778.468 individuals (Statistics Sweden, 2024). Without targeting, without bulk mailing, and assuming constant mailing prices, the total expenditure for a scientific leaflet campaign that addresses all children currently below age 14 would be 2.454 million EUR, plus the one-time cost of designing the leaflet, which should, however, be covered by existing Public Health Agency expenditures. We cannot quantify clearly the costs due to vaccine procurement, but we hypothesize they are not a large cost component in perspective: first, because Sweden already procures a number of doses close to the 90% target; second, because the HPV vaccine (Gardasil9) patent will expire in 2030, which will dramatically cut the vaccine acquisition costs. Therefore, reaching the 90% target implies a benefits-to-costs ratio of at least 40, in monetary terms, assuming to run a leaflet campaign addressed to all mothers of children who are currently less than 14, when they reach vaccination age. Costs are further cut down if the campaign is addressed only to lowly educated mothers who reacted positively to scientifically framed information.

The remaining question is: does our campaign enable to reach the 90% target? Table S.41 reports the weight of each stratum in the Stockholm County population, their uptake of the HPV vaccine in the control group, and scenarios of changes in uptake following a targeted campaign addressed only to the stratum where we found statistically significant ITT estimates. We find that addressing scientific framing to stratum 2 brings coverage from 88.4% to 88.9% (1 percentage point away from the 90% target), whereas addressing emotional framing to stratum 3 brings overall coverage down to 87.5%.

These changes might seem small because in Stockholm County, mothers of 12 year olds in educational strata 2 and 3 constitute 9% and 18.5% of the mothers' population of interest, respectively.

However, Stockholm County is on average more educated than the rest of the country – that's part of the reason we based our experiment there, to observe enough highly educated mothers. Looking at all women who were between 40 and 52 in 2021 (roughly the same age as mothers in our study) in all Sweden, those with stratum 2 education were 10%, only slightly higher than our setting, but those with stratum 3 education were 45%, many more (Statistics Sweden, 2022). On the other hand, women in that age group with postgraduate education are approximately 19% of the total, rather than the 47% in our sample. While we cannot run the full scenario for all Sweden because missing education data implies that weights computed from Statistics Sweden (2022) do not add up to one, we believe it is reasonable to assume that a scientifically framed campaign would increase coverage to 90% or close.

Conversely, an emotionally framed campaign would lower coverage much more than in the Stockholm County, implying additional annexed healthcare costs. Highlighting the negative effects of emotionally framed information is just as important as promoting effective campaigns. Besides the non-governmental organization of Swedish cancer survivors we took testimonies from in drafting our emotionally framed leaflets, emotional framing is advocated and used for informational campaigns by NGOs and health authorities in several countries worldwide. For instance, emotional survivor testimonies are used by U.S. state and federal health authorities (e.g., Minnesota Department of Health, 2024; Centers for Disease Control and Prevention, 2024), by WHO agencies (Pan American Health Organization, 2024), by global advocacy groups, collecting testimonies from all continents (Cervivor, Inc., 2025), and by NGOs (e.g., for two authoritative examples from the UK and Australia, see Cancer Research UK, 2024; Cancer Council Victoria, 2024).

Table S.41: Weighted Coverage by Scenario and Stratum

Stratum	Pop. weight	Uptake C group	T1 scenario	T2 scenario
1	0.139	0.773	0.773	0.773
2	0.090	0.786	0.786	**0.843**
3	0.185	0.887	**0.839**	0.887
4	0.119	0.905	0.905	0.905
5	0.467	0.930	0.930	0.930
**Weighted coverage**		**0.884**	**0.875**	**0.889**

Note: Values in bold indicate adjusted coverage in alternative scenarios where targeted leaflet campaigns are only addressed to strata where we found a statistically significant average effect. Population weights are relative to the Stockholm County in 2021, and were computed by *Statistics Sweden* for the purpose of this study.
